# From knowledge to action: the importance of characterizing herbicide resistance mechanisms for implementing management strategies

**DOI:** 10.1002/ps.71187

**Published:** 2026-08-13

**Authors:** Luan Cutti, Eric L. Patterson, Todd A. Gaines, Aldo Merotto

**Affiliations:** ^1^ Department of Crop Science Federal University of Rio Grande do Sul Porto Alegre RS Brazil; ^2^ Department of Plant, Soil, and Microbial Sciences Michigan State University East Lansing MI USA; ^3^ Department of Agricultural Biology Colorado State University Fort Collins CO USA

**Keywords:** target site resistance, non‐target site resistance, herbicide rotation, resistance diagnosis, resistance management

## Abstract

Herbicide resistance remains one of the most intensively studied areas in weed science because of its direct consequences for crop yield and production costs. Although major advances have been made in identifying resistance mechanisms, the translation of these insights into technologies that can slow the evolution of resistance remains limited. This review synthesizes current knowledge of herbicide resistance with a strong emphasis on mechanism‐informed management. We provide a detailed analysis of target‐site resistance mutations across major herbicide modes of action, highlighting mutation‐ and species‐specific cross‐resistance patterns that enable rational herbicide selection beyond blanket mode‐of‐action exclusion. We also comprehensively address non‐target‐site resistance with attention to environmental modulation of resistance expression, and a first unified overview of all metabolic genes underlying herbicide resistance. Finally, we discuss a forward‐looking framework linking molecular diagnostics and genomics to field‐level resistance management and stewardship. Understanding the mechanisms of resistance can reveal potential weaknesses. © 2026 The Author(s). *Pest Management Science* published by John Wiley & Sons Ltd on behalf of Society of Chemical Industry.

## Graphical abstract

This review bridges the gap between understanding resistance mechanisms and applying them in weed management, highlighting how mechanistic insights reveal vulnerabilities that can guide more effective control strategies.

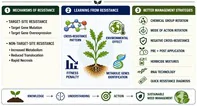

## 1 INTRODUCTION

Through evolution, weeds can overcome nearly every weed management strategy, whether it is mechanical, cultural, or chemical.^1^ Chemical (herbicide) resistance has repeatedly evolved, especially in systems with simplified chemical weed management (the repeated application of a few herbicide modes of action (MoAs) over several years) and where the weed population contains sufficiently high genetic variation. Furthermore, this problem is compounded by individual resistance traits stacking in the offspring of surviving individuals through pollen‐mediated gene flow so that resistance to several MoAs can be found in the same population or even the same individuals, often deemed ‘superweeds’. The development of new herbicide MoAs capable of controlling both single and complex herbicide resistance cases remains a major challenge for the industry; therefore, we can assume that there will not be a ‘silver‐bullet’ solution in the short term to herbicide resistance and that resistance will get worse before it gets better. In some situations, new herbicide MoAs may start being released to market soon; however, past experience says that it is likely that weeds will also evolve resistance to them if we do not steward their use carefully and implement strategies based on what we have learned from management mistakes in past years.

Currently, there are 113 reports of unique weed populations resistant to two or more herbicide MoAs,^2^ which highlights the future challenging scenario. The most feasible current strategy for preventing and controlling herbicide resistance is the mixture or rotation of herbicides with different MoAs.^3^ In the current situation, where we lack new chemistries and continue to have more stacked resistance genes, we have and will have to fight this battle with the available chemistries. The critical question is: how? One important strategy is to fundamentally understand resistance mechanisms and how they evolve so that we can identify possible weaknesses to exploit. Several analyses present ‘lessons’ or ‘what we have learned’ from herbicide resistance over the years,^4^, ^5^, ^6^, ^7^ but, to our knowledge, there are no publications directly considering the relationship between mechanisms of resistance and management strategies, and how one may directly influence the other. This review aims to fill this gap by presenting several cases in which detailed knowledge of the resistance mechanism can lead to improved weed management outcomes.

Herbicide resistance mechanisms are classified into two major categories, target and non‐target site resistance (TSR and NTSR, respectively) mechanisms. TSR mechanisms are alterations in herbicide target binding or overexpression of the gene encoding the target protein. NTSR mechanisms encompass all changes that alter the speed or amount of herbicide that reaches its target, including, but not limited to, mechanisms that lead to reduced absorption, reduced translocation due to vacuolar sequestration, reduced translocation due to rapid tissue necrosis, or metabolic resistance^8^ (Fig. 1). Among these resistance mechanisms, increased metabolism is a particular concern as the specific herbicides that are metabolized are relatively unpredictable, and a single mechanism can confer resistance to numerous chemicals spanning many MoA. For example, in *Lolium rigidum*, the up‐regulation of one single metabolic gene confers resistance to five herbicide MoAs.^9^ Common enzymes involved in metabolic resistance are cytochrome P450 monooxygenases (CYPs), glutathione‐S‐transferases (GST), UDP‐glycosyltransferases (UGT), aldo‐keto reductases (AKR), and ATP‐binding cassette transporters (ABC).^10^ Recently, a new NTSR enzyme family, β‐ketoacyl‐CoA synthase (KCS), has been characterized as playing a role in paraquat resistance by significantly promoting the endogenous polyamines metabolic pathway.^11^ A comprehensive understanding of TSR mechanisms requires at least a basic molecular biology laboratory infrastructure, while NTSR studies often require much more extensive biochemistry and bioinformatic infrastructure, including both the equipment and expertise. However, molecular and genomic studies in weeds have recently become much more tractable. Since the first relevant weed species genome was sequenced in 2017 (*Echinochloa crus‐galli*),^12^ several others have been made available,^13^ facilitating a more rapid discovery of herbicide resistance genes. The number of papers investigating complex resistance traits, such as metabolic resistance, has been increasing in the last few years. In addition, the knowledge of polyploid species genomes helps in sequencing the herbicide target sequences from all subgenomes, avoiding false‐negative results. With this ever‐growing catalogue of resistance mechanisms, the question becomes, how can we use this information for better weed management? This review compiles scientific results and our perspective on how the investigation of herbicide resistance mechanisms can be applied to manage weeds in the field or to develop technology to support it.

**Figure 1:**
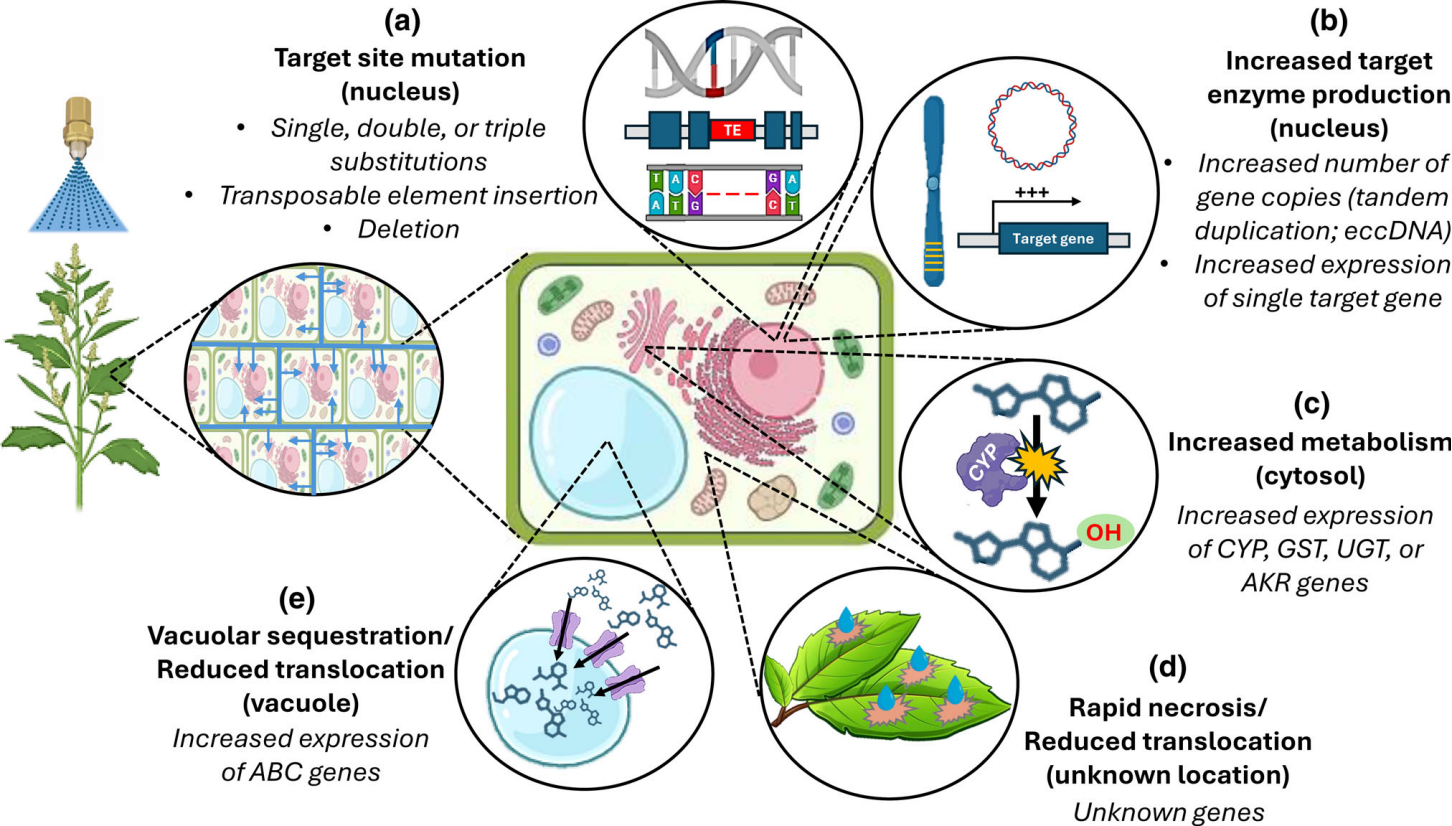
Comprehensive illustration of target‐site (a, b) and non‐target‐site (c–e) herbicide resistance mechanisms. Image created with BioRender.

## 2 DIVERSITY OF TARGET SITE MECHANISMS RESULTS IN VARIATION OF LEVEL AND CROSS‐RESISTANCE

TSR mechanisms are the best‐characterized in weeds, usually involving a single or multiple mutations (e.g., amino acid replacements, amino acid deletion, or changes in codon reading frame due to transposable element insertion) in the gene sequence that encodes the target enzyme, but could also involve increased expression of the target gene either as a consequence of alteration in *cis‐* or *trans*‐regulatory elements or from increased target gene copies.^8^ A single amino acid substitution can decrease the target enzyme binding affinity to either a single or multiple herbicides from the same MoA, disrupting the lock‐and‐key model of herbicide action. Typifying this sort of resistance are those herbicides that disrupt branch chain amino acid synthesis by inhibiting acetolactate synthase (ALS). The *ALS* gene has nine codons that can contain mutations conferring resistance to one or more Group 2 herbicides. These simple polymorphisms are also common resistance mechanisms for other herbicide MoAs, such as herbicides that target acetyl‐CoA carboxylase (ACCase) and 5‐enolpyruvylshikimate‐3‐phosphate synthase (EPSPS) proteins. While target site mutations have been reported for over 30 years, increased target expression was discovered about 15 years ago.^14^ Increased target gene expression results in increased herbicide target enzymes, necessitating a larger dose of herbicide to result in the same level of control, in a fairly linear correlation.^15^

Understanding patterns of cross‐resistance from TSR mechanisms can lead to a more rational rotation of herbicides in the field and help the management of the resistant biotypes. As TSR mechanisms were the first discovered resistance mechanisms, most of the herbicide resistance management strategies recommend rotating herbicides with different MoAs or using multiple herbicides simultaneously, and to discontinue the use of all compounds belonging to the MoA for which resistance has been identified. This is the best and the ideal recommendation, but it is difficult to implement due to the limited number of different herbicide MoAs that show crop selectivity and weed control efficacy at the same time. Furthermore, not all herbicides within the same MoA bind the target the same way, and sometimes rotating to another chemical class within the same MoA is still effective. In addition, some herbicides such as ALS, ACCase, EPSPS inhibitors, and auxin mimics have a favorable environmental profile, low cost, use at post‐emergence, and broad spectrum, which justifies utilization even considering resistance scenarios. Through years of research, we have accumulated hundreds of cases of TSR resistance in different weed species, to different herbicides, cropping systems, and locations, showing variability of resistance level and cross‐resistance. This already built knowledge, combined with the forthcoming field‐level diagnostics of predominant target‐site mutations enabled by high‐throughput genotyping, may support alternative active ingredient recommendations even within MoA for which resistance has been reported.^16^, ^17^

### 2.1 Target site mutations and their cross‐resistance patterns

#### 2.1.1 Acetolactate synthase (ALS) mutations

The MoA with the most active ingredients is the ALS inhibitors (HRAC Group 2), and they are also the most versatile in terms of crop selectivity range, such as corn, soybean, rice, and wheat, as well as for their pre‐ and post‐emergence efficacy. ALS inhibition prevents the production of branched‐chain amino acids leucine (Leu), isoleucine (Ile), and valine (Val).^18^ These herbicides are usually effective at relatively low rates (e.g., 5–30 g ha^−1^), which makes them cheap and relatively environmentally safe. The ALS‐inhibiting herbicides are classified into seven chemical groups: sulfonylureas (SU), imidazolinones (IMI), triazolopyrimidine type 1 and type 2 (TP), pyrimidinylbenzoate (PB), triazolinones (TZ), and sulfonanilides (SA). Their wide range of utility in several crops and the relative ease with which a single mutation provides resistance make this MoA the most resistance‐prone.^2^ It should be noted that TSR and NTSR mechanisms have been reported for ALS inhibitors, though TSR seems to be the dominant mechanism in most cases. The *ALS* gene has the most variability of amino acid substitution conferring resistance, involving up to nine positions (Ala122, Pro197, Ala205, Phe206, Asp376, Arg377, Trp574, Ser653, and Gly654), the most common being Trp574Leu (identified in 37 species) and Pro197Ser (identified in 27 species).^2^

The interesting factor about TSR in *ALS* is that each point mutation has a different pattern of cross‐resistance, according to the specific replaced amino acid and weed species. In general, the mutations Trp574Leu or Asp376Glu confer resistance to all ALS‐inhibiting herbicide chemical groups in several species evaluated, and at high resistance levels.^2^, ^19^, ^20^, ^21^ However, the Asp376Glu showed a different resistance pattern within the IMI group members in *Sorghum halepense* and *Amaranthus retroflexus*. The recommended label rate of imazethapyr showed high resistance level in both species, whereas the imazamox label rate was efficacious in controlling them.^22^ When molecular assays detect Trp574Leu mutation, it is not recommended to rotate to any other Group 2 herbicide, as they will most likely be ineffective. While Trp574Leu is the most common substitution, cases of Trp574Gly^23^ or Trp574Arg^24^ have been reported as conferring resistance to IMI, TP, and SU (PB, TZ, and SA were not tested), and Trp574Met^25^ conferring resistance to SU (the other chemical groups were not tested). Mutations in the position Pro197 are also very common, and can be substituted for several amino acids including threonine (Thr), histidine (His), arginine (Arg), Leu, glutamine (Gln), serine (Ser), alanine (Ala), Ile, asparagine (Asn), glutamate (Glu), tyrosine (Tyr), or phenylalanine (Phe) in several species with the most common being Pro197Ser, Pro197Thr, Pro197Leu, Pro197Ala, and Pro197His^2^ which results in different patterns of cross‐resistance associated with the amino acid replaced. The mutations Pro197Ser and Pro197Ala provide resistance to TP, SU, and PB chemical classes, but not for IMI in *Conyza canadensis*.^26^ The mutation Pro197Thr provides cross‐resistance between SU, TP, and TZ, but not for IMI and PB chemical groups in *Descurainia sophia*.^27^ Pro197Arg showed resistance to SU and IMI in *Conyza bonariensis* ^28^ but susceptibility to IMI in *Lolium rigidum*.^29^ Similarly, the amino acid Ala122 can be substituted by Thr, Val, Tyr, Ser, or Asn. The mutation Ala122Thr provides resistance to IMI, but not to TP, SU, and PB in *Echinochloa crus‐galli* ^30^ and *Amaranthus hybridus*.^31^, ^32^ If Ala122 is replaced by Asn instead of Thr, it confers resistance to IMI, TP, SU, and PB in *Echinochloa crus‐galli* ^33^; in *Raphanus raphanistrum*, the presence of Tyr at position 122 conferred resistance to IMI, TP, and SU.^34^ For the position Ser653, it can be substituted by Thr, Asn, or Ile. In *Echinochloa crus‐galli*, the Ser653Asn confers resistance only to IMI, and susceptibility to TP, SU, and PB,^30^ while in *Bromus tectorum* the same mutation resulted in IMI, TP, and TZ resistance, but susceptibility to SU,^35^ and in *Setaria viridis* resistance to IMI, TZ, and PB, but susceptibility to SU.^36^ The mutation Ser653Thr conferred resistance to IMI, but susceptibility to SU, PB, and TZ, while the Ser653Ile showed resistance to IMI, SU, and PB but susceptibility to TZ in *Setaria viridis*.^36^

Taken together, knowledge of the specific mutation and the specific weed species can help provide herbicide rotation options for growers, even within the same MoA between chemical groups. However, it takes a deep understanding of these species × mutation × herbicide interactions to make rotation recommendations with confidence. Additionally, researchers would need to have the ability to identify specific amino acids from dozens of individuals to assess an entire field or weed population, as many mutations can occur simultaneously in a population. A better understanding of species × mutation × herbicide interactions and the epidemiology of the resistance spread could help design efficient management programs and preserve this MoA, or at least how to most effectively incorporate it in an integrated weed management strategy. For example, many *Echinochloa crus‐galli* imazethapyr (IMI) resistant populations from Brazil were controlled when sprayed with penoxsulam (TP), despite both imazethapyr and penoxsulam being Group 2 herbicides.^30^, ^37^ The favorable environmental profile and low cost justify the continued utilization of ALS inhibitors. In addition, while challenged by resistance in some species, the ALS inhibitors remain valuable tools for managing other susceptible ones^38^; however, their rational utilization needs to be optimized.

#### 2.1.2 Acetyl‐CoA carboxylase (ACCase) mutations

ACCase‐inhibitors (HRAC Group 1) are the main strategy to control grasses, especially in a scenario where resistance to glyphosate and ALS‐inhibitors is widely spread. The inhibition of ACCase enzyme inhibits fatty acid biosynthesis.^39^ This MoA has four chemical groups: aryloxyphenoxypropionate (FOP), cyclohexanedione (DIM), phenylpyrazolin (DEN), and the most recent carbocyclic aryl‐dione, represented only by metproxybicyclone.^40^ The importance of this MoA is set to increase with the recent development of crops resistant to FOP chemical group, including Enlist® Corn (Corteva Agriscience), Provisia® Rice (BASF), Max‐Ace® Rice (RiceTec), and CoAXium® Wheat (Limagrain). Several of the most important weedy grass species have populations resistant to ACCase‐inhibitor herbicides, including *Lolium* sp.,^41^ *Echinochloa* sp.,^42^ *Alopecurus myosuroides*,^43^ *Poa annua*,^44^ and *Eleusine indica*.^45^

Mutations in seven positions in *ACCase* gene are known to confer resistance to ACCase inhibitors, including Ile1781, Trp1999, Trp2027, Ile2041, Asp2078, Cys2088, and Gly2096, all located in the carboxyl‐transferase domain of the ACCase protein. In general, the mutation Ile1781Leu and Ile1781Thr confer resistance to all three chemical groups FOP, DIM, and DEN^46^, ^47^, ^48^, ^49^; although, Ile1781Leu resistance to DIMs can be variable, with very low resistance to clethodim in *Digitaria ciliaris* and *Alopecurus myosuroides* ^46^, ^50^ but higher in *Leptochloa chinensis*.^51^ The mutation Trp1999Cys confers resistance to FOP and very low to DEN, but remains susceptible to DIMs.^51^, ^52^ This mutation also provides variable resistance to different FOP chemicals, with resistance to fenoxaprop but susceptibility to haloxyfop and clodinafop.^52^, ^53^ Other less common amino acid replacements have also been identified in this position; the Trp1999Ser confers resistance to FOPs, DIMs, and DEN in *Lolium multiflorum*,^54^ while the Trp1999Leu in *Alopecurus japonicus* confers resistance to fenoxaprop but is still susceptible to haloxyfop, clodinafop, and DIMs.^53^ The mutation Trp2027Cys confers resistance to FOPs and DENs but remains susceptible to DIMs in *D. ciliaris* and *Alopecurus myosuroides*,^50^, ^55^ whereas in *Leptochloa chinensis* and *Avena sterilis* the same mutation confers resistance to FOPs, DIMs, and DEN.^51^, ^52^ The mutation Ile2041Asn confers resistance to FOPs, DIMs (sethoxydim, but susceptibility to clethodim), and DEN in *Digitaria ciliaris*.^55^ The mutation Ile2041Thr in *Bromus tectorum* conferred resistance to FOPs, but not to DIMs (sethoxydim and clethodim).^56^ The mutation Asp2078Gly confers resistance to FOPs, DIMs, and DENs in several species, like *Digitaria insularis*, *Polypogon fugax*, and *Lolium* sp.^48^, ^57^, ^58^ The mutation Asp2078Glu confers resistance to FOPs and DEN in *Echinochloa crus‐galli*, but DIMs were not tested.^59^ The mutation Cys2088Arg confers resistance to FOPs, DIMs, and DEN in *Lolium* sp.^48^, ^60^ The Gly2096Ala confers resistance to FOPs and DIMs (sethoxydim, but susceptibility to clethodim) in *Bromus tectorum*, but DEN was not tested.^56^ The Gly2096Ser confers resistance to FOPs, DIMs (including clethodim), and DEN in *Phalaris paradoxa*.^61^ The new ACCase inhibitor metproxybicyclone, from the novel carbocyclic aryl‐dione chemical group, was effective in controlling *Lolium multiflorum* carrying the Ile1781Leu, Trp1999Ser, Trp2027Cys, Ile2041Asn, Asp2078Gly, and Cys2088Arg mutations, and *Eleusine indica* carrying Trp2027Cys, Asp2078Gly, and Gly2096Ala in the *ACCase* gene.^40^

Similar to ALS‐inhibiting chemistries, these results show the variability of cross‐resistance patterns when comparing the same mutation in different species. However, *ACCase* mutations also show different responses to different active ingredients within the same chemical group, for example, sethoxydim and clethodim. Clethodim seems to be effective in controlling several mutations, even in situations where sethoxydim fails; however, it is notorious that all mutations confer resistance to FOP herbicides, especially to fenoxaprop. Knowledge of these mutations and their presence and resistance profile provide an opportunity for herbicide resistance control, that is, it may still be possible to use ACCase inhibitors if there are resistance mutations.

#### 2.1.3 Protoporphyrinogen oxidase (PPO) mutations

Protoporphyrinogen oxidase (PPO)‐inhibitor herbicides (HRAC Group 14) are mostly utilized to control broadleaf weeds, especially when troublesome weeds become resistant to glyphosate and ALS‐inhibitors, such as *Amaranthus* sp. The inhibition of PPO enzyme results in reactive oxygen species (ROS) production, leading to loss of membrane function and integrity.^62^ The PPO‐inhibitors are classified into five chemical groups: N‐phenyl‐imides (PI), diphenyl ethers (DE), N‐phenyl‐oxadiazolones (PO), N‐phenyl‐triazolinones (PT), and phenylpyrazoles (PP). Only a few mutations endow resistance to PPO‐inhibitors, and most of them are found in *PPX2* gene, which encodes both plastidic and mitochondrial PPO enzyme isoforms. One of the most common TSR mechanisms is a 3‐bp deletion (rather than a substitution) that has been identified in *PPX2* from *Amaranthus tuberculatus*, which deletes a glycine at position 210 (ΔGly210).^63^ Furthermore, ΔGly210 confers resistance to DE (fomesafen), PT (carfentrazone), and PI (flumioxazin and saflufenacil) chemical groups in *Amaranthus palmeri*.^64^ A PPX2 enzymatic assay from *Amaranthus palmeri* showed that the ΔGly210 makes the enzyme insensitive to all PPO‐inhibiting herbicide chemical groups tested.^65^

Other resistance mutations in *PPX2* are regular amino acid substitutions. The mutation Arg128Leu (also referred to as Arg98Leu) confers resistance to DE (lactofen), PI (saflufenacil), and pyraflufen‐ethyl (without a chemical group), but susceptibility to flumioxazin (PI) in *Euphorbia heterophylla*,^66^ and resistance to DE (acifluorfen, fomesafen, lactofen, and oxyfluorfen), PI (flumiclorac and flumioxazin), PT (carfentrazone), and pyraflufen in *Ambrosia artemisiifolia*.^67^ However, an enzymatic assay of PPX2 from *Amaranthus palmeri* showed that the mutation Arg128Leu confers resistance to DE (acifluorfen, fomesafen, lactofen, and oxyfluorfen), PI (saflufenacil, butafenacil, and flumioxazin), PT (sulfentrazone and carfentrazone), but no resistance to PO (oxadiazon), pyraflufen, and pyraclonil (both without a defined chemical group).^65^ In addition, other amino acid replacements were found in the same position, such as Arg128Met, Arg128Gly, and Arg128Ile mutations in *Amaranthus* spp., which were associated with DE (fomesafen) resistance.^68^ The mutation Gly399Ala in *Amaranthus palmeri* confers cross‐resistance to all PPO‐inhibiting herbicide chemical groups according to an enzymatic assay^65^; however, *in planta*, *Amaranthus palmeri* study showed that Gly399Ala confers resistance to DE (fomesafen and lactofen) and PT (carfentrazone), and PI (flumioxazin), but not to DE (acifluorfen) and PI (saflufenacil).^65^ A Val361Ala mutation in *PPX2* from *Amaranthus palmeri* confers resistance to DE (fomesafen).^69^

The only mutation found in the *PPX1* gene was identified in *Eleusine indica*. The mutation is Ala212Thr, which confers resistance only to PO (oxadiazon) but not to PT (sulfentrazone), PI (flumioxazin, saflufenacil, and trifludimoxazin), and DE (lactofen).^70^ The PPX1 enzyme is thought to be located solely in the chloroplast, while PPX2 is dual‐targeted to both the chloroplast and mitochondria. The occurrence of the mutations in the *PPX2* gene, which encodes both plastidic and mitochondrial PPO isoforms, implies that a target‐site mutation in *PPX2* leads to resistance in both organelles.^71^ The mutations reported in *PPX2* can protect PPO activity not only in the mitochondria, but also in the chloroplast, explaining why most reported mutations were only associated with *PPX2*.^70^

In summary, all mutations in *PPX2* confer resistance mainly to the DE chemical group, which must be avoided for the management of the resistant populations. Active ingredients from different, or within the same, chemical groups can have differential sensitivity to amino acid changes in the enzyme. For example, saflufenacil is more effective than fomesafen in resistant biotypes of *Amaranthus palmeri* because fomesafen has a weaker binding affinity, a smaller interaction volume, and volume overlap with the substrate protoporphyrinogen IX than saflufenacil.^72^ The new PPO‐inhibitor epyrifenacil belongs to the PI chemical group, and is new to the market, showing a broad spectrum ability to control grasses as well as the potential for controlling populations with ΔGly210, Arg128Leu, or Gly399Ala.^73^ In the same way, trifludimoxazin, which also belongs to the PI chemical group, showed similar control when comparing susceptible and resistant *Amaranthus* carrying ΔGly210, Arg128Leu, or Val361Ala.^74^

#### 2.1.4 Indole‐3‐acetic acid (IAA) mutations

The auxinic herbicides (HRAC Group 4) do not have a well‐defined target enzyme, as several receptors are involved in auxin signaling and recognition. In general, these herbicides mimic native auxin hormones, particularly indole‐3‐acetic acid (IAA), which leads to misregulation of hormone homeostasis and plant death from incorrect growth and environmental response. The current model of auxinic herbicide activity involves several classes of proteins, but the target site mutations identified to date are mutations, deletions, and insertions in Aux/IAA transcriptional repressors.^8^ The reduced auxinic herbicide binding to the Aux/IAA repressor(s) prevents the activation cascade of auxin‐responsive genes, and consequently, no herbicidal symptomology.^75^ Auxinic herbicides are classified into six chemical groups: phenoxycarboxylates (i.e., 2,4‐D), 6‐chloropicolinates (i.e., picloram), 6‐arylpicolinates (i.e., florpyrauxifen), pyridyloxycarboxylates (i.e., fluroxypyr), benzoates (i.e., dicamba), and quinolinecarboxylates (i.e., quinclorac). Discoveries of TSR to this MoA are relatively recent, despite it being the oldest herbicide class; however, TSR reports are increasing with the advancement of weed genomic resource availability, a greater understanding of auxinic herbicide sites of action, and an increased use of auxinic herbicides due to Enlist® (2,4‐D resistant) and Xtend® (dicamba resistant) crops.

The first auxinic target site mutation identified was in *Bassia scoparia* with a Gly127Asn mutation in the domain II degron of *IAA16*, reportedly providing resistance to dicamba, 2,4‐D, fluroxypyr, picloram, and quinclorac.^76^ In *Chenopodium album*, another substitution in *IAA16*, Gly127Asp, also confers resistance to dicamba.^77^ Again in *IAA16* of *Bassia scoparia*, an insertion of a retrotransposon close to the beginning of the second exon led to disruption of normal splicing, resulting in the addition of one amino acid and the replacement of five more in the final protein product, including a Gly127Thr in the degron, causing resistance to dicamba and 2,4‐D.^78^ In *Sisymbrium orientale*, a 27‐nucleotide deletion removing nine amino acids in the degron tail of the auxin co‐receptor *IAA2*, conferred resistance to dicamba and 2,4‐D.^79^ Also in *Sisymbrium orientale*, the Leu175Pro mutation located in a region called Phox and Bem1 (PB1) in *IAA34* conferred resistance to 2,4‐D and MCPA.^80^ Other auxinic herbicides, such as dicamba, fluroxypyr, triclopyr, and picloram, were also tested and showed increased median lethal dose (LD_50_) when compared to the wild‐type. However, the fluroxypyr resistance was not confirmed when evaluated in transformed Arabidopsis.^80^ In *Sonchus oleraceus*, a deletion of six amino acids in the region flanking the degron of *IAA20*, three aspartic acid (Asp) upstream and three glycines (Gly) downstream, confers resistance to dicamba, 2,4‐D, fluroxypyr, and clopyralid (6‐chloropicolinates).^81^ Mutations in *IAA* decrease the auxin binding affinity of the degron domain mutants with the TIR/AFB F‐box proteins. Even if not all auxinic herbicides were evaluated in most studies, the mutations in *IAA* probably confer cross‐resistance to other auxinic herbicides due to their important role in auxinic perception. In this scenario, rotating growing seasons with 2,4‐D‐ or dicamba‐resistant crops will not be effective in controlling target site dicamba or 2,4‐D‐resistant weeds. Considering the cross‐resistance, rotation within the Group 4 MoA should be avoided until more data is collected about these relatively new resistance cases.

#### 2.1.5 The psbA mutations

Photosystem II (PSII)‐inhibitors (HRAC Groups 5 and 6) are pre‐ and post‐emergent herbicides selective to several crops, but heavily used in corn. The target of these herbicides is the D1 protein, located in the PSII complex, competing with plastoquinone and consequently blocking electron transfer from PSII to plastoquinone. After inhibition, high‐energy electrons accumulate in the thylakoid, leading to chloroplast damage and plant death.^82^ PSII‐inhibitors are divided into two major categories: ‘D1 non‐histidine 215 binders’ (HRAC Group 5) and ‘D1 histidine 215 binders’ (HRAC Group 6). D1 non‐histidine 215 binders are split into six chemical groups: triazines, ureas, uracils, phenylcarbamates, amides, and triazinones, whereas the D1 histidine 215 binders contain the nitrile chemical group. Additionally, both categories have active ingredients without official chemical family names. The D1 protein is encoded by the *psbA* gene, which is in the chloroplastic genome, therefore maternally inherited.^83^ Known mutations in the *psbA* gene that confer herbicide resistance are Leu218Val, Val219Ile, Ala251Val, Phe255Ile, Ser264Gly, Asn266Thr, and Phe274Val. The most common mutation is Ser264Gly, which confers resistance to triazines (atrazine, simazine, terbuthylazine), triazinones (metribuzin, metamitron), and amicarbazone (a D1 non‐histidine 215 binder without a chemical family name) in several species, such as *Chenopodium album*,^84^ *R. raphanistrum*,^85^ *Poa annua*,^86^ and *Amaranthus retroflexus*.^87^ However, plants carrying the Ser264Gly are still susceptible to ureas (linuron, diuron), and nitriles (bromoxynil), as verified in *Chenopodium album* ^88^ and *R*. *raphanistrum*.^89^ The Leu218Val confers resistance to triazinones (metribuzin, metamitron) but remains susceptible to triazines (terbuthylazine), while Ala251Val confers resistance to both triazinones and triazines in *Chenopodium album*.^84^ The Val219Ile confers resistance to ureas (linuron, diuron, monolinuron), triazinones (metribuzin), amides (propanil), and nitriles (bromoxynil) in *Amaranthus powellii*,^90^ *Poa annua*,^91^ and *Cyperus difformis* ^92^; however, this mutation is still susceptible to bentazon (a D1 histidine 215 binder without chemical family name) and triazines (atrazine).^90^, ^92^ The Phe255Ile confers resistance to triazinones (hexazinone, metribuzin), but remains susceptible to triazines (atrazine), ureas (diuron), and uracils (terbacil) in *Capsella bursa‐pastoris* ^93^ and *Festuca filiformis*.^94^ The mutation Asn266Thr confers resistance to uracils (terbacil), triazinones (metribuzin, hexazinone), and nitriles (bromoxynil), but remains susceptible to triazines (atrazine, simazine) and ureas (diuron) in *Senecio vulgaris*.^95^ The mutation Phe274Val confers resistance to triazines (atrazine), triazinones (metribuzin), and ureas (diuron), but remains susceptible to nitriles (bromoxynil) in *R. raphanistrum*.^89^ These results show that PSII TSR is manageable with rotating chemical families, including for the most prevalent mutation Ser264Gly.

#### 2.1.6 α‐Tubulin mutations

Microtubule assembly inhibitors (HRAC Group 3) control predominantly grass weeds and are essentially pre‐emergent herbicides divided into three chemical families: dinitroanilines, pyridines, and phosphoroamidates. These herbicides bind to the α‐tubulin, inhibiting the microtubule polymerization during the mitotic cycle and ultimately inhibiting cell division, especially in root tips.^96^ TSR mechanisms are primarily due to amino acid changes in the *α*‐*tubulin* gene at different positions. The mutations Leu136Phe, Thr239Ile, Val202Phe, Arg243Met/Lys, and Met268Thr confer resistance to dinitroaniline herbicides in several grass species, such as *Lolium rigidum*, *Poa annua*, *Eleusine indica*, and *Setaria viridis*.^97^, ^98^, ^99^, ^100^, ^101^, ^102^ The dinitroaniline chemical group should be avoided when species carrying any of the known target‐site mutations in the *α‐tubulin* gene are detected. The mutations Leu136Phe and Thr239Ile also confer resistance to chlorthal‐dimethyl, whereas Thr239Ile also confers resistance to pyridines.^100^, ^101^, ^102^ The other mutations were not tested for different chemical groups outside of dinitroanilines. Unlike other herbicides, resistance to microtubule assembly inhibitors due to target site mutation is recessive, which means that heterozygous plants are still susceptible,^103^ providing a small management opportunity, especially for cross‐pollinating species such as *Lolium rigidum*.

#### 2.1.7 5‐Enolpyruvylshikimate‐3‐phosphate synthase (EPSPS) mutations

Glyphosate (HRAC Group 9), which inhibits the EPSPS enzyme, is the most used herbicide, especially in the United States, due to the high adoption of Roundup Ready crops. This heavy adoption has led to several high‐profile cases of herbicide resistance.^104^ EPSPS inhibition prevents the biosynthesis of the aromatic amino acids: Phe, Tyr, and tryptophan (Trp).^105^ Weeds have evolved TSR via single Pro106Ser,^106^ double Thr102Ile + Pro106Ser^107^ or Thr102Ile + Pro106Thr,^108^ and triple Thr102Ile + Ala103Val + Pro106Ser^109^ mutations in the gene *EPSPS*. However, glyphosate is the only active ingredient in this MoA, and consequently, there is no cross‐resistance pattern to be evaluated. The mutation Pro106Ser is the most common and is known to confer low resistance level in most of the cases^106^; then, increasing glyphosate rates associated with crop varieties with superior competitive ability has potential benefit in suppressing populations with this mutation; however, the double and triple mutations confer high levels of glyphosate resistance, and increasing the glyphosate rate is not effective.

#### 2.1.8 Glutamine synthetase (GS) mutations

Another important herbicide that is the single member of its MoA is glufosinate‐ammonium (HRAC Group 10), which inhibits the glutamine synthetase (GS) enzyme, preventing the synthesis of Gln from Glu and ammonia.^110^ Most importantly, inhibition of GS results in an accumulation of ammonia, which is highly damaging even at low amounts. The number of resistance cases to glufosinate‐ammonium is low at this time, with only a few target site mutations reported that confer resistance, such as Ser59Gly in *GS1* ^111^ and Asn171Asp in *GS2*.^112^ There are no management implications associated with the mechanism of resistance to this herbicide, because it is the single member of this MoA.

#### 2.1.9 Target site resistance level is affected by zygosity or species ploidy level

Species ploidy level can impact the resistance level of a target site mutation. The mutation Ser653Asn confers low IMI resistance level in hexaploid *Echinochloa crus‐galli* ^30^ but high levels in diploid species, such as *Bromus tectorum* ^35^ and weedy rice.^113^ In hexaploid *Avena fatua*, the *ACCase* mutations Ile1781Leu, Asp2078Gly, and Cys2088Arg located in only one subgenome, conferred a lower resistance level to diclofop and sethoxydim than in the diploid species *Lolium rigidum*.^48^, ^114^ In hexaploid *Echinochloa colona*, the *EPSPS* mutation Pro106Ser conferred a low resistance level,^115^, ^116^ whereas in diploid species such as *Lolium rigidum* and *Conyza canadensis*, the *EPSPS* mutation Pro106Ser conferred a higher resistance level.^117^, ^118^ This is thought to be because there is a ‘dilution effect,’ where not all the enzymes are mutated (in a hexaploid as few as one‐sixth of the protein pool will have the resistance mutation in the case of a single subgenome heterozygote). A given target site mutation conferring resistance in diploid species may not be sufficient in polyploid species to be selected in the field, buffering the effect of resistance mutations.^114^ An additional factor influencing resistance in polyploids is the presence of a target site mutation in a subgenome that is more or less expressed.^30^ In hexaploid *Echinochloa crus‐galli*, the *ALS* mutation Trp574Leu in subgenome C conferred 20‐fold more resistance to imazethapyr and penoxsulam when compared to subgenome A, because the *ALS* from subgenome C was four times more expressed than *ALS* from subgenome A.^30^ The same trend was observed in hexaploid wheat (*Triticum aestivum*), where the ACCase mutation Ala2004Val in subgenome A conferred higher resistance to quizalofop‐*p*‐ethyl than in subgenome B.^119^ However, this is not always the case, because different species have different genetic regulation mechanisms. The presence of Pro197Ser in *ALS* in different subgenomes of tetraploid *Sonchus oleraceus* did not affect the resistance level to ALS inhibitors.^120^

The occurrence of target site mutation in either homozygosity or heterozygosity state in diploid species can also influence the resistance level due to an incomplete dominance. In *Eleusine indica* resistant to glyphosate with Pro106Ser mutation in the *EPSPS* gene, the heterozygous genotype showed intermediate median effective dose (ED_50_) between the homozygous susceptible Pro106 and homozygous resistant Ser106.^106^ It is important to highlight that the heterozygous individuals were still resistant to glyphosate. In case of recessive target site mutation characteristics, such as the case of α‐tubulin mutations, the heterozygous individuals will be susceptible to the microtubule assembly inhibitors,^103^ and the resistance evolution will not be perceived in the field until there is predominance of homozygous individuals. Researchers should pay attention to double or triple peaks when analyzing the DNA sequencing chromatogram, because the ploidy level or heterozygosity may mask the presence of target site mutation analysis.

#### 2.1.10 Implications of target site mutations on management

Several weed resistance reports generalize resistance to one active ingredient across all other active ingredients within the same MoA. Similarly, farmers and consultants may generalize resistance when control failure is observed with one active ingredient, without testing other active ingredients within the same MoA. The studies described earlier indicate that this assumption is not always valid and oversimplifies cross‐resistance patterns (Table 1). When looking across these various herbicide MoAs, it is clear the importance of evaluating the specific mutation, the specific weed species, and the composition of multiple chemicals within a MoA to best use TSR mechanism information to form resistance management plans. For example, the Ser653Asn confers different cross‐resistance patterns in *Echinochloa crus‐galli* and *Bromus tectorum*, which, in turn, are different than in *Setaria viridis*.^30^, ^35^, ^36^ Different target site mutations can be managed with different chemical groups within the same MoA. While the Trp574Leu confers resistance across all ALS inhibitors in *Echinochloa crus‐galli*, the mutations Ala122Thr, Ala205Asn, and Ser653Asn still demonstrate susceptibility to penoxsulam (TP) and bispyribac‐sodium (PB), which are selective to rice.^30^ Even more specific is the variable efficacy of active ingredients within the same MoA in controlling target site mutations, such as clethodim × sethodydim, fenoxaprop × haloxyfop, and imazethapyr × imazamox (Table 1). Different species have different innate metabolic systems and physiology that influence the amount of herbicide reaching its target. It is important to highlight that more than one target site mutation is often stacked in the same plant, which increases resistance level, changes the cross‐resistance pattern, and complicates the resistance pattern of TSR mechanisms.^37^, ^64^, ^114^

**Table 1:** Examples of target‐site mutations that confer variable cross‐resistance patterns to herbicides within the same mode of action

Gene	Gene position	Mutation	Species	Resistance	Susceptibility	Reference
ALS inhibitors						
*ALS*	Ala122	Thr	*Echinochloa crus‐galli*, *Amaranthus hybridus*	IMI	SU, TP, PB	^30^, ^31^, ^32^
		Asn	*Echinochloa crus‐galli*	SU, IMI, TP, PB	None tested	^33^
		Tyr	*Raphanus raphanistrum*	SU, IMI, TP	None tested	^34^
	Asp376	Glu	*Galium aparine*	SU, IMI, TP, PB, TZ	None tested	^21^
			*Sorghum halepense*, *Amaranthus retroflexus*	IMI (imazethapyr)	IMI (imazamox)	^22^
	Pro197	Ala	*Conyza canadensis*	SU, TP, PB	IMI	^26^
		Ser	*Conyza canadensis*	SU, TP, PB	IMI	^26^
		Thr	*Descurainia sophia*	SU, TP, TZ	IMI, PB	^27^
		Arg	*Conyza bonariensis*	SU, IMI, TP, PB, TZ	None tested	^28^
			*Lolium rigidum*	SU	IMI	^29^
	Trp574	Leu	*Sinapis arvensis*, *Amaranthus palmeri*	SU, IMI, TP, PB, TZ	None tested	^19^, ^20^
	Ser653	Asn	*Echinochloa crus‐galli*	IMI	SU, TP, PB	^30^
			*Bromus tectorum*	IMI, TP, TZ	SU	^35^
			*Setaria viridis*	IMI, PB, TZ	SU	^36^
		Thr	*Setaria viridis*	IMI	SU, PB, TZ	^36^
		Ile	*Setaria viridis*	SU, IMI, PB	TZ	^36^
ACCase inhibitors						
*ACCase*	Ile1781	Leu	*Digitaria ciliaris*, *Avena fatua*, *Lolium rigidum*, *Alopecurus myosuroides*, *Leptochloa chinensis*	FOP, DIM, DEN	None tested	^46^, ^47^, ^48^, ^49^, ^50^, ^51^, ^55^
		Thr	*Alopecurus myosuroides*	FOP, DIM, DEN	None tested	^49^
	Trp1999	Cys	*Leptochloa chinensis*	FOP, DEN	DIM‐Clethodim	^51^
			*Avena sterilis*	FOP (fenoxaprop)	FOP (haloxyfop), DIM (sethoxydim)	^52^
			*Alopecurus japonicus*	FOP (fenoxaprop), DEN	FOP (haloxyfop, clodinafop), DIM	^53^
		Ser	*Lolium multiflorum*	FOP, DIM, DEN	None tested	^54^
		Leu	*Alopecurus japonicus*	FOP (fenoxaprop)	FOP (haloxyfop, clodinafop), DIM	^53^
	Trp2027	Cys	*Alopecurus myosuroides*, *Digitaria ciliaris*	FOP, DEN	DIM	^50^, ^55^
			*Leptochloa chinensis*, *Avena sterilis*	FOP, DIM, DEN	None tested	^51^, ^52^
	Ile2041	Asn	*Digitaria ciliaris*	FOP, DIM (sethoxydim), DEN	DIM (clethodim)	^55^
		Thr	*Bromus tectorum*	FOP, DEN	DIM	^56^
	Asp2078	Gly	*Digitaria insularis*, *Polypogon fugax*, and *Lolium* sp.	FOP, DIM, DEN	None tested	^48^, ^57^, ^58^
		Glu	*Echinochloa crus‐galli*	FOP, DEN	None tested (DIM not tested)	^59^
	Cys2088	Arg	*Lolium* sp.	FOP, DIM, DEN	None tested	^48^, ^60^
	Gly2096	Ala	*Bromus tectorum*	FOP, DIM (sethoxydim)	DIM (clethodim)	^56^
		Ser	*Phalaris paradoxa*	FOP, DIM, DEN	None tested	^61^
PPO inhibitors						
*PPX2*	Gly210	ΔGly	*Amaranthus palmeri*	DE, PI, PT	PI (trifludimoxazin)	^64^, ^74^
	Arg128	Leu	*Euphorbia heterophylla*	DE, PI, Pyraflufen	PI (flumioxazin)	^66^
			*Ambrosia artemisiifolia*	DE, PI (including flumioxazin), PT, Pyraflufen	None tested	^67^
	Gly399	Ala	*Amaranthus palmeri*	DE (fomesafen, lactofen), PI (flumioxazin), PT	DE (acifluorfen), PI (saflufenacil)	^65^
	Val361	Ala	*Amaranthus palmeri*	DE	PI (trifludimoxazin)	^69^, ^74^
*PPX1*	Ala212	Thr	*Eleusine indica*	PO	DE, PT, PI	^70^
PSII inhibitors						
*psbA*	Ser264	Gly	*Chenopodium album*, *Raphanus raphanistrum*, *Poa annua*, *Amaranthus retroflexus*	Triazines, Triazinones, Amicarbazone	Ureas, Nitriles	^84^, ^85^, ^86^, ^87^, ^88^, ^89^
	Leu218	Val	*Chenopodium album*	Triazinones	Triazines	^84^
	Ala251	Val	*Chenopodium album*	Triazinones, Triazines	None tested	^84^
	Val219	Ile	*Amaranthus powellii*, *Cyperus difformis*	Ureas, Triazinones, Amides, Nitriles	Bentazon, Triazines	^90^, ^92^
	Phe255	Ile	*Capsella bursa‐pastoris*, *Festuca filiformis*	Triazinones	Triazines, Ureas, Uracils	^93^, ^94^
	Asn266	Thr	*Senecio vulgaris*	Uracils, Triazinones, Nitriles	Triazines, Ureas	^95^
	Phe274	Val	*Raphanus raphanistrum*	Triazines, Triazinones, Ureas	Nitriles	^89^

*Note*: DE, diphenyl ethers; DEN, phenylpyrazolin; DIM, cyclohexanedione; FOP, aryloxyphenoxypropionate; IMI, imidazolinones; PB, pyrimidinylbenzoate; PI, N‐phenyl‐imides; PO, N‐phenyl‐oxadiazolones; PP, phenylpyrazoles; PT, N‐phenyl‐triazolinones; SA, sulfonanilides; SU, sulfonylureas; TP, triazolopyrimidine type 1 and type 2; TZ, triazolinones; ALS, acetolactate synthase; ACCase, acetyl‐CoA carboxylase; PPO, protoporphyrinogen oxidase; PSII, photosystem II.

Currently, several challenges are facing sustainable chemical management: (1) Multiple resistance has been identified in several weed species and continues to increase, (2) crops resistant to several MoAs are being developed, despite already existing weed resistance to them, and (3) the lack of new major herbicide MoAs. These challenges underscore and reinforce our need for a better understanding of TSR mutations in terms of cross‐resistance patterns in multiple weed species. The cross‐resistance pattern elucidation can be used to directly recommend herbicide rotation programs that minimize herbicide resistance evolution and prolong the market life of cheap, environmentally safe, and efficient MoAs, benefiting both growers and industry.

We are in a transition to a future with accessible molecular diagnosis of resistance for farmers, which could greatly help decision‐making.^16^, ^121^ For that, it is necessary to provide complete information when reporting TSR in the literature, for example, evaluating several herbicides from as many chemical groups as possible and characterizing whether the mutation confers resistance to all or only some active ingredients. This information can be used by agrochemical companies to search for or design molecules of already existing MoAs that are able to overcome common mutations already reported. For example, the herbicides epyrifenacil and metproxybicyclone, new PPO and ACCase inhibitors, respectively, are reported as efficient in controlling some target site resistant populations.^40^, ^73^

### 2.2 Target site mutations reflected in fitness penalty

Target site mutations modify the shape and function of an enzyme, which has a more‐or‐less optimized shape for its natural role in the plant; therefore, mutations may alter enzyme efficiency, resulting in a fitness penalty in the absence of herbicide application and compared to the susceptible biotype. The selection pressure from herbicides is so great that even relatively high fitness penalties can be tolerated if it means surviving chemical control.^122^ Fitness penalties have been studied for several herbicide resistance mechanisms, but only a few of them clearly show a reduction in plant vegetative and reproductive parameters. For example, the presence of double mutation TIPS (Thr102Ile + Pro106Ser) in the *EPSPS* gene confers high glyphosate resistance, but also drastic growth reduction. In *Eleusine indica* homozygous for TIPS, the plants showed less biomass accumulation and less seed production when compared to a biotype heterozygous for TIPS mutation, one with only Pro106Ser mutation, and a susceptible one.^123^ When competing with rice, the homozygous TIPS‐resistant biotype showed even more drastic penalties, with reductions in biomass accumulation and seed production decreasing by 90% and 85%, respectively, when compared to a resistant population with only the Pro106Ser mutation.^123^ This was confirmed in another population of TIPS *Eleusine indica* with 47% less biomass and 66% fewer seeds when compared to the resistant with only the Pro106Ser mutation without crop competition, and 95% less biomass and 96% fewer seeds when competing with soybean.^124^ These penalties are explained by the less efficient kinetic properties of EPSPS enzyme when the mutation is present.^107^

The fitness penalty examples are not specific to one MoA. The mutation Arg243Met in α‐tubulin, which confers resistance to dinitroanilines microtubule inhibitors (Group 3), also confers a high fitness penalty, with a severe reduction in *Lolium rigidum* growth, which is almost lethal when in competition with crops.^125^ This pronounced disadvantage means that this mutation is rare and may not have a high impact as a problem in the field, keeping homozygous resistant plants at a very low level. Even when heterozygous, the fitness penalty is present, especially at high temperatures (26 °C), but much less evident than when in homozygosity.^125^ The ACCase mutation Asp2078Gly has been shown to confer a fitness penalty to the resistant plants, reducing seed production and aboveground biomass in *Beckmannia syzigachne*, *Lolium rigidum*, and *Alopecurus myosuroides*.^126^, ^127^, ^128^ The ACCase enzyme kinetics, such as *V*_max_, are reduced when carrying the mutation Asp2078Gly compared to the wild‐type and Ile178Leu mutation.^127^ The mutation Ser264Gly in *psbA* gene, which encodes the D1 protein, confers resistance to the PSII‐inhibitor atrazine (Group 5) and is known to confer a severe fitness penalty. *Chenopodium album* with this mutation had reduced growth parameters, such as height and biomass, with and without wheat competition.^129^ *Brachypodium hybridum* with Ser264Gly also showed reduced growth parameters.^130^ The mutation Ser264Gly in the D1 protein directly impacts PSII function, leading to increased photoinhibitory damage, higher turnover rates of the D1 protein, and reduced PSII electron transfer efficiency.^131^, ^132^ Mutations in *IAA* genes that confer resistance to auxinic herbicides have shown variable fitness penalties in resistant plants. *Sisymbrium orientale* with a 27 bp deletion in *IAA2* did not confer any fitness penalty; the biomass accumulation and seed production were the same between resistant and susceptible.^133^ In *Bassia scoparia* carrying the Gly127Asn mutation in *IAA16*, a significant vegetative growth defect was observed, leading to 80–90% less biomass and seed production^134^; whereas a transposable element insertion in *IAA16*, which resulted in a Gly to Thr substitution, showed some fitness penalty.^78^ This was probably due to reduced natural auxin perception, which could impact competition with crops in the field.^78^, ^134^

We cannot overlook that most fitness studies lack proper control of the genetic background, and their results may reflect natural variation or other factors unrelated to the herbicide‐resistance allele. Ideally, fitness should be evaluated in populations derived from resistant × susceptible crosses or, alternatively, by comparing several resistant populations carrying the same causal resistance allele to several susceptible populations.^123^

From a weed and herbicide resistance management perspective, fitness penalties are a boon. The stronger the penalty, the faster resistant plants (and their alleles) will theoretically be purged from a population with no herbicide selection. If all mutations carried such fitness penalties, herbicide resistance could be solved by switching to other herbicides until the allele frequency dropped to an acceptable level. Unfortunately, severe fitness penalties seem to be the exception, not the rule.^135^ More subtle impacts on seed production and biomass growth may be more common, but the weaker the penalty, the more persistent the TSR mechanism will be in an unselected population, as there is not enough negative selection to purge resistance alleles. Fitness penalties may be exploited by weed scientists and growers for resistance control. The development of more aggressive/competitive crops and utilizing cultural control methods such as cover crops, narrow row spacing, and high seeding rates may challenge herbicide‐resistant crops sufficiently to reduce seed production and weed‐related damage. However, evolutionary processes that generate mutations in other regions of the genome may compensate for the fitness penalties imposed by the resistance allele.^136^, ^137^

### 2.3 Target site increased expression

In addition to mutations altering direct enzyme‐herbicide binding, weeds have evolved a second target site mechanism via the overexpression of the non‐mutated target‐site gene. This overexpression can be driven via additional gene copies (copy number variation (CNV)) or by modifications to the gene expression regulation machinery, such as the promoter, *cis*‐ or *trans*‐elements. The former seems to be a common phenomenon, especially for *EPSPS* in glyphosate resistance, but there are cases for *ACCase* and *GS* as well. The presence of several *EPSPS* and *GS2* copies in non‐chromosomal structures called extrachromosomal circular DNA (eccDNA), conferring glyphosate and glufosinate‐ammonium resistance, respectively, is well‐characterized in *Amaranthus palmeri*.^14^, ^138^, ^139^ In *Bassia scoparia*, *EPSPS* duplication is tandemly arranged in chromosomal DNA at the native locus.^15^, ^140^, ^141^ Other important species, such as *Lolium multiflorum*,^142^ *Amaranthus tuberculatus*,^143^ and *Eleusine indica*,^144^ have been reported with *EPSPS* CNVs, while *Digitaria sanguinalis* populations have been found with *ACCase* CNV.^145^ The presence of multiple gene copies results in more enzyme production, which is the same consequence as the up‐regulation of a single gene. Target gene up‐regulation has been reported for *psbA*, contributing to atrazine resistance; however, this is complicated by the *psbA* gene being chloroplast, and therefore dependent on the number of chloroplasts per cell, a different form of gene CNV.^146^

Many have tried to find fitness penalties associated with CNV; however, if they are there, they seem to be small, so much so that it does not impact management greatly.^147^, ^148^, ^149^ The higher capacity of producing target enzymes requires more herbicide to inhibit all enzyme activity. Increasing the herbicide dose would be effective in controlling plants with a low number of extra copies or a small increase in expression, which would require the assessment of the occurrence and variability at field level. However, the copy number can range from a few^5^, ^6^, ^7^, ^8^, ^9^, ^10^ to hundreds in resistant plants,^14^, ^15^ and continuous spraying at higher dosage would select plants with more copies; moreover, the herbicide dose required would exceed the maximum registered rate. Increasing the dose in these cases is therefore not economically feasible. The herbicide MoA rotation is the best management practice for target site increased expression.

## 3 NON‐TARGET SITE RESISTANCE

### 3.1 Metabolic and vacuolar sequestration resistance are temperature‐dependent

#### 3.1.1 Metabolic resistance

NTSR mechanisms are highly variable but generally include any factor that decreases the concentration of the active herbicide at the site of action, including, but not limited to, increased herbicide metabolism, reduced translocation/sequestration, reduced absorption, and rapid necrosis.^8^ Metabolism‐based mechanisms are the most worrying due to their potential to confer resistance to multiple chemistries, even from very different MoAs and chemical groups, low potential for fitness penalties, and the difficulty in finding the causal gene(s). Different plants have evolved the capacity to metabolize various herbicides around the world. For instance, CYP enzymes are involved in *Echinochloa phyllopogon* resistance to ALS and ACCase‐inhibiting herbicides^150^, ^151^; *Amaranthus tuberculatus* resistance to 2,4‐D^152^; *Echinochloa crus‐galli* resistance to florpyrauxifen‐benzyl^153^; and in *Lolium rigidum* to at least five MoAs.^9^ GSTs have been shown to be the causal enzyme in *Alopecurus myosuroides* multiple herbicide resistance,^154^ *Beckmannia syzigachne* resistance to ALS and ACCase inhibiting herbicides,^155^ and in *Amaranthus palmeri* resistance to S‐metolachlor.^156^ A new metabolism enzyme class, the AKRs, was found in *Echinochloa colona* and *Lolium rigidum* to metabolize glyphosate.^157^, ^158^ In general, the overexpression of metabolism genes is responsible for the increased capacity to detoxify pesticides. Interestingly, metabolic activity has been directly linked to environmental conditions, especially temperature (Table 2).

**Table 2:** Summary of temperature effect on non‐target site resistance (NTSR) to different herbicide modes of action

MoA	Herbicide	Species	Temperature		Reference
			Susceptibility or reduced resistance	Resistance	
ACCase inhibitor	Pinoxaden	*Brachypodium hybridum*	16/10 °C	34/28 °C	^160^
ACCase inhibitor	Cyhalofop‐butyl	*Echinochloa crus‐galli*	25/20 °C	35/30 °C	^159^
ACCase inhibitor	Fenoxaprop and Clodinafop	*Alopecurus myosuroides*	10 °C	25 °C	^163^
EPSPS inhibitor	Glyphosate	*Lolium rigidum*	8 °C	19 °C	^164^
EPSPS inhibitor	Glyphosate	*Lolium perenne*	9 °C	20 °C	^165^
EPSPS inhibitor	Glyphosate	*Sorghum halepense*	19 °C	30 °C	^164^
EPSPS inhibitor	Glyphosate	*Conyza bonariensis*	12 °C	25 °C	^177^
EPSPS inhibitor	Glyphosate	*Conyza canadensis*	8 or 12 °C	20 °C	^178^, ^179^
EPSPS inhibitor	Glyphosate	*Eleusine indica*	20 °C/15 °C	30 °C/25 °C	^166^
EPSPS inhibitor	Glyphosate	*Echinochloa colona*	25 °C/20 °C	35 °C/30 °C	^157^
EPSPS inhibitor	Glyphosate	*Echinochloa colona*	15 °C/10 °C	25 °C/20 °C or 35 °C/30 °C	^168^
PSI inhibitor	Paraquat	*Lolium rigidum*	30 °C	15 °C	^180^
PSI inhibitor	Paraquat	*Hordeum leporinum*	30 °C	15 °C	^181^
Auxinic	2,4‐D	*Amaranthus palmeri*	24/14 °C	34 °C/24 °C	^170^
Auxinic	2,4‐D	*Amaranthus tuberculatus*	24/10 °C	34 °C/20 °C	^171^
HPPD inhibitor	Tembotrione	*Amaranthus palmeri*	18 °C	28 °C	^172^

Abbreviations: ACCase, acetyl‐CoA carboxylase; EPSPS, 5‐enolpyruvylshikimate‐3‐phosphate synthase; PSI, photosystem I; HPPD, 4‐hydroxyphenylpyruvate dioxygenase.

The effect of temperature on herbicide metabolism has been reported to influence resistance to ACCase inhibitors. A cyhalofop‐butyl metabolic resistant *Echinochloa crus‐galli* biotype showed susceptibility to the herbicide when grown at 25 °C/20 °C (day/night) (surviving up to 71 g ha^−1^) but survived twice the label rate (570 g ha^−1^) and showed faster herbicide degradation at 35 °C/30 °C.^159^ This biotype needed at least 48 h of exposure at 35 °C/30 °C to survive the label rate of 285 g ha^−1^.^159^ In a *Brachypodium hybridum* biotype resistant to pinoxaden, high temperatures (34 °C/28 °C day/night) during the first 48 h after herbicide application increase the capacity to detoxify the herbicide when compared with lower temperatures (16 °C/10 °C day/night).^160^ Plants that were grown in low temperatures and transferred to high temperatures at 2 h, 24 h, or 48 h after pinoxaden application survived. In contrast, plants grown in high temperatures and transferred to low temperatures at the same time points after pinoxaden treatment died.^160^ Metabolic evaluation showed that both resistant and susceptible biotypes have high levels of metabolite M5 (pinoxaden‐glucose conjugate) at 48 and 96 h after treatment (HAT) in plants grown under high temperature and, consequently, the metabolite M2 (active molecule) declined more rapidly under high compared with low temperature. However, the resistant biotype showed a faster conversion of the metabolite M2 to M4 (intermediate metabolite) as early as 2 h, and this can be the key factor to enable survival.^160^ RNA sequencing (RNA‐Seq) analysis showed up‐regulation of *CYP* (*CYP72A15* and *CYP72A14*), *GST* (*GST‐TAU18*, *GST‐TAU8*, and *GST‐TAU16*), and *UGT* (*UDPGT‐88A1*, *UDPGT‐73B5*, and *UDPGT‐73B4*) genes in this biotype.^161^ The up‐regulation of metabolic genes after pinoxaden application was found in both high and low temperature conditions, which suggests that the temperature‐dependent pinoxaden resistance may depend more on enzymatic efficiency than on gene regulation.^161^ In the same way, in *Alopecurus myosuroides* resistant to fenoxaprop‐*p*‐ethyl and clodinafop‐propargyl, the GST enzyme activity increased between winter and spring, correlating with reduced herbicide efficiency in the spring when more solar radiation, hours of sunlight, and warmer temperatures occur.^162^ In addition, in *Alopecurus myosuroides* it was found that plants grown at 25 °C have higher GST activity than those grown at 10 °C in the absence of any herbicide for both resistant and susceptible biotypes; however, the temperature effect was more pronounced for the resistant biotype.^163^ Transferring the plants from 25 °C back to 10 °C resulted in a reduction of GST activity without a difference between the resistant and susceptible biotypes.^163^

Resistance to glyphosate due to NTSR can also be influenced by environmental temperatures. In *Lolium rigidum*, a temperature of 8 °C decreased the resistance factor when compared to warm temperature (19 °C); in the same way, *Sorghum halepense* was more sensitive at 19 °C than at 30 °C.^164^ In *Lolium perenne*, temperatures of 20 °C conferred resistance to glyphosate, but temperatures below 10 °C completely reversed the resistance.^165^ In *Eleusine indica*, the dose that caused a 50% reduction in plant growth (GR_50_) at high temperature (30 °C/25 °C) was 3134 g ha^−1^, while at low temperature (20 °C/15 °C) the GR_50_ was 678 g ha^−1^ for the resistant biotype and 353 and 219 g ha^−1^, respectively, for the susceptible.^166^ Glyphosate absorption was 10% and 24% higher at 30 °C/25 °C than at 20 °C/15 °C for the resistant and susceptible biotypes, respectively; the amount translocated was higher at high temperatures (~32%) than at low temperatures (~17%) without differences between susceptible and resistant biotypes.^166^ Recently, the gene *CYP71AK44* has been associated with glyphosate metabolism in *Eleusine indica*.^167^ However, *Echinochloa colona* was the first characterized species that evolved resistance to glyphosate due to metabolism, and several other populations have consistently shown a temperature effect. Plants grown at 25 °C/20 °C or 35 °C/30 °C for 8 days after application were resistant, but when grown at 15 °C/10 °C they died.^168^ In another *Echinochloa colona* biotype, it was verified that the level of glyphosate resistance is highly influenced by temperature. Plants treated with 540 g ha^−1^ and grown at 35 °C/30 °C (day/night) had only 5% reduction in biomass compared with the untreated control, while those grown at 25 °C/20 °C had a reduction of 70%.^157^ The mechanism of resistance identified in this specific biotype was the metabolism gene *AKR4*, which metabolizes glyphosate into AMPA and glyoxylate, as well as the *ABCC8* transporter (described in the next section).^157^, ^169^ *AKR4* gene expression was 2.9 times higher at 35 °C/30 °C than at 25 °C/20 °C in the resistant biotype.^157^, ^169^

Metabolic auxinic herbicide resistance is also temperature‐dependent. In *Amaranthus palmeri*, 2,4‐D resistant plants grown at 34 °C/24 °C had a GR_50_ of 1308 g ha^−1^, and 57 g ha^−1^ for the susceptible; however, a GR_50_ of 131 g ha^−1^ and 63 g ha^−1^, respectively, was verified when grown at 24 °C/14 °C, restoring susceptibility.^170^ The resistant biotype metabolized 2,4‐D faster than the susceptible at 24 HAT under both temperatures, and the amount of remaining 2,4‐D parent compound was lower at high temperature than at low.^170^ Similarly, *Amaranthus tuberculatus* resistant to 2,4‐D also showed a temperature‐dependent resistance level. At high temperature (34 °C/20 °C), the 2,4‐D level of resistance was 3.7 times higher when compared with the low temperature (24 °C/10 °C).^171^ The parent compound disappeared faster at high temperature, 24 and 72 HAT, when compared with low temperature in the resistant biotype.

Increased metabolism associated with 4‐hydroxyphenylpyruvate dioxygenase (HPPD)‐inhibitor resistance in *Amaranthus palmeri* also showed a temperature effect. The resistant population showed a reduction in the amount of tembotrione recovered at 28 °C compared with 18 °C, indicating a faster parent herbicide degradation at high temperature.^172^ According to the metabolites found, tembotrione first had a 4‐hydroxylation reaction, which is typically catalyzed by cytochrome P450 enzymes, followed by glycosylation.^172^ Subsequently, the gene *CYP72A1182* was functionally validated to perform 4‐hydroxylation on tembotrione.^173^

#### 3.1.2 Vacuolar sequestration

Vacuolar sequestration is one of the NTSR mechanisms, limiting the herbicide translocation by trapping it in the vacuole before it reaches the target enzyme.^8^ The main gene family for this mechanism is ABC‐transporters located in the tonoplast membrane. Examples of ABC‐transporters modulating resistance have been reported for *Conyza canadensis* and *Echinochloa colona* resistant to glyphosate,^169^, ^174^ *Lolium rigidum*, *Lolium perenne* spp. *multiflorum*, and *Hordeum leporinum* resistant to paraquat,^175^ and *Alopecurus myosuroides* resistant to mesosulfuron‐methyl.^176^ In the same way as described previously for metabolic resistance, vacuolar sequestration has also been reported as responsive to temperature. In *Conyza bonariensis*, resistant plants survived 1440 g ha^−1^ glyphosate at 25 °C, while at 12 °C the resistant plants were killed at 180 g ha^−1^.^177^ For *Conyza canadensis*, 85% of the glyphosate was in the vacuole 1 day after treatment in a resistant biotype, and only 15% in the vacuole of the susceptible. These results suggest that tonoplast pumps are acting more efficiently in transporting across the tonoplast membrane in resistant individuals.^174^, ^178^ In addition, the amount of glyphosate sequestered in the vacuole at low temperature (12 °C) was lower than at warm temperature (20 °C) in the resistant biotype.^178^ Even if these studies have not investigated the genes that explain the glyphosate resistance in these species, it is likely that the mechanism is a reduced translocation.^164^, ^165^, ^178^ However, a few other studies investigated transcriptomic differential expression. In *Echinochloa colona* resistant to glyphosate and showing temperature‐responsiveness, the gene *ABCC8* acts together with the *AKR4* (described in the previous section), and showed 2.8 times higher expression at 35 °C/30 °C than at 25 °C/20 °C in the resistant biotype.^157^, ^169^ In *Conyza canadensis*, temperature has been shown to change the expression of ABC‐transporters, *M10* and *M11*, making the glyphosate‐resistant biotype sensitive at 8 °C.^179^

Resistance to the herbicide paraquat has also been reported as vacuolar sequestration caused by tonoplast‐membrane transporters in *Lolium perenne* spp. *multiflorum*.^175^ However, opposite to what has been reported for glyphosate, in *Lolium rigidum* ^180^ and *H. leporinum* ^181^ the resistance to paraquat was demonstrated to have the opposite temperature effect compared to glyphosate, with low temperature (15 °C) showing a higher resistant phenotype, and high temperature (30 °C) decreasing the resistance level. In *H. leporinum*, the paraquat resistance affected by temperature is not related to the target site sensitivity or differential absorption. The results suggest a reduced paraquat translocation to the growing tissue and inferior access to the active site.^181^ In *Lolium rigidum*, a restricted paraquat translocation was verified in the resistant biotype compared to the susceptible.^180^ What is still not well understood is why the herbicide resistance caused by reduced translocation to paraquat is verified at low temperatures, whereas for glyphosate at high temperatures.

### 3.2 Microorganisms mediating metabolic herbicide resistance

Recently, the role of microorganism interaction with herbicide‐resistant weed populations has begun to be studied more in‐depth. The mechanism of microorganisms conferring resistance to pesticides is called ‘co‐metabolic degradation.’^182^ The two most conclusive studies investigated resistance to ACCase inhibitors. The endophyte taxa *Pantoea*, *Paenibacillus*, *Acidovorax*, *Sphingomonas*, and *Massilia* were associated with resistance to cyhalofop‐butyl and transmitted across five generations. *Pantoea deleyi* did not have a significant effect on the growth of unstressed *Echinochloa crus‐galli* but significantly decreased herbicide stress after application by activating genes from *CYP* and *GST* families.^183^ It was also found that the presence of *Bacillus velezensis* antagonizes the effect of *Pantoea deleyi*, restoring susceptibility.^183^ In *Polypogon fugax*, the endophyte *Sphingomonas* spp. was found to be more abundant in the quizalofop‐ethyl‐resistant biotypes when compared to susceptible ones, with the ability to degrade more than 90% of quizalofop‐ethyl *in vitro*.^184^ Susceptible plants inoculated with *Sphingomonas* spp. increased their resistance factor eight times, while the resistant biotype treated with endophyte growth inhibitors reduced their resistance factor three times.^184^ The same co‐metabolic degradation effect of microbes and weeds has also been identified in insects, resulting in increased metabolism of insecticides.^185^ The two prevailing hypotheses are that microorganisms either induce plant genes to degrade the herbicide or do the metabolism themselves. Methodological procedures should be designed considering the weed × microorganism interaction(s), for example, filtering out differentially expressed genes from microorganisms in RNA‐Seq analysis.^186^

Other studies have also associated herbicide resistance with microorganisms. In Italy, soils with a higher incidence of ALS or ACCase‐resistant *Echinochloa* spp. showed a lower microbial diversity, while soils with a lower incidence of resistant *Echinochloa* spp. showed a higher microbial diversity.^187^ This study demonstrated that soils with a high incidence of resistance were positively associated with microorganisms in the *Lactobacillales*, *Malasseziales*, and *Diaporthales* clades, whereas *Rhizobiales* and *Solibacterales* were associated with soils exhibiting low resistance.^187^ A different study also found higher diversity of rhizosphere microbiota associated with penoxsulam‐susceptible rather than penoxsulam‐resistant *Echinochloa crus‐galli*.^188^ *Acidobacteria*, *Bacteroidetes*, *Gemmatimonadota*, and *Proteobacteria*, *Ascomycota*, *Chytridiomycota*, and *Rozellomycota* were present at higher relative abundance in penoxsulam‐resistant compared with susceptible plants.^188^ In addition, root exudates from resistant and susceptible plants modulated microorganism abundance and diversity in the rhizosphere.^188^ However, these studies often focus on the microbiome and lack in sequencing the herbicide target gene to show the absence of TSR, and generally lack experiments that conclusively link certain microbes with resistance, such as inoculating susceptible plants, which would make the association of microorganisms with herbicide resistance more robust. The actual effect of microorganisms conferring herbicide resistance, or whether it is a small contribution to the plant detoxification system, still needs additional research.

### 3.3 Rapid necrosis is herbicide‐specific and may affect herbicide mixtures

One of the most unusual NTSR mechanisms discovered recently is ‘rapid necrosis.’ Plants with this mechanism respond almost immediately to the application of herbicide, undergoing rapid necrosis of their foliar tissue, which is then shed, allowing the plants to regrow from meristematic tissue. This is especially effective for systemic herbicides like glyphosate and 2,4‐D, which require time to translocate to growing points and effectively kill the whole plant, effectively sequestering most of the active ingredient in desiccated tissue. This mechanism has only been reported in two Asteraceae weed species: *Ambrosia trifida* for glyphosate^189^ and *Conyza sumatrensis* for 2,4‐D.^190^, ^191^ In rapid necrosis‐based glyphosate‐resistant *Ambrosia trifida*, the symptoms appear over 1 or 2 days and are mainly in mature leaves, with young leaves remaining less affected, which helps restart growth after treatment.^192^ In 2,4‐D‐resistant *Conyza sumatrensis*, the rapid necrosis symptoms appear on leaves within a few hours, and after 1 day the plants are completely desiccated; however, 21 days after the application, the plants start to regrow from the axillary buds.^190^, ^191^ Rapid necrosis in this 2,4‐D‐resistant *Conyza sumatrensis* biotype seems to be 2,4‐D specific, as the other auxinic herbicides fluroxypyr, triclopyr, picloram, dicamba, florpyrauxifen‐benzyl, and halauxifen‐methyl did not generate the rapid necrosis symptoms.^190^ One consequence of this mechanism is that if 2,4‐D is used in a tank‐mix with other herbicides, the 2,4‐D will trigger the mechanism, sequestering all the active compounds in the dead tissue, preventing their translocation. The effectiveness of dicamba, triclopyr, and halauxifen‐methyl is reduced when sprayed on the resistant biotype together with, 4 h after, or 24 h after 2,4‐D application.^193^ Researchers also noted that decreased light intensity and low temperatures (12 °C) decreased the velocity of the beginning of the rapid necrosis symptoms^190^, ^193^; then, mixtures of herbicides applied at night can improve translocation of tank‐mixed herbicides and still provide control of resistant *Conyza sumatrensis*. A sequential application of other herbicides when the *Conyza sumatrensis* starts to regrow is also an efficient option. In *Ambrosia trifida*, a mixture of other herbicides with glyphosate may be efficient, because young leaves are less affected and can still translocate the other active herbicides. While this mechanism is fairly limited in terms of species distribution and herbicide molecules, its uniqueness and importance in the systems in which it occurs make it worthy of note. Only through careful study of these mechanisms is it possible to develop alternative recommendations to mitigate them.

### 3.4 Implications for management

It is clear that high temperatures greatly affect enzyme *K*_m_ (Michaelis constant, represents the substrate concentration at which the reaction rate reaches half of its maximum velocity) and *V*_max_ (maximum reaction velocity) and, consequently, generally enhance the efficiency of enzymatic processes^194^, ^195^ (Fig. 2). What this means is that resistance, especially metabolic and transporters resistance to glyphosate, 2,4‐D, or ACCase inhibitors, may be manageable at low temperatures. If the weed species are from warm‐season crops, for example *Conyza* sp. and *Amaranthus* sp., early burndown can be recommended to take advantage of low‐temperature periods; if the weeds are commonly found in winter crops, such as *Lolium* spp., low‐temperature periods are common during most of the crop cycle, which would help to manage metabolic resistant populations. In these cases, applications could be timed with periods of predicted low temperatures. In the case of paraquat resistance, high temperatures decrease resistance. To date, just a few metabolism‐based herbicide resistance studies have been performed at different temperatures, but it is possible that many of them are temperature‐sensitive. The general trend of increased resistance at higher temperatures means that enhanced metabolic resistance is likely to be more evident due to the predicted global warming, and that periods of low temperature may be less available for exploitation.^196^ Understanding exactly how NTSR mechanisms operate and under which conditions they occur can be crucial for developing herbicide resistance management plans and enabling the exploitation of these findings. In addition, phenotypic resistance diagnosis may produce false negatives if the effects of temperature on metabolic resistant populations are not considered during glasshouse screenings.

**Figure 2:**
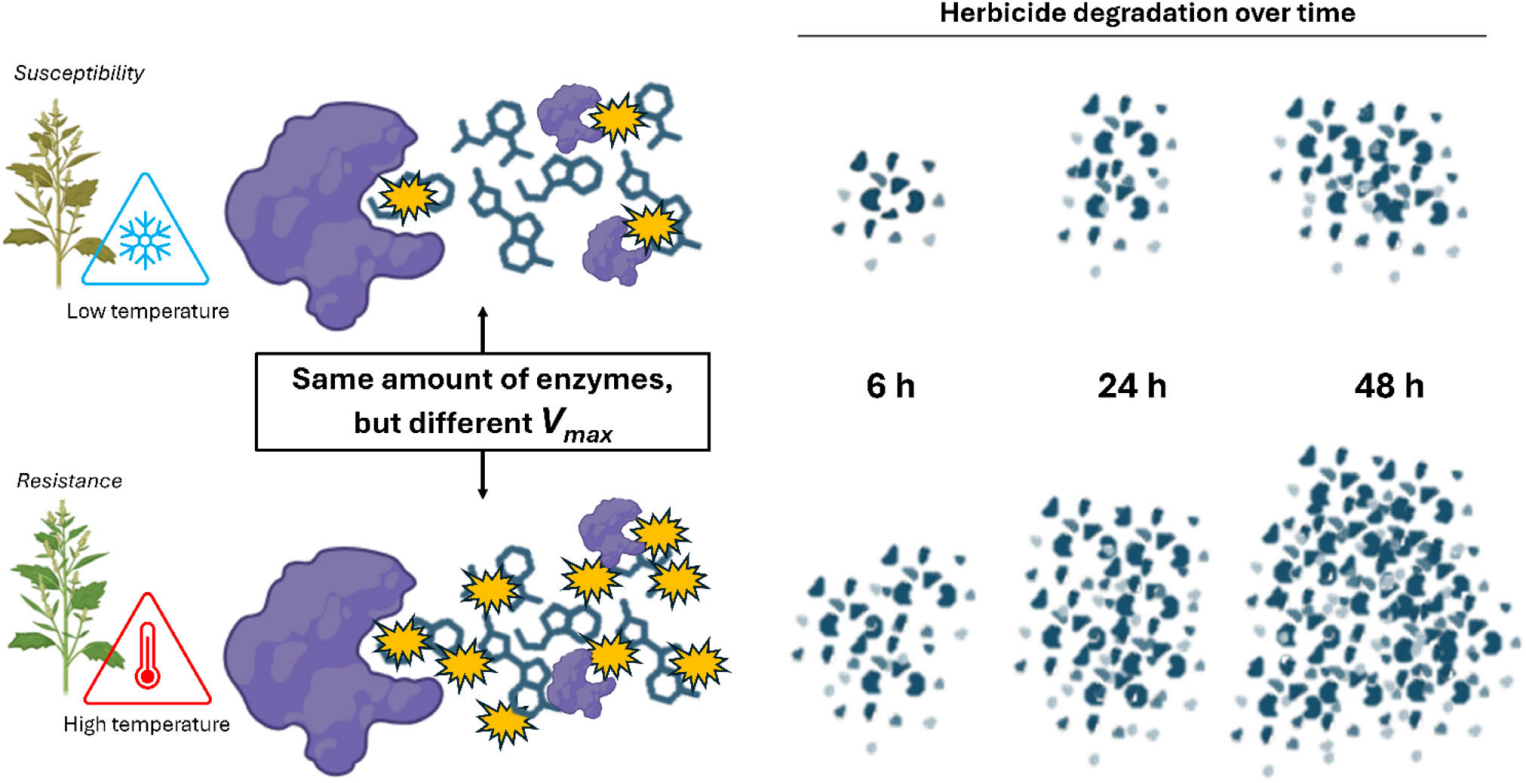
Temperature effect on metabolic resistance. A population with metabolic enzyme overproduction exhibits different phenotypes depending on its interaction with environmental temperature. In cases where there is a genotype × environment interaction, enzyme overproduction alone may not be sufficient to metabolize the herbicide quickly enough to prevent it from reaching the target site. However, when enzyme overproduction occurs under higher temperatures, the resulting increase in enzyme activity (higher *V*_max_) enables faster herbicide metabolism, leading to the resistant phenotype. Image created with BioRender.

Metabolic‐ and transporter‐mediated resistance exhibit unpredictable cross‐resistance patterns, which complicates the design of effective herbicide rotation programs. This apparent unpredictability may partly reflect the limited exploration of these mechanisms, as the validation and functional characterization of herbicide‐resistance genes, particularly those involved in metabolism and transport, are relatively recent. As more candidate genes are validated and their substrate specificities clarified, it is likely that clearer patterns of cross‐resistance will emerge, enabling more informed and predictive resistance‐management strategies.

Rapid necrosis is the most recently identified NTSR mechanism, and no causal gene has been validated to date. Because only a limited number of studies are available, management recommendations remain scarce. At present, it is advised to avoid tank‐mixing herbicides that trigger rapid necrosis with other herbicides. However, if such mixtures are used, applying them at night, when temperatures and light intensity are lower, can delay the onset of necrosis and may allow greater herbicide translocation before tissue collapse occurs.

The knowledge of microorganisms acting as promoters of metabolic herbicide resistance would bring the opportunity to utilize compounds to synergize with herbicides, such as antibiotics, fungicides,^186^ or inoculation with other microorganisms.^183^ In addition, a better understanding of this phenomenon may lead to microbial community monitoring in fields to help predict herbicide failure. It would also be possible to implement management strategies to shift microbial composition or to slow the microbial dissemination.^185^

## 4 NEGATIVE CROSS‐RESISTANCE AS A STRATEGY TO DELAY OR PREVENT RESISTANCE

When a mechanism that confers resistance to one herbicide increases the susceptibility to another herbicide, it is called negative cross‐resistance (NCR).^197^ The NCR phenomenon is better understood for insecticide resistance mechanisms,^198^ but studies into NCR in weeds are increasing.^199^, ^200^ NCR is usually characterized for TSR mechanisms, due to variation in key‐lock binding affinity (Fig. 3(A)). For example, in *Cyperus difformis* the mutation Val219Ile in *psbA* gene (which encodes the D1 protein) confers resistance to propanil (amides), bromoxynil (nitriles), metribuzin (triazinones), and diuron (ureas) but increases the sensitivity of the resistant population to bentazon (benzothiadiazinone) by four times when compared with the susceptible.^92^ The *psbA* mutation Ser264Gly confers resistance to atrazine but increases the susceptibility to bromoxynil in *R. raphanistrum*.^89^ All these mentioned herbicides are PSII inhibitors belonging to different chemical groups and are separated into two HRAC MoAs (Groups 5 and 6). Bentazon and bromoxynil specifically bind to a different site, His215 on the D1 protein (Group 6), while the others are Ser264 binders (Group 5).^201^

**Figure 3:**
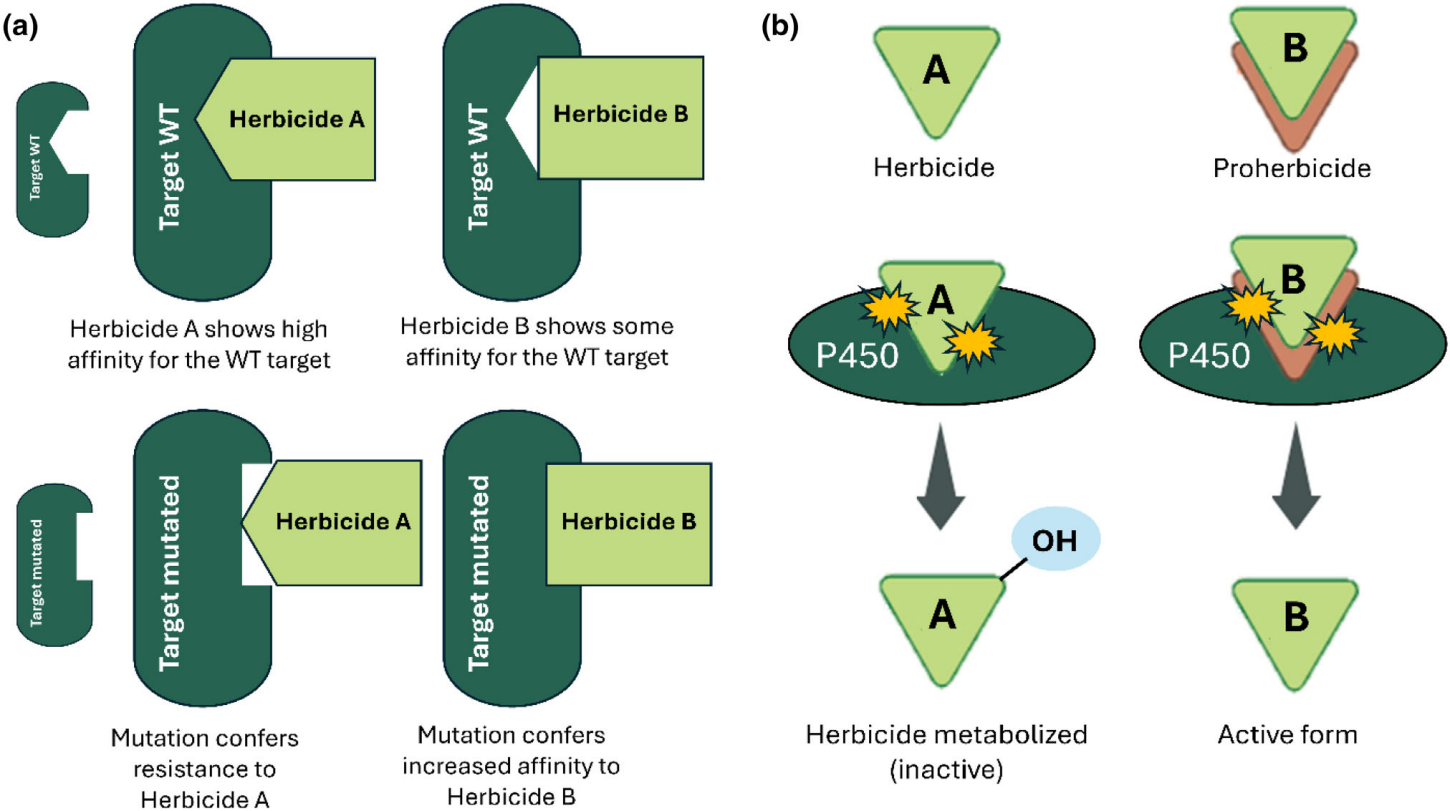
Negative cross‐resistance related to target and non‐target site resistance. (a) The wild‐type version of the enzyme is susceptible to binding by herbicides A and B. A target‐site mutation alters the enzyme's binding pocket, conferring resistance to herbicide A; however, the same mutation that prevents herbicide A from binding increases the binding affinity of herbicide B, making the enzyme more susceptible than the wild‐type. (b) Overproduction of metabolic enzymes (e.g., cytochrome P450 monooxygenases) with affinity for herbicide A results in its rapid metabolism to an inactive form, preventing it from reaching the target site. However, the same overproduced enzymes can metabolize proherbicide B to its active form more quickly than in plants without enzyme overproduction, thereby increasing susceptibility.

Amino acid replacements that result in herbicide resistance sometimes have no impact on native protein function (i.e., no fitness penalty), however, they can change the protein conformation and increase the affinity to other herbicides within the same MoA.^202^ For example, four *Amaranthus hybridus* populations resistant to imazethapyr (IMI) with Ala122Thr mutation in *ALS* gene have increased susceptibility to cloransulam‐methyl (TP) and pyrithiobac‐sodium (PB), all ALS‐inhibitors.^31^, ^32^ The GR_50_ of the susceptible population to imazethapyr was 1.5 g ha^−1^, while in the resistant ones ranged from 1022 to 1890 g ha^−1^; for cloransulam‐methyl, the GR_70_ of the susceptible population was 130 g ha^−1^, while for the resistant ones ranged from 12 to 67 g ha^−1^.^31^ The ALS enzymatic assay showed the imazethapyr‐resistant enzyme with Ala122Thr required 6.5 times lower concentration of pyrithiobac‐sodium to inhibit 50% of enzymatic activity (*I*_50_) than the ALS wild‐type.^203^ A biotype of *Descurainia sophia* resistant to tribenuron‐methyl (SU) with Pro197Ser mutation in *ALS* gene exhibits NCR to imazethapyr (IMI), while other amino acid replacements at the same position Pro197His/Thr/Ala/Leu do not.^200^ The mutant ALS showed an *I*_50_ of 50 μm to tribenuron‐methyl, while the wild‐type ALS showed an *I*_50_ of 0.18 μm; however, for the herbicide imazethapyr, the *I*_50_ for the mutant ALS was 2.65 μm, while for the wild‐type was 4.51 μm, representing 70% more.^200^ In the same study, the NCR was not observed for other imidazoline herbicides like imazamox and imazapic, which reinforces that even inside the same chemical group, there is enzyme binding variation.^200^

NCR can also be exploited for NTSR mechanisms like metabolism. As described earlier, these NTSR often detoxify several compounds from different MoAs,^204^ making the efficacy of herbicide rotation with different MoAs unpredictable. However, the characterization of increased metabolism in populations also allows for the rotation with other herbicides that have their effectiveness increased by being metabolized, known as ‘proherbicides’ (Fig. 3(B)). Proherbicides are compounds that do not have herbicidal activity in their primary structure and utilize the plant's or microorganisms’ enzymatic activity, or environmental factors, to convert them into their active form.^205^ These can be beneficial as their inactive proherbicide form may have better chemical properties for absorption and translocation, with metabolism to the active form upon arrival at the cellular environment. Examples of proherbicides are molecules formulated as esters that need to lose their methyl, benzyl, butyl, or ethyl group to be active in their acid form, for example, cyhalofop‐butyl (ACCase inhibitor).^206^ The most classic proherbicide is clomazone (1‐deoxy‐d‐xylulose 5‐phosphate synthase (DXP) inhibitor). Clomazone bioactivation has two steps: the first is the conversion to 5‐OH‐clomazone, and the second to 5‐keto‐clomazone, which is the active form.^207^ This bioactivation is driven by CYP enzymes.^208^ Four biotypes of *Echinochloa crus‐galli* resistant to IMI herbicides via enhanced metabolism increased their susceptibility approximately three times to clomazone when compared with four susceptible biotypes.^199^ The reason is the IMI‐metabolizing *CYP* genes also activate significantly more of the proherbicide clomazone than in susceptible biotypes. The application of CYP inhibitor before clomazone reverted the NCR, and the clomazone GR_50_ between imazethapyr‐resistant and imazethapyr‐susceptible biotypes was similar.^199^ Corroborating these results, the *CYP709C69* gene that metabolizes diclofop‐methyl in *Echinochloa phyllopogon* increased the susceptibility to clomazone when overexpressed in transformed *Arabidopsis* compared to the wild‐type.^209^ Considering the recent identification of increased metabolic resistance via CYPs in several weed species, the inclusion of clomazone in the herbicide program is recommended (when it is safe for the crop), due to the occurrence of NCR and the low number of resistance cases reported to it.^2^, ^210^, ^211^ Another example of NCR was found in *Sorghum bicolor* resistant to chlorsulfuron (ALS‐inhibitor) with increased metabolism by CYPs, which had its susceptibility to tembotrione and mesotrione (HPPD‐inhibitors) increased when compared with the chlorsulfuron susceptible; however, the underlying reasons for this remain unclear.^212^

Knowledge about the resistance mechanisms, and NCR in particular, allows rational herbicide rotation programs or herbicide mixtures that may otherwise be unexplored. A deep knowledge of resistance mechanisms and specific agronomic systems is critical because not all target‐site mutations or NTSR mechanisms identified are investigated for NCR. For example, there are no reported NCR studies for *ACCase* or *PPO* gene mutations. The NCR concept may be helpful for avoiding or delaying pesticide resistance evolution, or for managing the current resistant biotypes.

## 5 OTHER MANAGEMENT STRATEGIES BASED ON HERBICIDE RESISTANCE MECHANISM KNOWLEDGE

Mixing and rotating herbicides from different MoAs is the most recommended strategy to delay herbicide resistance in the field.^213^ This recommendation assumes that each herbicide will select weed populations in novel directions, and for unique mutations – two unique mutations co‐occurring in the same individual plant should be exponentially rarer than a single mutation. This strategy is well supported when considering only TSR mechanisms, also called specialist mechanisms, because they are specific to a single herbicide MoA. Mutations in single genes causing phenotypic changes are approximated at 10^−5^–10^−6^ per gamete per generation, at least in *Zea mays*,^214^ which corresponds to 10^−8^–10^−9^ changes per base pair in a gene of 1000 nucleotides^215^; in *Lolium rigidum*, the frequency of individuals resistant to the sulfonylurea herbicide sulfometuron‐methyl varied from 1.2 × 10^−4^ to 2.2 × 10^−5^.^216^ Assuming resistance evolution to each herbicide in the mixture is independent, the probability of a double mutation in a single individual is a result of the product of their probabilities, disregarding the occurrence of gene flow between different populations. As a general example, two herbicides targeting different enzymes and a 1 × 10^−5^ probability of a resistance mutation for each target site, result in a probability of 1 × 10^−10^ of the same plant evolving resistance to both herbicides.^217^ If there is a greater rotation of herbicide mixtures, the chance of TSR will drop even more, again multiplicatively.^218^ This is rarely the case in reality, farmers either do not have many options for effective herbicide mixtures (depending on the crop), or it is considerably more expensive as multiple products need to be purchased and applied. Also, it should be noted that if both herbicides in the mixture do not target the same weed species, they do not have any effect in delaying the resistance evolution.^217^ An example of an ineffective mixture is ACCase inhibitors + bentazon to control *Cyperus* spp., because only bentazon has activity on the target weed.

NTSR, specifically metabolic resistance, is often called a ‘generalist resistance mechanism’ and violates the model of resistance described earlier. There is growing evidence that the repetitive use of herbicide mixtures decreases the probability of TSR but increases a more generalist resistance response.^219^ The enzymes CYP, GST, ABC, and others are considered promiscuous, which allows them to bind to a variety of compounds, including herbicides from different MoAs. To date, 60 genes (33 *CYP*s, 12 *GST*s, 5 *UGT/GT*, 5 *ABC*s, 2 *AKR*s, 1 *CASPL* (*Casparian strip domain*‐like), 1 *HIS* (*Fe(II)/2‐ketoglutarate (2‐OG)‐dependent dioxygenase*), and 1 *KCS* (*3‐ketoacyl‐CoA synthase*) have been validated as conferring metabolic herbicide resistance in 28 different species to 53 herbicides (Table 3). Some are involved in resistance to different MoAs, such as *CYP81A10v7* from *Lolium rigidum*,^9^ while others are specific to one herbicide, for example, *AKR* to glyphosate.^157^ In addition, some metabolic enzymes can inactivate only one or a few herbicides from the same chemical group or MoA. For example, *CYP709C69* conferred resistance to diclofop‐methyl but not to cyhalofop‐butyl, both from the FOPs chemical group of ACCase‐inhibitors.^220^ Application of fenoxaprop‐*p*‐ethyl formulated with the safener isoxadifen‐ethyl confers resistance in a specific *Echinochloa crus‐galli* biotype to fenoxaprop through GST detoxification, while fenoxaprop not formulated with the safener and other ACCase herbicides are still effective.^221^ The knowledge of the metabolic mechanisms in weeds and their geographical spread would help make decisions on what herbicides should be utilized. For that, it is also important to screen as many herbicides as possible when validating new metabolic genes. A rational mixture would include herbicides that are not metabolized by the enzymes identified, or even herbicides with a very low chance of evolving metabolic resistance, such as glufosinate‐ammonium, for which only a few metabolic resistance cases have been reported.^222^

**Table 3:** Genes from weeds and crops validated as conferring herbicide resistance

Gene	Species	Herbicide	Reference
*ABCC1*	*Alopecurus myosuroides*	Mesosulfuron‐methyl	^176^
*ABCC2*	*Alopecurus myosuroides*	Mesosulfuron‐methyl	^176^
*ABCC3*	*Lolium rigidum; Lolium multiflorum*	Glyphosate	^263^
*ABCC8*	*Echinochloa colona*	Glyphosate	^169^
*ABCG15*	*Cyperus rotundus*	Glyphosate	^264^
*AKR1*	*Lolium rigidum*	Glyphosate	^158^
*AKR4*	*Echinochloa colona; Lolium rigidum*	Glyphosate	^157^, ^158^
*CASPL2C2*	*Cyperus rotundus*	Glyphosate	^264^
*CYP51*	*Polypogon fugax*	Glyphosate	^265^
*CYP71A1*	*Echinochloa oryzoides*	Thiobencarb	^249^
*CYP71AF43*	*Alopecurus myosuroides*	Fenoxaprop‐*p*‐ethyl	^266^
*CYP71AK44*	*Eleusine indica*	Glyphosate	^167^
*CYP72A15*	*Raphanus raphanistrum*	Mesotrione	^267^
*CYP72A15*	*Echinochloa phyllopogon*	Penoxsulam	^268^
*CYP72A31*	*Oryza sativa*	Bispyribac‐sodium	^269^
*CYP72A385*	*Echinochloa crus‐galli*	Florpyrauxifen‐benzyl	^153^
*CYP72A1182*	*Amaranthus palmeri*	Tembotrione	^173^
*CYP77B34*	*Descurainia sophia*	Tribenuron‐methyl; Carfentrazone‐ethyl; Oxyfluorfen	^270^
*P450‐N‐Z1 (CYP81A)*	*Cynodon dactylon*	Nicosulfuron, Penoxsulam; Mesotrione; Dicamba; Bentazon; Atrazine; 2,4‐D; Flazasulfuron	^271^
*CYP81A6*	*Oryza sativa*	Bensulfuron‐methyl; Bentazon; Sulfonylureas; Clomazone	^272^, ^273^, ^274^, ^275^
*CYP81A9 (Nsf1)*	*Zea mays*	Nicosulfuron; Mesotrione	^276^, ^277^, ^278^
*CYP81A10v7*	*Lolium rigidum*	Diclofop‐methyl; Tralkoxydim; Chlorsulfuron; Mesotrione; Atrazine; Chlorotoluron	^9^
*CYP81A12*	*Echinochloa phyllopogon*	Bensulfuron‐methyl; Penoxsulam; Pyrimisulfan; Propoxycarbazone‐sodium; Azimsulfuron; Pyrazosulfuron‐ethyl; Chlorsulfuron; Propyrisulfuron; Mesotrione; Bentazon; Clomazone; Diclofop‐methyl; Tralkoxydim; Pinoxaden; Quinclorac; 2,4‐D; Florpyrauxifen‐benzyl	^150^, ^151^, ^204^, ^211^, ^279^
*CYP81A14*	*Echinochloa phyllopogon*	Pyriftalid; Propoxycarbazone‐sodium; Pyrazosulfuron‐ethyl	^204^
*CYP81A15*	*Echinochloa phyllopogon*	Clomazone; Pyriftalid; Pyrimisulfan; Azimsulfuron; Pyrazosulfuron‐ethyl; Propyrisulfuron; Bensulfuron‐methyl; Bentazon	^204^, ^211^
*CYP81A18*	*Echinochloa phyllopogon*	Pyriftalid; Propoxycarbazone‐sodium; Azimsulfuron; Pyrazosulfuron‐ethyl; Propyrisulfuron; Bensulfuron‐methyl	^204^
*CYP81A21*	*Echinochloa phyllopogon*	Bensulfuron‐methyl; Penoxsulam; Pyrimisulfan; Propoxycarbazone‐sodium; Azimsulfuron; Pyrazosulfuron‐ethyl; Chlorsulfuron; Propyrisulfuron; Bentazon; Clomazone; Diclofop‐methyl; Tralkoxydim; Pinoxaden; Quinclorac; 2,4‐D; Florpyrauxifen‐benzyl	^150^, ^151^, ^204^, ^211^, ^279^
*CYP81A24*	*Echinochloa phyllopogon*	Pyrimisulfan; Imazamox; Propoxycarbazone‐sodium; Azimsulfuron; Pyrazosulfuron‐ethyl; Chlorsulfuron; Propyrisulfuron; Bensulfuron‐methyl; Penoxsulam; Norflurazon; Mesotrione; Pyraclonil; Bentazon; Clomazone; Tralkoxydim; Pinoxaden; Quinclorac	^150^, ^204^, ^211^, ^279^
*CYP81A63*	*Hordeum vulgare*	Diclofop‐methyl; Pinoxaden	^150^
*CYP81A68*	*Echinochloa crus‐galli*	Penoxsulam; Cyhalofop‐butyl; Metamifop	^280^
*CYP81A104*	*Eleusine indica*	Imazamox; Tribenuron‐methyl; Penoxsulam; Flucarbazone‐sodium; Bentazon; Mesotrione; MCPA	^281^
*CYP81Q32*	*Leptochloa chinensis*	Tripyrasulfone	^282^
*CYP89B16*	*Echinochloa crus‐galli*	Cyhalofop‐butyl; Pinoxaden; Penoxsulam	^283^
*CYP96A146*	*Descurainia sophia*	Tribenuron‐methyl	^284^
*CYP99A2*	*Aegilops tauschii*	Pyroxsulam	^285^
*CYP99A44*	*Beckmannia syzigachne*	Mesosulfuron‐methyl	^286^
*CYP704A177*	*Beckmannia syzigachne*	Pyroxsulam; Bispyribac‐sodium	^286^
*CYP704C1*	*Beckmannia syzigachne*	Fenoxaprop‐*p*‐ethyl; Mesosulfuron‐methyl; Isoproturon	^287^
*CYP709B1*	*Beckmannia syzigachne*	Fenoxaprop‐*p*‐ethyl; Mesosulfuron‐methyl; Isoproturon	^287^
*CYP709C56*	*Alopecurus aequalis*	Mesosulfuron‐methyl; Penoxsulam	^288^
*CYP709C69*	*Echinochloa phyllopogon*	Diclofop‐methyl	^209^
*CYP749A16*	*Gossypium hirsutum*	Trifloxysulfuron	^289^
*GST1*	*Echinochloa phyllopogon*	Penoxsulam	^290^
*GST4*	*Echinochloa crus‐galli*	Penoxsulam	^291^
*GSTF1*	*Alopecurus myosuroides*	Chlorotoluron; Fenoxaprop‐*p‐*ethyl	^154^
*GSTF2*	*Amaranthus tuberculatus*	Atrazine	^292^
*GSTF2*	*Polypogon fugax*	Quizalofop‐*p*‐ethyl	^293^
*GSTF13*	*Alopecurus aequalis*	Fenoxaprop‐*p‐*ethyl	^294^
*GSTU*	*Lolium rigidum*	Flufenacet	^295^
*GSTU1*	*Digitaria sanguinalis*	Haloxyfop‐methyl	^296^
*GSTU17*	*Monochoria korsakowii*	Bensulfuron‐methyl	^297^
*GSTU18*	*Vigna radiata*	Fomesafen	^298^
*GSTU23*	*Echinochloa crus‐galli*	Metamifop	^299^
*GSTU23*	*Echinochloa oryzoides*	Thiobencarb	^249^
*GSTU163*	*Alopecurus japonicus*	Mesosulfuron‐methyl	^300^
*GSTU181*	*Lolium rigidum*	Diclofop‐methyl; Clodinafop‐propargyl; Glufosinate ammonium	^301^, ^302^
*GT1*	*Oryza sativa*	Glyphosate	^303^
*GT8*	*Alopecurus japonicus*	Fenoxaprop‐*p‐*ethyl	^304^
*UGT79‐like*	*Echinochloa crus‐galli*	Quinclorac	^305^
*UGT84A1*	*Descurainia sophia*	Tribenuron‐methyl	^306^
*UGT91C1*	*Arabidopsis thaliana*	Sulcotrione	^307^
*HIS1* (generic name)	*Oryza sativa*	*β*‐Triketone herbicides	^308^
*KCS*	*Eleusine indica*	Paraquat	^11^

Abbreviations: ABC, ATP‐binding cassette; AKR, aldo‐keto reductase; CASPL, casparian strip domain‐like; CYP, cytochrome P450 monooxygenase; GST, glutathione‐S‐transferase; HIS1, generic name ‘HPPD inhibitor sensitive 1’ for a gene that codifies a Fe(II)/2‐ketoglutarate (2‐OG)‐dependent dioxygenase; KCS, β‐ketoacyl‐CoA synthase 11‐like; UGT and GT, UDP‐glycosyltransferase.

The identification and characterization of metabolic resistance also present the possibility of adding metabolic inhibitors mixed with herbicides to restore population susceptibility. The most common CYP inhibitor utilized in research is malathion, an organophosphate insecticide registered in several countries, while the most common GST inhibitor is NBD‐Cl (4‐chloro‐7‐nitrobenzofurazan). In some cases, not all, the metabolic inhibitor synergizes with the herbicide and restores activity, such as the study reporting CYP inhibitors reversing the resistance of *Amaranthus tuberculatus* to tembotrione.^223^ NBD‐Cl application before chlorotoluron, clodinafop‐propargyl, or fenoxaprop‐*p*‐ethyl reversed resistance in *Alopecurus myosuroides*.^154^ These molecules are used in the laboratory as part of validating the involvement of CYPs and GSTs, but rarely, if ever, in the field. It should be noted that these particular inhibitors are highly toxic to animals as well, only serving as an example of the value of metabolic inhibitors. There is an opportunity to develop new metabolic inhibitors that are less toxic and efficient in inhibiting herbicide metabolic enzymes, synergists for herbicide activity in difficult‐to‐control NTSR populations. As an example, the development of GSTF1 crystal structure from *Alopecurus myosuroides*, a well‐known enzyme involved in herbicide detoxification, identified several flavonoids as potential new inhibitors using ligand‐fishing experiments.^224^ One of these compounds reversed the *Alopecurus myosuroides* resistance to pendimethalin.^224^

Additional strategies to effectively control resistant populations could rely on the modality of herbicide application (e.g., pre‐ *versus* post‐emergence application). In *Echinochloa crus‐galli* with target site mutation, penoxsulam was six times more effective in pre‐ than in post‐emergence in biotypes with *ALS* mutation Trp574Leu, whereas imazethapyr was more effective in pre‐emergence in biotypes with the Ser653Asn mutation.^37^ In *Amaranthus tuberculatus* populations resistant to PSII inhibitors, the pre‐emergent application of metribuzin was efficient in controlling populations with metabolic resistance, but not the population with Ser264Gly mutation in the *psbA* gene.^225^ Despite metribuzin being a classic pre‐emergent herbicide, its post‐emergence application demonstrated a lower ED_50_ and ED_80_ for the metabolic‐resistant population than the target site resistant population.^225^ This study supports the hypothesis that metabolic‐resistant populations to post‐emergence herbicides may be more susceptible to them if sprayed as pre‐emergence, but the literature still lacks sufficient data. When considering only post‐emergence application, the plant development stage is also important and may affect the resistant phenotype dramatically. For example, *Amaranthus albus* populations resistant to rimsulfuron with a Pro197Leu *ALS* mutation showed an ED_50_ ranging from 6 to 9 g ha^−1^ when sprayed at 2–4 leaf stage, whereas it ranged from 600 to 900 g ha^−1^ when sprayed at 5–10 leaf stage.^226^ It has been speculated that metabolic‐resistant populations would also be susceptible or less resistant when sprayed with the herbicide at a very early growth stage, due to not fully developed metabolic activity, but this has not yet been shown conclusively.

## 6 QUICK RESISTANCE DIAGNOSTICS TO TAKE ACTION IN THE FIELD

Traditionally, confirming resistance meant collecting seeds and conducting glasshouse bioassays over several weeks. A very optimistic test would take 14 days from germination to spray (if not facing seed dormancy issues), and an additional 21 days for evaluation of product performance. Rapid diagnostic tools are desired to develop and identify resistance within the same season, providing answers within days, before spraying the herbicide to verify the failure of control. Each resistant phenotype has a genomic signature, and the quick assays would capture those particularities. However, the previous identification and characterization of herbicide resistance in weeds is required to implement this tool. The early detection of resistance has many benefits, primarily making the correct decision when choosing herbicides to spray in that season, and decreasing costs by avoiding the use of herbicides that would not perform.

Some quick tests have been developed, shortening the time to diagnose resistance when compared to a regular screening or dose–response curve assays in a glasshouse, such as leaf‐disk assays. This test refers to collecting leaf disks from plants and incubating them for 24–48 h in an herbicide solution in plates. The evaluation is made utilizing the chlorophyll fluorescence (*F*_v_ */F*_m_) parameter; the higher the *F*_v_ */F*_m_ value, the less affected by the herbicide the plant was, meaning resistance.^227^ This method is broad and does not provide any insights regarding the mechanism of resistance. In addition, it still needs improvements, such as standardized protocols for testing different species, timing of incubation for different herbicide MoAs, and doses to discriminate resistant × susceptible populations.

In terms of tests that provide more complete information, TSR is the most feasible mechanism to develop quick‐resistance testing compared to NTSR. Some tests for TSR have already been developed, such as a biochemical assay to quantify the amount of herbicide target enzyme's substrate accumulation. This is a very common assay to characterize the TSR mechanism; however, it does not provide any information about the specific TSR mechanism (i.e., what mutation or if CNV). This assay has been used mainly to detect shikimate accumulation, the EPSPS enzyme substrate, in glyphosate resistance tests^228^, ^229^ and to measure the enzymatic activity in the presence of the herbicide, such as the ALS enzyme activity through acetolactate production.^230^ The lower the substrate quantified, the less effective the herbicide is in inhibiting that enzyme, which means resistance. These methods have the advantage of generating results in a few days and require only a few leaves. The disadvantage is the incomplete information about what the TSR mechanism is, and potential false‐negative results, in the case of using the substrate accumulation method for an NTSR mechanism.

Considering the large knowledge in the TSR area, molecular markers have been developed for quick genotyping.^16^, ^17^, ^231^ Once those DNA changes are known, we can test them directly. The process involves collecting a small leaf sample from the suspect resistant weed and sending it to a laboratory. DNA is extracted and analyzed (via polymerase chain reaction (PCR) or quantitative real‐time PCR (qPCR)) for known mutations or gene copy number that confer resistance, which would take a few days from shipping to the results (Fig. 4). These tests have high accuracy and typically report whether specific resistance alleles were ‘detected’ or ‘not detected’ in the sample. However, they only detect the target sequence polymorphism. If a known mutation is found, that resistance mechanism is confirmed. If none are found, the weed might be susceptible or might have a non‐target‐site mechanism that the DNA test cannot identify. Unknown mechanisms or metabolic resistances will evade a targeted PCR test. Despite this, as the knowledge of resistance genetics expands, more mechanisms are becoming ‘known’ and testable. Molecular diagnostics shine in providing mechanistic certainty. They tell us what mutation or gene amplification is present. This is valuable for making informed management decisions, such as understanding cross‐resistance patterns, as explained earlier in this review.

**Figure 4:**
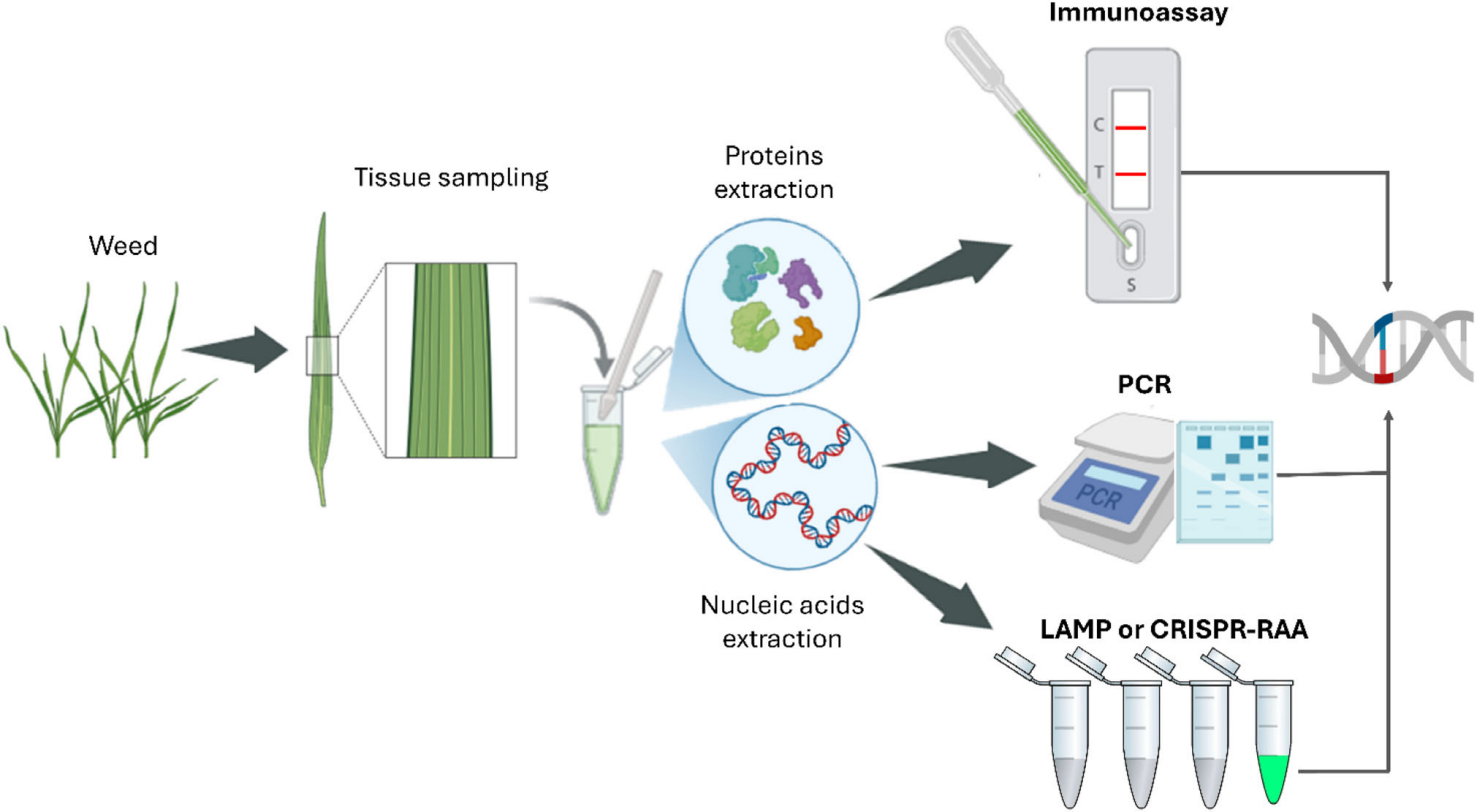
Quick diagnosis of herbicide resistance in the field or laboratory. Resistant weed tissue collected in the field could have its resistance mechanism identified using a rapid extraction method for proteins or nucleic acids, followed by an immunoassay, CRISPR‐RAA, or LAMP assay developed specifically for the most common resistance mechanisms in that species. A lateral‐flow immunoassay would display results similarly to a COVID test, whereas CRISPR‐RAA and LAMP assays would produce a color change visible to the naked eye. Alternatively, vegetative tissue could be shipped to a laboratory for resistance diagnosis using PCR (conventional or qPCR), primarily for target‐site mechanisms, with primers designed to detect the most common mutations reported for that species. These represent different methods for the rapid diagnosis of genomic alterations. Image created with BioRender.

The molecular methods used to diagnose herbicide resistance are mainly derived from cleaved amplified polymorphic sequence (dCAPS),^232^ qPCR, PCR allele competitive extension (PACE),^16^ and kompetitive allele‐specific PCR (KASP).^231^ Molecular markers for Sanger sequencing, PACE, and qPCR targeting the most common *ALS* mutations were developed for *Echinochloa crus‐galli*.^16^ The results showed that these molecular markers are an efficient method for high‐throughput characterization of *ALS* mutations, but some false‐positive or false‐negative results were observed.^16^ This limitation would potentially be solved by increasing the number of replicates when running the analysis. The Ala122Thr, Ala205Asn, and Ser653Asn showed the most consistent results when using PACE or qPCR methods compared to the actual DNA sequencing.^16^ In addition, the method has the capacity to detect one resistant plant in a bulk with several susceptible (up to one resistant mixed with 19 other susceptible plants), if the same amount of leaf tissue is collected from each plant.^16^ Molecular markers have been developed for several weed species resistant to several herbicides. KASP molecular markers were developed in *Amaranthus retroflexus* to genotype fomesafen‐resistant plants with Arg128Gly mutation in the *PPX2* gene,^231^ and in *Chloris virgata* to genotype Trp2027Cys in the *ACCase* gene.^233^ In *Lolium perenne*, dCAPS makers were successful in genotyping glyphosate‐resistant plants carrying Pro106Ser in *EPSPS* gene,^232^ and in detecting the Trp2027Cys in the *ACCase* gene of *Leptochloa chinensis* resistant to cyhalofop‐butyl.^234^

The molecular markers method described earlier is quicker than the traditional glasshouse testing, but still needs a laboratory infrastructure. New methods, such as loop‐mediated isothermal amplification (LAMP) and CRISPR‐based, could have the same advantage as dCAPS, qPCR, or KASP markers, but with the advantage of potential portability and in‐field use. LAMP is a form of DNA amplification (like PCR), but it operates at a constant temperature and can be run on a small portable device or even a heating block, without the need for a thermocycler.^235^ The LAMP protocol detected Ile1781Leu and Asp2078Gly mutations in the *ACCase* gene in fenoxaprop*‐p*‐ethyl‐resistant *Alopecurus aequalis*, enabling noticeable naked eye color change visualization when using SYBR Green I dye.^236^ This study also found that the sensitivity of the LAMP assay was approximately 10^4^‐fold greater than that of conventional PCR methods.^236^ In the *Amaranthus* species, the accuracy of the LAMP method in detecting Trp574Leu in *ALS* gene was variable, with very promising results.^237^ However, the results show that the assay was effective on crude genomic DNA extraction, an important step for in‐field use.^237^ Ideally, a LAMP‐based diagnosis in the field would take a leaf punch, grind it in a small tube with DNA extraction buffer, add reagents, keep the tube warm (60–65 °C) for an hour, then observe the color. Promising advances have been made in LAMP assays, such as LAMP‐on‐a‐chip, and paper‐based LAMP with application in clinical diagnostics and pathogen detection.^238^ The other exciting and recent area is CRISPR‐based diagnostic platforms, such as recombinase‐aided amplification (RAA) with CRISPR/Cas12a technology, which, similar to LAMP, can be configured to detect specific DNA sequences and produce a quick visual signal, potentially in field‐deployable formats.^239^ It works at a constant temperature (typically 37–42 °C), amplification can occur in 15–30 min, and the final result is a visible signal, making it ideal for field use. This isothermal DNA amplification uses a recombinase and a strand‐displacing polymerase to amplify a target DNA sequence without the need for thermal cycling. Once the target DNA is amplified, a guide‐RNA takes the Cas12a to the exact single nucleotide polymorphism (SNP) of interest in the DNA sequence, and if the target sequence is present, Cas12a binds and cleaves nearby single‐stranded DNA reporters (usually labeled with a fluorophore and quencher).^239^ This method has been developed to detect Ser59Gly mutation in *GS1* gene of glufosinate‐resistant *Eleusine indica*,^240^ Trp574Leu in *ALS* gene of ALS‐inhibitors‐resistant *Alopecurus japonicus*, *Alopecurus aequalis*, and *Alopecurus myosuroides*,^241^ Trp2027Cys in *ACCase* of *Leptochloa chinensis*,^242^ and Ile1781Val mutation in *ACCase* of *Leptochloa fusca*.^243^ The same system, but utilizing Cas12b, was developed to detect SNPs at Ile1781 position of the *ACCase* gene conferring resistance to pinoxaden in *Beckmannia syzigachne* at constant temperature of 54 °C within 40 min.^244^ As LAMP and RAA‐CRISPR/Cas12a assays improve, we might see commercial field kits for common resistance mutations.

The development of NTSR mechanisms quick tests is more complex than TSR mechanisms, as many potential candidate genes can act alone or in combination, and very often we do not know which gene is driving the resistance. The characterization of genes that metabolize herbicides has been increasing in several species (Table 3), but we need to consider that different populations of the same species may develop different NTSR mechanisms. Weed science is still advancing in better characterizing the genes that play a major role in resistance, because there is a large number of *CYP*, *GST*, and *ABC* genes in plants, which makes it difficult to identify and validate candidate genes. To illustrate, allohexaploid *Echinochloa crus‐galli* has 867 *CYP* and 227 *GST*, 361 *ABC*, and 113 *AKR* genes.^245^ Continuous investigation of metabolic resistance is critical to characterize more resistance‐causing genes, considering that different populations from the same species can evolve resistance to the same herbicide, but through different enzymes. A better understanding of metabolic genes is essential for potential quick test development to diagnose resistance, in the field or in the laboratory, but also may greatly benefit the agrochemical industry in predicting if new herbicides coming from their discovery pipeline have the potential to fail in the field even before their launch. For example, the deep understanding of *Alopecurus myosuroides* resistance allowed researchers to select the most important metabolic genes and develop markers to correlate their expression and protein production with fenoxaprop‐*p*‐ethyl and mesosulfuron‐methyl.^121^ So far, the only in‐field test developed is an immunodiagnostic quick test for NTSR, which detects elevated levels of a GST enzyme (*GSTF1*) that correlates with metabolic resistance in *Alopecurus myosuroides*. Farmers crush a small sample of *Alopecurus myosuroides* leaf in a buffer and apply a few drops to the lateral‐flow device. Like a COVID test, the GSTF1 assay provides results within 10 min, showing a control line to indicate that the test is functioning and a test line on the strip only if the GSTF1 enzyme level is high (indicating the weed likely has enhanced metabolism conferring resistance).^246^ However, the metabolic resistance assays do not inform which herbicides the population is resistant to, just that it has a certain enhanced metabolism trait. Some genes have been extensively characterized to confer resistance to multiple herbicides, such as *GSTF1* in *Alopecurus myosuroides* and *CYP81A12* in *Echinochloa phyllopogon*. However, the quick test for NTSR could also be molecular markers, if the genomic alteration that causes increased expression is discovered. Immunostrip kits would be the best quick test option to use in the field, because they are highly portable, user‐friendly, and require no laboratory and minimal training. In theory, they could be developed for other weeds and resistance markers, such as *ALS* target site mutation if an antibody can distinguish the mutated enzyme from the wild‐type. The challenge is developing antibodies for each resistance marker.

The evolution of herbicide resistance diagnostics has moved from labor‐intensive glasshouse bioassays to high‐throughput and precise molecular and biochemical tools. While TSR mechanisms are well‐characterized and amenable to rapid genotyping through PCR‐based methods and emerging field‐deployable platforms like LAMP and RAA‐CRISPR/Cas12a and Cas12b, NTSR remains a more complex challenge. Nonetheless, progress is being made, such as the immunodiagnostic tools for *GSTF1*. As our understanding of resistance mechanisms expands and more mutations/mechanisms are characterized, these field‐deployable assays will empower timely, informed herbicide decisions, reducing costs and improving weed management outcomes.

## 7 RNA INTERFERENCE‐BASED CONTROL BENEFITS FROM UNDERSTANDING THE MECHANISMS OF RESISTANCE

Post‐transcriptional regulation of gene expression through spray‐induced gene silencing (SIGS) has emerged as a promising biotechnological tool for weed management, offering a novel MoA distinct from conventional herbicides. It is feasible by utilizing tools to degrade messenger RNA (mRNA) before it is translated into a protein/enzyme. RNA interference (RNAi) involves the exogenous application of long precursor double‐stranded RNA (dsRNA) or hairpin RNA (hpRNA), which are processed into approximately 21‐nucleotide small interfering RNAs (siRNAs) acting in degrading mRNA of the desired transcript.^247^ The emergence of RNAi technology for weed control heavily relies on understanding resistance mechanisms. RNAi technologies could target resistance genes, knocking down metabolic gene expression and re‐establishing susceptibility to herbicides, or working as an ‘herbicide’ itself, knocking down essential genes.^248^ Considering the transient effect of SIGS, in case of metabolic resistance suppression, the herbicide needs to be mixed with the RNAi or sprayed in a short window of time.

Effective RNAi needs to specifically target the transcripts involved in resistance mechanisms, such as the mRNA from *CYP81A10* that metabolizes a range of herbicides in *Lolium rigidum* (or the transcription factor that regulates its expression),^9^ or the *EPSPS* wild‐type transcript in *Amaranthus palmeri* showing an increased number of gene copies in the DNA.^14^ To achieve it, a deep understanding of metabolic genes and their regulation is required in many species resistant to different herbicides. The large variability of homologous gene sequences across species and the diversity of gene families driving metabolic resistance (Table 3) are challenges for making broad‐spectrum RNAi constructs. In addition, very often more than one metabolic gene contributes to resistance, such as the *AKR4* and *ABCC8* genes in *Echinochloa colona* resistant to glyphosate,^157^, ^169^ and *CYP71A1* and *GSTU23* in *Echinochloa oryzoides* resistant to thiobencarb.^249^

It is not possible to discard that there is a high chance of weeds also evolving resistance to RNAi via SNPs, as verified for a different molecular control technique called gene drive, which targets sex genes in mosquitoes.^250^ This TSR‐type could potentially be overcome by utilizing different RNAi constructs for the same gene, combining two or more RNAi constructs, or designing RNAi based on amino acids essential for the enzyme functionality. These hypotheses still need to be tested.

The development of SIGS, other than relying on understanding the mechanism of resistance, requires genomic resources to accurately design RNAi molecules and identify conserved and essential gene targets. The International Weed Genomics Consortium and the weed science community have sequenced several high‐quality genomes of important weed species for the first time, such as *Amaranthus palmeri*,^251^ *Conyza bonariensis*, *Conyza sumatrensis*,^252^ *Conyza canadensis*,^253^ and *Echinochloa crus‐galli*.^12^ The next step are pangenomic studies to capture the genomic variability in different populations. Another layer of challenge is the polyploidy genomic architecture of several troublesome weeds. The RNAi molecule has to target the multiple homoeologous copies present in the genome, taking into account their potential sequence variability, because missing one copy would be enough for the plant's survival. This has been proven in herbicide resistance, where the mutation in one out of three *ALS* genes in allohexaploid *Echinochloa crus‐galli* is sufficient to confer survival after herbicide application.^30^ This higher gene dosage raises the question of whether it would reflect in higher RNAi dosage required for polyploid compared to diploid species.

Other than the challenges of developing a successful RNAi molecule, an efficient delivery system and low/no ecological risk are also important to develop a robust technology package. Unlike insects, where ingestion facilitates RNA uptake, plants require foliar or root uptake, which is hindered by cuticle barriers and RNA degradation by environmental factors such as ultraviolet (UV) radiation, microbial degradation, and pH fluctuations, which can significantly reduce dsRNA half‐life, limiting its efficacy in field conditions.^254^ Strategies to stabilize and protect RNA molecules using nanocarriers, such as liposomes, nanocapsules, and metallic nanoparticles, among others, have shown promise in enhancing dsRNA stability, cellular uptake, and systemic movement within plant tissues.^254^, ^255^ The variability in leaf surface morphology, presence or absence of trichomes, and cuticle composition in different weed species complicates the design of universal delivery systems and necessitates species‐specific optimization.

While researchers continue to face challenges, progress has been reported in developing efficient RNAi molecules, delivery systems, and/or formulations in weeds, such as in *Oryza sativa* f. *spontanea* ^256^ and *Mikania micrantha*.^257^ There are examples of the successful implementation of SIGS as an insecticide. Calantha™ is a commercial RNAi‐based insecticide to control *Leptinotarsa decemlineata* by targeting the essential gene *PSMB5*, and registered in the United States in 2023.^258^, ^259^ Other studies have shown effective down‐regulation of metabolic genes reflected in decreased survival of insects after insecticide spray.^260^, ^261^, ^262^ The knowledge of insecticide resistance regulation has always been in a more advanced stage when compared to herbicide resistance, which corroborates the subject of our review: the more we know about the mechanisms, the better we can combat them.

## 8 FINAL REMARKS

In this review, we have highlighted numerous ways that knowledge about herbicide resistance mechanisms in weeds can be used to develop strategies to control resistant biotypes (Table 4). The current management recommendation is herbicide MoA rotation, herbicide mixtures, and integrated weed management practices, which are effective but not always possible or practical. When possible, the adoption of non‐chemical weed control practices, such as the adoption of cover crops, crop rotation, more competitive crop cultivars/hybrids, and weed seed destruction at harvest, is always welcome, as it contributes to and diversifies the strategies used to combat herbicide resistance.

**Table 4:** Summary of the main management strategies proposed for each resistance mechanism, and the requirements for their implementation

Mechanisms	Main actions proposed	Requirements/limitations
Target site resistance		
Single mutation	Rotate herbicide mode of action (MoA) Rotate chemical family in the same mode of action when possible	**To develop** ^a^: Quick testing tools to identify the mutations in advance
	Consider the negative cross‐resistance concept in the rotation or mixtures program	**To research** ^b^: Understand the cross‐resistance pattern of mutations in different species
	Spray the herbicide in pre‐emergence instead of post‐emergence when possible.	**To research**: Knowledge of the mutations for which a pre‐emergent application is more effective than a post‐emergent application
	RNA interference (RNAi) targeting specifically the mutation in the target gene	**To develop**: Efficient delivery of RNAi molecules
Double or triple mutation	Rotate herbicide MoA RNAi targeting specifically the mutation in the target gene	**To develop**: Quick testing tools to identify the mutations in advance **To develop**: Efficient delivery of RNAi molecules
Deletion		
Target overexpression	Rotate herbicide MoA	**To develop**: Quick testing tools to characterize the number of copies in advance
	RNAi to down‐regulate the target gene expression, followed by herbicide spray	**To develop**: Efficient delivery of RNAi molecules
Non‐target site resistance		
Metabolic	Usually, spraying at lower temperatures increases the control (exception: paraquat has higher control at a higher temperature)	**To develop**: Quick testing tools to discard the presence of known target site mechanisms
	Mixtures or rotation program with herbicides not, or less, prone to being metabolized/sequestered	Accurate forecast to plan the application, aiming for low temperature after the application
	Metabolic/transporters inhibitors	**To develop**: Efficient and low‐toxic metabolic inhibitors
Vacuolar sequestration	RNAi to down‐regulate the metabolic gene expression, followed by herbicide spray	**To research**: Identification of genes driving the resistance
		**To develop**: Efficient delivery of RNAi molecules to down‐regulate the metabolic/transporter genes
Mediated by microorganisms	Mixtures with fungicides, antibiotics, or the application of antagonistic microorganisms	**To research**: Understand if and what pesticides are efficient in controlling microorganisms mediating resistance
Rapid necrosis	Rotate active ingredients (do not need to rotate MoA)	**To research**: Identify cross‐resistance patterns and environmental effects
	Avoid mixtures with the herbicide that causes necrosis	
	If the mixture includes a herbicide that causes necrosis, spray it at night (low light and temperature)	

^a^ To develop = Technology development is needed.

^b^ To research = Additional scientific studies/information are needed.

Target‐site mutations endowing herbicide resistance can be managed by rotating herbicides from different chemical groups (not just different MoAs), while non‐target‐site mutations may be managed through environmental factors, mainly temperature. To achieve this level of applicability, studies reporting herbicide resistance should improve their investigation of resistance mechanisms and the interaction with the environment and other factors that may alter resistance phenotypes. More studies concerning cross‐resistance are critical, especially for mutations that do not show a well‐defined pattern. In the same way, temperature effect investigations are also important to develop management strategies, but also to phenotypically characterize resistant populations, avoiding false negatives in lower temperatures, for instance. The pre‐emergence application may result in increased herbicide efficacy against resistant biotypes selected with the same herbicide at post‐emergence, for low‐level mechanisms of resistance. Taking into account the NCR concept and general epidemiological principles when designing an herbicide rotation program can help manage resistant populations and, if implemented in advance, may also help delay resistance evolution. The simple report of a resistant phenotype to herbicides and species already reported must be discouraged to stimulate papers with deeper investigations that could reflect in future field applications (Fig. 5).

**Figure 5:**
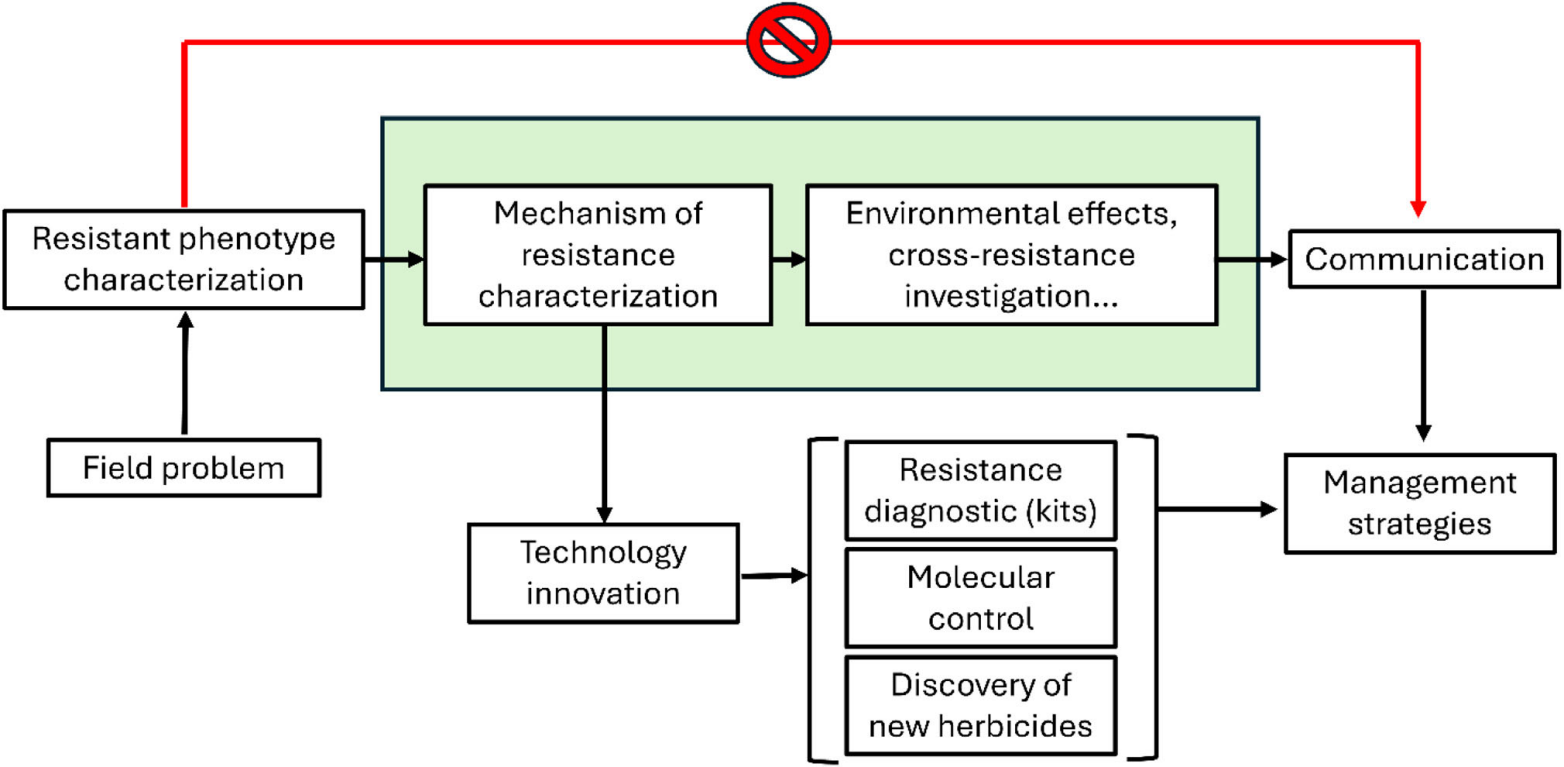
Herbicide resistance reports. Herbicide resistance reports should be encouraged to include more complete information to better support the development of management strategies and innovative technologies.

The implementation of effective management strategies according to the mechanism of resistance is still limited, mainly due to the lack of ability to rapidly characterize the exact resistance mechanisms in the field. This work requires laboratories specialized in molecular biology, biochemistry, and/or the development of quick assays. Additionally, regional‐scale herbicide resistance surveys can help infer the most prevalent resistance mechanisms in a given area and, based on that information, guide optimal use recommendations, particularly for herbicides to which resistant biotypes are present but whose use remains viable due to low cost, favorable environmental profiles, and the need to control other susceptible weed species. These adjustments are especially important given the limited availability of new herbicide MoAs.

Taken together, we are reaching a point where we have amassed a tremendous amount of information about herbicide resistance, but translating that information into practical weed control is still difficult and in its infancy. This review points out several ways this information can be translated; however, such translation will likely need to occur on a region‐by‐region, system‐by‐system, and species‐by‐species basis, as each situation appears to be unique. We believe it is essential to continue characterizing these resistance mechanisms and to strengthen future studies by incorporating assessments of cross‐resistance, fitness penalties, environmental interactions, and diagnostic tools, thereby maximizing the value of discoveries in the field of herbicide resistance.

## CONFLICTS OF INTEREST

The authors have no conflicts of interest to declare.

## FUNDING INFORMATION

This work was supported by the National Council for Scientific and Technological Development (CNPq) for fellowships to LC (141 070/2018‐8). The Article Processing Charge for the publication of this research was funded by the Coordenação de Aperfeiçoamento de Pessoal de Nível Superior – Brasil (CAPES) (ROR identifier: 00x0ma614).

## Data Availability

Data sharing not applicable to this article as no datasets were generated or analysed during the current study.

## References

[1] Gould F , Brown ZS and Kuzma J , Wicked evolution: can we address the sociobiological dilemma of pesticide resistance? Science 360:728–732 (2018). 10.1126/science.aar3780. 29773742

[2] Heap I , The International Herbicide‐Resistant Weed Database (2026). Available: www.weedscience.org.

[3] Beckie HJ and Reboud X , Selecting for weed resistance: herbicide rotation and mixture. Weed Technol 23:363–370 (2009). 10.1614/WT-09-008.1.

[4] Powles SB , Evolved glyphosate‐resistant weeds around the world: lessons to be learnt. Pest Manag Sci 64:360–365 (2008). 10.1002/ps.1525. 18273881

[5] Shaner DL , Lessons learned from the history of herbicide resistance. Weed Sci 62:427–431 (2014). 10.1614/WS-D-13-00109.1.

[6] Tranel PJ and Wright TR , Resistance of weeds to ALS‐inhibiting herbicides: what have we learned? Weed Sci 50:700–712 (2002). 10.1614/0043-1745(2002)050[0700:RROWTA]2.0.CO;2.

[7] Schroeder J , Barrett M , Shaw DR , Asmus AB , Coble H , Ervin D *et al*., Managing wicked herbicide‐resistance: lessons from the field. Weed Technol 32:475–488 (2018). 10.1017/wet.2018.49.

[8] Gaines TA , Duke SO , Morran S , Rigon CAG , Tranel PJ , Küpper A *et al*., Mechanisms of evolved herbicide resistance. J Biol Chem 295:10307–10330 (2020). 10.1074/jbc.REV120.013572. PMC7383398 32430396

[9] Han H , Yu Q , Beffa R , González S , Maiwald F , Wang J *et al*., Cytochrome P450 CYP81A10v7 in *Lolium rigidum* confers metabolic resistance to herbicides across at least five modes of action. Plant J 105:79–92 (2021). 10.1111/tpj.15040. 33098711

[10] Rigon CAG , Gaines TA , Küpper A and Dayan FE , Metabolism‐based herbicide resistance, the major threat among the non‐target site resistance mechanisms. Outlooks Pest Manag 31:162–168 (2020). 10.1564/v31_aug_04.

[11] Luo Q , Chen S , Zhu J , Ye L , Hall ND , Basak S *et al*., Overexpression of EiKCS confers paraquat‐resistance in rice (*Oryza sativa* L.) by promoting the polyamine pathway. Pest Manag Sci 78:246–262 (2022). 10.1002/ps.6628. PMC9292836 34476895

[12] Guo L , Qiu J , Ye C , Jin G , Mao L , Zhang H *et al*., *Echinochloa crus‐galli* genome analysis provides insight into its adaptation and invasiveness as a weed. Nat Commun 8:1031 (2017). 10.1038/s41467-017-01067-5. PMC5647321 29044108

[13] Montgomery J , Morran S , MacGregor DR , McElroy JS , Neve P , Neto C *et al*., Current status of community resources and priorities for weed genomics research. Genome Biol 25:139 (2024). 10.1186/s13059-024-03274-y. PMC11129445 38802856

[14] Gaines TA , Zhang W , Wang D , Bukun B , Chisholm ST , Shaner DL *et al*., Gene amplification confers glyphosate resistance in *Amaranthus palmeri* . Proc Natl Acad Sci USA 107:1029–1034 (2010). 10.1073/pnas.0906649107. PMC2824275 20018685

[15] Gaines TA , Barker AL , Patterson EL , Westra P , Westra EP , Wilson RG *et al*., *EPSPS* gene copy number and whole‐plant glyphosate resistance Level in *Kochia scoparia* . PLoS One 11:e0168295 (2016). 10.1371/journal.pone.0168295. PMC5161467 27992501

[16] Rudell EC , Cutti L , Turra GM , Angonese PS , Tasca VF , Dos Santos OD *et al*., Variability and spatial distribution of ALS‐inhibitor resistance mechanisms in Brazilian *Echinochloa crus‐galli* . Plant Physiol Biochem 228:110237 (2025). 10.1016/j.plaphy.2025.110237. 40684739

[17] Sulzback JRM , Borgato EA , Cutti L , Hill EC , Burns EE and Patterson EL , Optimizing molecular assays for glyphosate and ALS‐inhibitor resistance diagnostics in four weed species. Can J Plant Sci 104:540–554 (2024). 10.1139/cjps-2024-0020.

[18] Zhou Q , Liu W , Zhang Y and Liu KK , Action mechanisms of acetolactate synthase‐inhibiting herbicides. Pestic Biochem Physiol 89:89–96 (2007). 10.1016/j.pestbp.2007.04.004.

[19] Gherekhloo J , Hatami ZM , Alcántara‐de la Cruz R , Sadeghipour HR and De Prado R , Continuous use of Tribenuron‐methyl selected for cross‐resistance to Acetolactate synthase–inhibiting herbicides in wild mustard (*Sinapis arvensis*). Weed Sci 66:424–432 (2018). 10.1017/wsc.2018.23.

[20] Singh S , Singh V , Salas‐Perez RA , Bagavathiannan MV , Lawton‐Rauh A and Roma‐Burgos N , Target‐site mutation accumulation among ALS inhibitor‐resistant palmer amaranth. Pest Manag Sci 75:1131–1139 (2019). 10.1002/ps.5232. 30298618

[21] Deng W , Di Y , Cai J , Chen Y and Yuan S , Target‐site resistance mechanisms to Tribenuron‐methyl and cross‐resistance patterns to ALS‐inhibiting herbicides of Catchweed bedstraw (*Galium aparine*) with different ALS mutations. Weed Sci 67:183–188 (2019). 10.1017/wsc.2018.70.

[22] Porri A , Panozzo S , Sisay MT , Scarabel L , Lerchl J and Milani A , 3D structure of acetolactate synthase explains why the Asp‐376‐Glu point mutation does not give the same resistance level to different imidazolinone herbicides. Pestic Biochem Physiol 204:106070 (2024). 10.1016/j.pestbp.2024.106070. 39277385

[23] Li J , Gao X , Li M and Fang F , Resistance evolution and mechanisms to ALS‐inhibiting herbicides in *Capsella bursa‐pastoris* populations from China. Pestic Biochem Physiol 159:17–21 (2019). 10.1016/j.pestbp.2019.05.010. 31400779

[24] Li J , Li M , Gao X and Fang F , A novel amino acid substitution Trp574Arg in acetolactate synthase (ALS) confers broad resistance to ALS‐inhibiting herbicides in crabgrass (*Digitaria sanguinalis*). Pest Manag Sci 73:2538–2543 (2017). 10.1002/ps.4651. 28643897

[25] Hamouzová K , Košnarová P , Salava J , Soukup J and Hamouz P , Mechanisms of resistance to acetolactate synthase‐inhibiting herbicides in populations of *Apera spica‐venti* from The Czech Republic. Pest Manag Sci 70:541–548 (2014). 10.1002/ps.3563. 23893862

[26] Zheng D , Kruger GR , Singh S , Davis VM , Tranel PJ , Weller SC *et al*., Cross‐resistance of horseweed (*Conyza canadensis*) populations with three different ALS mutations. Pest Manag Sci 67:1486–1492 (2011). 10.1002/ps.2190. 21538801

[27] Deng W , Yang Q , Zhang Y , Jiao H , Mei Y , Li X *et al*., Cross‐resistance patterns to acetolactate synthase (ALS)‐inhibiting herbicides of flixweed (*Descurainia sophia* L.) conferred by different combinations of ALS isozymes with a Pro‐197‐Thr mutation or a novel Trp‐574‐Leu mutation. Pestic Biochem Physiol 136:41–45 (2017). 10.1016/j.pestbp.2016.08.006. 28187829

[28] Kaspary TE , Cutti L , Turra GM , Angonese PS , Dos Santos OD and Merotto A Jr , *Conyza bonariensis* resistance to glyphosate and ALS inhibitors involves target and non‐target site resistance. Pestic Biochem Physiol 213:106501 (2025). 10.1016/j.pestbp.2025.106501. 40744617

[29] Yu Q , Han H and Powles SB , Mutations of the ALS gene endowing resistance to ALS‐inhibiting herbicides in *Lolium rigidum* populations. Pest Manag Sci 64:1229–1236 (2008). 10.1002/ps.1624. 18636424

[30] Cutti L , Turra GM , Machado FM , Sulzbach E , Angonese PS , Markus C *et al*., Subgenome dominance of ALS target site mutations impacts herbicide resistance in allohexaploid *Echinochloa crus‐galli* . Plant Direct 10:e70168 (2026). 10.1002/pld3.70168. PMC13099262 42022335

[31] Poston DH , Wilson HP and Hines TE , Imidazolinone resistance in several *Amaranthus hybridus* populations. Weed Sci 48:508–513 (2000). 10.1614/0043-1745(2000)048[0508:IRISAH]2.0.CO;2.

[32] Whaley CM , Wilson HP and Westwood JH , ALS resistance in several smooth pigweed (*Amaranthus hybridus*) biotypes. Weed Sci 54:828–832 (2006). 10.1614/WS-05-040R.1.

[33] Panozzo S , Scarabel L , Rosan V and Sattin M , A new ala‐122‐Asn amino acid change confers decreased fitness to ALS‐resistant *Echinochloa crus‐galli* . Front Plant Sci 8:2042 (2017). 10.3389/fpls.2017.02042. PMC5712356 29234345

[34] Han H , Yu Q , Purba E , Li M , Walsh M , Friesen S *et al*., A novel amino acid substitution ala‐122‐Tyr in ALS confers high‐level and broad resistance across ALS‐inhibiting herbicides. Pest Manag Sci 68:1164–1170 (2012). 10.1002/ps.3278. 22431132

[35] Kumar V and Jha P , First report of Ser653Asn mutation endowing high‐level resistance to imazamox in downy brome (*Bromus tectorum* L.). Pest Manag Sci 73:2585–2591 (2017). 10.1002/ps.4673. 28734097

[36] Laplante J , Rajcan I and Tardif FJ , Multiple allelic forms of acetohydroxyacid synthase are responsible for herbicide resistance in *Setaria viridis* . Theor Appl Genet 119:577–585 (2009). 10.1007/s00122-009-1067-5. 19495723

[37] Turra GM , Cutti L , Machado FM , Dias GM , Andres A , Markus C *et al*., Application of ALS inhibitors at pre‐emergence is effective in controlling resistant barnyardgrass biotypes depending on the mechanism of resistance. Crop Prot 172:106325 (2023). 10.1016/j.cropro.2023.106325.

[38] Torra J , Mora G , Montull JM , Royo‐Esnal A , Notter JS and Salas M , A 4‐year field study monitoring the evolution of Trp574Leu‐resistant plants in an Echinochloa crus‐galli population under different crop rotation and herbicide programs in maize. Pest Manag Sci 80:5843–5851 (2024). 10.1002/ps.8315Digital Object Identifier. 39007446

[39] Kukorelli G , Reisinger P and Pinke G , ACCase inhibitor herbicides–selectivity, weed resistance and fitness cost: a review. Int J Pest Manag 59:165–173 (2013). 10.1080/09670874.2013.821212.

[40] Scutt JN , Willetts NJ , Fernandes Campos B , Oliver S , Hennessy A , Joyce PM *et al*., Metproxybicyclone, a novel carbocyclic aryl‐dione acetyl‐CoA carboxylase‐inhibiting herbicide for the management of sensitive and resistant grass weeds. J Agric Food Chem 72:21380–21392 (2024). 10.1021/acs.jafc.4c02729. 39311764

[41] Ghanizadeh H , Buddenhagen CE , Harrington KC , Griffiths AG and Ngow Z , Pinoxaden resistance in Lolium perenne L. is due to both target‐site and non‐target‐site mechanisms. Pestic Biochem Physiol 184:105103 (2022). 10.1016/j.pestbp.2022.105103. 35715042

[42] Amaro‐Blanco I , Romano Y , Palmerin JA , Gordo R , Palma‐Bautista C , de Prado R *et al*., Different mutations providing target site resistance to ALS‐ and ACCase‐inhibiting herbicides in *Echinochloa* spp. from Rice fields. Agri 11:382 (2021). 10.3390/agriculture11050382.

[43] Lan Y , Li W , Wei S , Huang H , Liu Z and Huang Z , Multiple resistance to ACCase‐ and ALS‐inhibiting herbicides in black‐grass (*Alopecurus myosuroides* Huds.) in China. Pestic Biochem Physiol 184:105127 (2022). 10.1016/j.pestbp.2022.105127. 35715065

[44] Ghanizadeh H , Mesarich CH and Harrington KC , Molecular characteristics of the first case of haloxyfop‐resistant *Poa annua* . Sci Rep 10:4231 (2020). 10.1038/s41598-020-61104-0. PMC7060245 32144361

[45] Osuna MD , Goulart ICGR , Vidal RA , Kalsing A , Ruiz Santaella JP and de Prado R , Resistance to ACCase inhibitors in *Eleusine indica* from Brazil involves a target site mutation. Planta Daninha 30:675–681 (2012). 10.1590/S0100-83582012000300025.

[46] Yang Q , Zhu J , Yang X , Wei T , Lv M and Li Y , Ile‐1781‐Leu target mutation and non‐target‐site mechanism confer resistance to acetyl‐CoA carboxylase‐inhibiting herbicides in *Digitaria ciliaris* var. *chrysoblephara* . J Agric Food Chem 71:7988–7995 (2023). 10.1021/acs.jafc.3c00646. 37191622

[47] Beckie HJ , Warwick SI and Sauder CA , Basis for herbicide resistance in Canadian populations of wild oat (*Avena fatua*). Weed Sci 60:10–18 (2012). 10.1614/WS-D-11-00110.1.

[48] Yu Q , Collavo A , Zheng MQ , Owen M , Sattin M and Powles SB , Diversity of acetyl‐coenzyme A carboxylase mutations in resistant *Lolium* populations: evaluation using clethodim. Plant Physiol 145:547–558 (2007). 10.1104/pp.107.105262. PMC2048730 17720757

[49] Kaundun SS , Hutchings SJ , Dale RP and McIndoe E , Role of a novel I1781T mutation and other mechanisms in conferring resistance to acetyl‐CoA carboxylase inhibiting herbicides in a black‐grass population. PLoS One 8:e69568 (2013). 10.1371/journal.pone.0069568. PMC3723891 23936046

[50] Délye C , Matéjicek A and Michel S , Cross‐resistance patterns to ACCase‐inhibiting herbicides conferred by mutant ACCase isoforms in *Alopecurus myosuroides* Huds. (black‐grass), re‐examined at the recommended herbicide field rate. Pest Manag Sci 64:1179–1186 (2008). 10.1002/ps.1614. 18537107

[51] Deng W , Cai J , Zhang J , Chen Y , Chen Y , Di Y *et al*., Molecular basis of resistance to ACCase‐inhibiting herbicide cyhalofop‐butyl in Chinese sprangletop (*Leptochloa chinensis* (L.) Nees) from China. Pestic Biochem Physiol 158:143–148 (2019). 10.1016/j.pestbp.2019.05.004. 31378350

[52] Liu W , Harrison DK , Chalupska D , Gornicki P , O'donnell CC , Adkins SW *et al*., Single‐site mutations in the carboxyltransferase domain of plastid acetyl‐CoA carboxylase confer resistance to grass‐specific herbicides. Proc Natl Acad Sci USA 104:3627–3632 (2007). 10.1073/pnas.0611572104. PMC1802000 17360693

[53] Xu H , Li J , Zhang D , Cheng Y , Jiang Y and Dong L , Mutations at codon position 1999 of acetyl‐CoA carboxylase confer resistance to ACCase‐inhibiting herbicides in Japanese foxtail (*Alopecurus japonicus*). Pest Manag Sci 70:1894–1901 (2014). 10.1002/ps.3753. 24497328

[54] Kaundun SS , Bailly GC , Dale RP , Hutchings SJ and McIndoe E , A novel W1999S mutation and non‐target site resistance impact on acetyl‐CoA carboxylase inhibiting herbicides to varying degrees in a UK *Lolium multiflorum* population. PLoS One 8:e58012 (2013). 10.1371/journal.pone.0058012. PMC3585232 23469130

[55] Yang Q , Deng W , Liu L , Wei T , Yang X , Zhu J *et al*., Resistance patterns and molecular basis to ACCase‐inhibiting herbicides. Weed Sci 72:352–359 (2024). 10.1017/wsc.2024.30.

[56] Ribeiro VHV , Brunharo CA , Mallory‐Smith C , Walenta DL and Barroso J , First report of target‐site resistance to ACCase‐inhibiting herbicides in *Bromus tectorum* L. Pest Manag Sci 79:4025–4033 (2023). 10.1002/ps.7607. 37309712

[57] Moisinho IS , Ferreira LAI , Krzyzaniak F , Albrecht AJP , Albrecht LP and Brunharo C , Multiple‐resistance evolution to ACCase inhibitors and glyphosate in sourgrass (*Digitaria insularis*) is attributed to diverse polymorphisms in the herbicide target sites. Weed Sci 72:517–526 (2024). 10.1017/wsc.2024.53.

[58] Mo B , Chen W , He S , Liu H , Bai L and Pan L , First Asp‐2078‐Gly mutation conferring resistance to different ACCase inhibitors in a *Polypogon fugax* population from China. Int J Mol Sci 24:528 (2022). 10.3390/ijms24010528. PMC9820770 36613971

[59] Fang J , He Z , Liu T , Li J and Dong L , A novel mutation Asp‐2078‐Glu in ACCase confers resistance to ACCase herbicides in barnyardgrass (*Echinochloa crus‐galli*). Pestic Biochem Physiol 168:104634 (2020). 10.1016/j.pestbp.2020. 32711768

[60] Kaundun SS , Hutchings SJ , Dale RP and McIndoe E , Broad resistance to ACCase inhibiting herbicides in a ryegrass population is due only to a cysteine to arginine mutation in the target enzyme. PLoS One 7:e39759 (2012). 10.1371/journal.pone.0039759. PMC3387263 22768118

[61] Cruz‐Hipolito H , Domínguez‐Valenzuela JA , Osuna MD and De Prado R , Resistance mechanism to acetyl coenzyme A carboxylase inhibiting herbicides in Phalaris paradoxa collected in Mexican wheat fields. Plant Soil 355:121–130 (2012). 10.1007/s11104-011-1085-3.

[62] Barker AL , Pawlak J , Duke SO , Beffa R , Tranel PJ , Wuerffel J *et al*., Discovery, mode of action, resistance mechanisms, and plan of action for sustainable use of group 14 herbicides. Weed Sci 71:173–188 (2023). 10.1017/wsc.2023.15.

[63] Patzoldt WL , Hager AG , McCormick JS and Tranel PJ , A codon deletion confers resistance to herbicides inhibiting protoporphyrinogen oxidase. Proc Natl Acad Sci USA 103:12329–12334 (2006). 10.1073/pnas.0603137103. PMC1567880 16894159

[64] Carvalho‐Moore P , Rangani G , Heiser J , Findley D , Bowe SJ and Roma‐Burgos N , *PPO2* mutations in *Amaranthus palmeri*: implications on cross‐resistance. Agri 11:760 (2021). 10.3390/agriculture11080760.

[65] Rangani G , Salas‐Perez RA , Aponte RA , Knapp M , Craig IR , Mietzner T *et al*., A novel single‐site mutation in the catalytic domain of Protoporphyrinogen oxidase IX (PPO) confers resistance to PPO‐inhibiting herbicides. Front Plant Sci 10:568 (2019). 10.3389/fpls.2019.00568. PMC6530635 31156659

[66] Mendes RR , Takano HK , Adegas FS , Oliveira RS , Gaines TA and Dayan FE , Arg‐128‐Leu target‐site mutation in PPO2 evolves in wild poinsettia (*Euphorbia heterophylla*) with cross‐resistance to PPO‐inhibiting herbicides. Weed Sci 68:437–444 (2020). 10.1017/wsc.2020.42.

[67] Rousonelos SL , Lee RM , Moreira MS , VanGessel MJ and Tranel PJ , Characterization of a common ragweed (*Ambrosia artemisiifolia*) population resistant to ALS‐ and PPO‐inhibiting herbicides. Weed Sci 60:335–344 (2012). 10.1614/WS-D-11-00152.1.

[68] Giacomini DA , Umphres AM , Nie H , Mueller TC , Steckel LE , Young BG *et al*., Two new PPX2 mutations associated with resistance to PPO‐inhibiting herbicides in *Amaranthus palmeri* . Pest Manag Sci 73:1559–1563 (2017). 10.1002/ps.4581. 28370968

[69] Nie H , Harre NT and Young BG , A new V361A mutation in *Amaranthus palmeri PPX2* associated with PPO‐inhibiting herbicide resistance. Plants (Basel) 12:1886 (2023). 10.3390/plants12091886. PMC10181388 37176944

[70] Bi B , Wang Q , Coleman JJ , Porri A , Peppers JM , Patel JD *et al*., A novel mutation A212T in chloroplast Protoporphyrinogen oxidase (*PPO1*) confers resistance to PPO inhibitor Oxadiazon in *Eleusine indica* . Pest Manag Sci 76:1786–1794 (2020). 10.1002/ps.5703. 31788953

[71] Dayan FE , Barker A and Tranel PJ , Origins and structure of chloroplastic and mitochondrial plant protoporphyrinogen oxidases: implications for the evolution of herbicide resistance. Pest Manag Sci 74:2226–2234 (2018). 10.1002/ps.4744. 28967179

[72] Wu C , Goldsmith M‐R , Pawlak J , Feng P , Smith S , Navarro S *et al*., Differences in efficacy, resistance mechanism and target protein interaction between two PPO inhibitors in palmer amaranth (*Amaranthus palmeri*). Weed Sci 68:105–115 (2020). 10.1017/wsc.2020.4.

[73] Sada Y , Jin Y , Hikosaka M and Ido K , Epyrifenacil, a new systemic PPO‐inhibiting herbicide for broad‐spectrum weed control. Pest Manag Sci 81:2463–2468 (2025). 10.1002/ps.8244. PMC11981982 38873996

[74] Steppig NR , Johnson WG and Young BG , Common target‐site resistance mutations for PPO‐inhibiting herbicides in waterhemp (*Amaranthus tuberculatus*) and palmer amaranth (*Amaranthus palmeri*) do not confer cross‐resistance to trifludimoxazin. Weed Sci 73:e30 (2025). 10.1017/wsc.2024.103.

[75] Todd OE , Figueiredo MRA , Morran S , Soni N , Preston C , Kubeš MF *et al*., Synthetic auxin herbicides: finding the lock and key to weed resistance. Plant Sci 300:110631 (2020). 10.1016/j.plantsci.2020.110631. 33180710

[76] LeClere S , Wu C , Westra P and Sammons RD , Cross‐resistance to dicamba, 2,4‐D, and fluroxypyr in *Kochia scoparia* is endowed by a mutation in an AUX/IAA gene. Proc Natl Acad Sci USA 115:E2911–E2920 (2018). 10.1073/pnas.1712372115. PMC5879655 29531066

[77] Ghanizadeh H , He L , Griffiths AG , Harrington KC , Carbone V , Wu H *et al*., A novel mutation in IAA16 is associated with dicamba resistance in *Chenopodium album* . Pest Manag Sci 80:3675–3683 (2024). 10.1002/ps.8071. 38459963

[78] Montgomery JS , Soni N , Marques Hill S , Morran S , Patterson EL , Edwards SA *et al*., A transposable element insertion in AUX/IAA16 disrupts splicing and causes auxin resistance in *Bassia scoparia* . Plant J 123:e70339 (2025). 10.1111/tpj.70339. PMC12277486 40685552

[79] Figueiredo MRA , Küpper A , Malone JM , Petrovic T , Figueiredo ABTB , Campagnola G *et al*., An in‐frame deletion mutation in the degron tail of auxin coreceptor *IAA2* confers resistance to the herbicide 2,4‐D in *Sisymbrium orientale* . Proc Natl Acad Sci USA 119:e2105819119 (2022). 10.1073/pnas.2105819119. PMC8892348 35217601

[80] Qi Y , Krishnan M , Tucker M and Preston C , A point mutation in *IAA34* confers resistance to the auxin herbicide 2,4‐D in *Sisymbrium orientale* . Pest Manag Sci 81:3523–3531 (2025). 10.1002/ps.8720. PMC12159386 39956931

[81] Krishnan M , Petrovic T , Schwerdt JG , Merriam AB , Hereward JP and Preston C , A novel mutation in *SoIAA20* confers cross‐resistance to 2,4‐dichlorophenoxyacetic acid and other auxinic herbicides in *Sonchus oleraceus* . Pest Manag Sci 81:141–148 (2025). 10.1002/ps.8413. PMC11632205 39268911

[82] Wang Y , Wang D , Dong B , Hao J , Jia X and Zhou H , Potential candidate molecule of photosystem II inhibitor herbicide‐Brassicanate A sulfoxide. Int J Mol Sci 25:2400 (2024). 10.3390/ijms25042400. PMC10889811 38397082

[83] Cseh A , Cernak I and Taller J , Molecular characterization of atrazine resistance in common ragweed (*Ambrosia artemisiifolia* L.). J Appl Genet 50:321–327 (2009). 10.1007/BF03195690. 19875882

[84] Thiel H and Varrelmann M , Identification of a new PSII target site psbA mutation leading to D1 amino acid Leu218 Val exchange in the *Chenopodium album* D1 protein and comparison to cross‐resistance profiles of known modifications at positions 251 and 264. Pest Manag Sci 70:278–285 (2014). 10.1002/ps.3556. 23576399

[85] Lu H , Yu Q , Han H , Owen MJ and Powles SB , Metribuzin resistance in a wild radish (*Raphanus raphanistrum*) population via both *psbA* gene mutation and enhanced metabolism. J Agric Food Chem 67:1353–1359 (2019). 10.1021/acs.jafc.8b05974. 30640451

[86] Perry DH , McElroy JS , Dane F , van Santen E and Walker RH , Triazine‐resistant annual bluegrass (*Poa annua*) populations with Ser264 mutation are resistant to Amicarbazone. Weed Sci 60:355–359 (2012). 10.1614/WS-D-11-00200.1.

[87] Mikulka J , Sen MK , Košnarová P , Hamouz P , Hamouzová K , Sur VP *et al*., Molecular mechanisms of resistance against PSII‐inhibiting herbicides in *Amaranthus retroflexus* from The Czech Republic. Genes (Basel) 15:904 (2024). 10.3390/genes15070904. PMC11275581 39062683

[88] McKenzie‐Gopsill A , Graham G , Laforest M , Ibarra S , Hann S and Wagg C , Occurrence and management of PSII‐inhibitor‐resistant *Chenopodium album* L. in Atlantic Canadian potato production. Agronomy 10:1369 (2020). 10.3390/agronomy10091369.

[89] Lu H , Yu Q , Han H , Owen MJ and Powles SB , A novel psbA mutation (Phe274‐Val) confers resistance to PSII herbicides in wild radish (*Raphanus raphanistrum*). Pest Manag Sci 75:144–151 (2019). 10.1002/ps.5079. 29797480

[90] Dumont M , Letarte J and Tardif FJ , Identification of a psbA mutation (Valine219 to isoleucine) in Powell Amaranth (*Amaranthus powellii*) conferring resistance to Linuron. Weed Sci 64:6–11 (2016). 10.1614/WS-D-15-00087.1.

[91] Mengistu LW , Mueller‐Warrant GW , Liston A and Barker RE , psbA mutation (valine219 to isoleucine) in *Poa annua* resistant to metribuzin and diuron. Pest Manag Sci 56:209–217 (2000). 10.1002/(SICI)1526-4998(200003)56:3<209::AID-PS117>3.0.CO;2-8.

[92] Pedroso RM , Al‐Khatib K , Alarcón‐Reverte R and Fischer AJ , A psbA mutation (Val219 to Ile) causes resistance to propanil and increased susceptibility to bentazon in *Cyperus difformis* . Pest Manag Sci 72:1673–1680 (2016). 10.1002/ps.4267. 26929096

[93] Perez‐Jones A , Intanon S and Mallory‐Smith C , Molecular analysis of Hexazinone‐resistant Shepherd's‐purse (*Capsella bursa‐pastoris*) reveals a novel *psbA* mutation. Weed Sci 57:574–578 (2009). 10.1614/WS-09-089.1.

[94] Laforest M , Soufiane B , Bisaillon K , Bessette M , Page ER and White SN , The amino acid substitution Phe‐255‐Ile in the *psbA* gene confers resistance to hexazinone in hair fescue (*Festuca filiformis*) plants from lowbush blueberry fields. Weed Sci 70:401–407 (2022). 10.1017/wsc.2022.36.

[95] Park KW and Mallory‐Smith CA , *psbA* mutation (Asn266 to Thr) in *Senecio vulgaris* L. confers resistance to several PS II‐inhibiting herbicides. Pest Manag Sci 62:880–885 (2006). 10.1002/ps.1252. 16791906

[96] Morejohn LC , Bureau TE , Molè‐Bajer J , Bajer AS and Fosket DE , Oryzalin, a dinitroaniline herbicide, binds to plant tubulin and inhibits microtubule polymerization in vitro. Planta 172:252–264 (1987). 10.1007/BF00394595. 24225878

[97] Chen J , Chu Z , Han H , Goggin DE , Yu Q , Sayer C *et al*., A Val‐202‐Phe α‐tubulin mutation and enhanced metabolism confer dinitroaniline resistance in a single *Lolium rigidum* population. Pest Manag Sci 76:645–652 (2020). 10.1002/ps.5561. 31329340

[98] Chu Z , Chen J , Nyporko A , Han H , Yu Q and Powles S , Novel α‐tubulin mutations conferring resistance to Dinitroaniline herbicides in *Lolium rigidum* . Front Plant Sci 9:97 (2018). 10.3389/fpls.2018.00097. PMC5810296 29472938

[99] Yamamoto E , Zeng L and Baird WV , Alpha‐tubulin missense mutations correlate with antimicrotubule drug resistance in *Eleusine indica* . Plant Cell 10:297–308 (1998). 10.1105/tpc.10.2.297. PMC143984 9490751

[100] Russell EC , Peppers JM , Rutland CA , Patel J , Hall ND , Gamble AV *et al*., Mitotic‐inhibiting herbicide response variation in goosegrass (*Eleusine indica*) with a Leu‐136‐Phe substitution in α‐tubulin. Weed Sci 70:20–25 (2022). 10.1017/wsc.2021.65.

[101] Russell EC , Rutland CA , Patel J , Hall ND , Bi B , Li X *et al*., α‐Tubulin mutation Thr‐239‐Ile in annual bluegrass (*Poa annua*) induces variable responses to prodiamine and dithiopyr. Weed Sci 71:438–443 (2023). 10.1017/wsc.2023.39.

[102] Délye C , Menchari Y , Michel S and Darmency H , Molecular bases for sensitivity to tubulin‐binding herbicides in green foxtail. Plant Physiol 136:3920–3932 (2004). 10.1104/pp.103.037432. PMC535825 15531712

[103] Chen J , Lu H , Han H , Yu Q , Sayer C and Powles S , Genetic inheritance of dinitroaniline resistance in an annual ryegrass population. Plant Sci 283:189–194 (2019). 10.1016/j.plantsci.2019.02.019. 31128688

[104] Sammons RD and Gaines TA , Glyphosate resistance: state of knowledge. Pest Manag Sci 70:1367–1377 (2014). 10.1002/ps.3743. PMC4260172 25180399

[105] Schönbrunn E , Eschenburg S , Shuttleworth WA , Schloss JV , Amrhein N , Evans JN *et al*., Interaction of the herbicide glyphosate with its target enzyme 5‐enolpyruvylshikimate 3‐phosphate synthase in atomic detail. Proc Natl Acad Sci USA 98:1376–1380 (2001). 10.1073/pnas.98.4.1376. PMC29264 11171958

[106] Huffman JL , Riggins CW , Steckel LE and Tranel PJ , The EPSPS Pro106Ser substitution solely accounts for glyphosate resistance in a goosegrass (*Eleusine indica*) population from Tennessee, United States. J Integr Agric 15:1304–1312 (2016). 10.1016/S2095-3119(15)61220-5.

[107] Chen J , Cui H , Li Z , Yu H , Hou Q and Li X , Potential role of EPSPS mutations in the resistance of *Eleusine indica* to glyphosate. Int J Mol Sci 24:8250 (2023). 10.3390/ijms24098250. PMC10179075 37175957

[108] Takano HK , Fernandes VN , Adegas FS , Oliveira RS Jr , Westra P , Gaines TA *et al*., A novel TIPT double mutation in EPSPS conferring glyphosate resistance in tetraploid *Bidens subalternans* . Pest Manag Sci 76:95–102 (2020). 10.1002/ps.5535. 31251461

[109] Sulzbach E , Turra GM , Cutti L , Kroth LVE , Tranel PJ , Merotto A Jr *et al*., Smooth pigweed (*Amaranthus hybridus*) and unresolved Amaranthus spp. from Brazil resistant to glyphosate exhibit the EPSPS TAP‐IVS substitution. Weed Sci 72:48–58 (2024). 10.1017/wsc.2023.70.

[110] Logusch EW , Walker DM , McDonald JF and Franz JE , Inhibition of plant glutamine synthetases by substituted phosphinothricins. Plant Physiol 95:1057–1062 (1991). 10.1104/pp.95.4.1057. PMC1077651 16668090

[111] Zhang C , Yu Q , Han H , Yu C , Nyporko A , Tian X *et al*., A naturally evolved mutation (Ser59Gly) in glutamine synthetase confers glufosinate resistance in plants. J Exp Bot 73:2251–2262 (2022). 10.1093/jxb/erac008. PMC8982429 35029685

[112] Avila‐Garcia WV , Sanchez‐Olguin E , Hulting AG and Mallory‐Smith C , Target‐site mutation associated with glufosinate resistance in Italian ryegrass (*Lolium perenne* L. ssp. *multiflorum*). Pest Manag Sci 68:1248–1254 (2012). 10.1002/ps.3286. 22488875

[113] Kaloumenos NS , Capote N , Aguado A and Eleftherohorinos IG , Red rice (*Oryza sativa*) cross‐resistance to imidazolinone herbicides used in resistant rice cultivars grown in northern Greece. Pestic Biochem Physiol 105:177–183 (2013). 10.1016/j.pestbp.2013.01.008.

[114] Yu Q , Ahmad‐Hamdani MS , Han H , Christoffers MJ and Powles SB , Herbicide resistance‐endowing *ACCase* gene mutations in hexaploid wild oat (*Avena fatua*): insights into resistance evolution in a hexaploid species. Heredity 110:220–231 (2013). 10.1038/hdy.2012.69. PMC3668648 23047200

[115] Han H , Yu Q , Widderick MJ and Powles SB , Target‐site EPSPS Pro‐106 mutations: sufficient to endow glyphosate resistance in polyploid *Echinochloa colona*? Pest Manag Sci 72:264–271 (2016). 10.1002/ps.4038. 25969123

[116] Alarcón‐Reverte R , García A , Urzúa J and Fischer AJ , Resistance to glyphosate in junglerice (*Echinochloa colona*) from California. Weed Sci 61:48–54 (2013). 10.1614/WS-D-12-00073.1.

[117] Simarmata M and Penner D , The basis for glyphosate resistance in rigid ryegrass (*Lolium rigidum*) from California. Weed Sci 56:181–188 (2008). 10.1614/WS-07-057.1.

[118] Beres ZT , Giese LA , Mackey DM , Owen MD , Page ER and Snow AA , Target‐site *EPSPS* Pro‐106‐Ser mutation in *Conyza canadensis* biotypes with extreme resistance to glyphosate in Ohio and Iowa, USA. Sci Rep 10:7577 (2020). 10.1038/s41598-020-64458-7. PMC7200745 32371909

[119] Ostlie M , Haley SD , Anderson V , Shaner D , Manmathan H , Beil C *et al*., Development and characterization of mutant winter wheat (*Triticum aestivum* L.) accessions resistant to the herbicide quizalofop. Theor Appl Genet 128:343–351 (2015). 10.1007/s00122-014-2434-4. 25432092

[120] Merriam AB , Malone JM , Hereward JP , Gill G and Preston C , Population structure of ALS‐inhibiting herbicide‐resistant *Sonchus oleraceus* in South Australia. Pest Manag Sci 79:3200–3210 (2023). 10.1002/ps.7498. 37029476

[121] Lowe C , Onkokesung N , Goldberg A , Beffa R , Neve P , Edwards R *et al*., RNA and protein biomarkers for detecting enhanced metabolic resistance to herbicides mesosulfuron‐methyl and fenoxaprop‐ethyl in black‐grass (*Alopecurus myosuroides*). Pest Manag Sci 80:2539–2551 (2024). 10.1002/ps.7960. 38375975

[122] Vila‐Aiub MM , Yu Q and Powles SB , Do plants pay a fitness cost to be resistant to glyphosate? New Phytol 223:532–547 (2019). 10.1111/nph.15733. 30737790

[123] Han H , Vila‐Aiub MM , Jalaludin A , Yu Q and Powles SB , A double EPSPS gene mutation endowing glyphosate resistance shows a remarkably high resistance cost. Plant Cell Environ 40:3031–3042 (2017). 10.1111/pce.13067. 28910491

[124] Barroso AAM , Michelon TB , da Costa Aguiar Alves PL , Han H , Yu Q , Powles SB *et al*., Challenging glyphosate resistance EPSPS P106S and TIPS mutations with soybean competition and glyphosate: implications for management. Pest Manag Sci 78:4764–4773 (2022). 10.1002/ps.7096. 35904507

[125] Wang Y , Han H , Chen J , Yu Q , Vila‐Aiub M , Beckie HJ *et al*., A dinitroaniline herbicide resistance mutation can be nearly lethal to plants. Pest Manag Sci 78:1547–1554 (2022). 10.1002/ps.6773. 34981627

[126] Du L , Qu M , Jiang X , Li X , Ju Q , Lu X *et al*., Fitness costs associated with acetyl‐coenzyme A carboxylase mutations endowing herbicide resistance in American sloughgrass (*Beckmannia syzigachne* Steud.). Ecol Evol 9:2220–2230 (2019). 10.1002/ece3.4917. PMC6392401 30847106

[127] Vila‐Aiub MM , Yu Q , Han H and Powles SB , Effect of herbicide resistance endowing Ile‐1781‐Leu and Asp‐2078‐Gly ACCase gene mutations on ACCase kinetics and growth traits in *Lolium rigidum* . J Exp Bot 66:4711–4718 (2015). 10.1093/jxb/erv248. PMC4507778 26019257

[128] Menchari Y , Chauvel B , Darmency H and Délye C , Fitness costs associated with three mutant acetyl‐coenzyme A carboxylase alleles endowing herbicide resistance in black‐grass *Alopecurus myosuroides* . J Appl Ecol 45:939–947 (2008). 10.1111/j.1365-2664.2008.01462.x.

[129] Ghanizadeh H and Harrington KC , Ecological evidence for the fitness trade‐off in Triazine resistant *Chenopodium Album* L.: can we exploit the cost of resistance? Agronomy (Basel) 9:523 (2019). 10.3390/agronomy9090523.

[130] Frenkel E , Matzrafi M , Rubin B and Peleg Z , Effects of environmental conditions on the fitness penalty in herbicide resistant *Brachypodium hybridum* . Front Plant Sci 8:94 (2017). 10.3389/fpls.2017.00094. PMC5289963 28217132

[131] Sundby C , Chow WS and Anderson JM , Effects on photosystem II function, Photoinhibition, and plant performance of the spontaneous mutation of Serine‐264 in the photosystem II reaction center D1 protein in Triazine‐resistant *Brassica napus* L. Plant Physiol 103:105–113 (1993). 10.1104/pp.103.1.105. PMC158952 12231917

[132] Jansen MAK and Pfister K , Conserved kinetics at the reducing side of reaction‐center II in photosynthetic organisms; changed kinetics in Triazine‐resistant weeds. Z Naturforsch C J Biosci 45C:441–445 (1990). 10.1515/znc-1990-0522.

[133] Malone JM , Dang HT , de Figueiredo MR , Gill G , Gaines TA and Preston C , 2,4‐D resistance due to a deletion in *IAA2* in *Sisymbrium orientale* L. carries no apparent fitness penalty. Pest Manag Sci 81:3013–3019 (2025). 10.1002/ps.8673. PMC12074621 39876824

[134] Wu C , LeClere S , Liu K , Paciorek M , Perez‐Jones A , Westra P *et al*., A dicamba resistance‐endowing *IAA16* mutation leads to significant vegetative growth defects and impaired competitiveness in kochia (*Bassia scoparia*). Pest Manag Sci 77:795–804 (2021). 10.1002/ps.6080. PMC7821297 32909332

[135] Wu C , Davis AS and Tranel PJ , Limited fitness costs of herbicide‐resistance traits in *Amaranthus tuberculatus* facilitate resistance evolution. Pest Manag Sci 74:293–301 (2018). 10.1002/ps.4706. 28834134

[136] Simmons MJ and Crow JF , Mutations affecting fitness in drosophila populations. Annu Rev Genet 11:49–78 (1977). 10.1146/annurev.ge.11.120177.000405. 413473

[137] Leon RG , Dunne JC and Gould F , The role of population and quantitative genetics and modern sequencing technologies to understand evolved herbicide resistance and weed fitness. Pest Manag Sci 77:12–21 (2021). 10.1002/ps.5988. PMC7754128 32633005

[138] Carvalho‐Moore P , Borgato EA , Cutti L , Porri A , Meiners I , Lerchl J *et al*., A rearranged *Amaranthus palmeri* extrachromosomal circular DNA confers resistance to glyphosate and glufosinate. Plant Cell 37:koaf069 (2025). 10.1093/plcell/koaf069. PMC11985328 40152451

[139] Molin WT , Yaguchi A , Blenner M and Saski CA , The EccDNA replicon: A heritable, Extranuclear vehicle that enables gene amplification and glyphosate resistance in *Amaranthus palmeri* . Plant Cell 32:2132–2140 (2020). 10.1105/tpc.20.00099. PMC7346551 32327538

[140] Patterson EL , Saski CA , Sloan DB , Tranel PJ , Westra P and Gaines TA , The draft genome of *Kochia scoparia* and the mechanism of glyphosate resistance via transposon‐mediated EPSPS tandem gene duplication. Genome Biol Evol 11:2927–2940 (2019). 10.1093/gbe/evz198. PMC6808082 31518388

[141] Hall N , Montgomery J , Chen J , Saski C , Matzrafi M , Westra P *et al*., FHY3/FAR1 transposable elements generate adaptive genetic variation in the *Bassia scoparia* genome. Pest Manag Sci 81:4393–4402 (2025). 10.1002/ps.8798. PMC12268810 40165631

[142] Salas RA , Dayan FE , Pan Z , Watson SB , Dickson JW , Scott RC *et al*., *EPSPS* gene amplification in glyphosate‐resistant Italian ryegrass (*Lolium perenne* ssp. *multiflorum*) from Arkansas. Pest Manag Sci 68:1223–1230 (2012). 10.1002/ps.3342. 22815255

[143] Lorentz L , Gaines TA , Nissen SJ , Westra P , Strek HJ , Dehne HW *et al*., Characterization of glyphosate resistance in *Amaranthus tuberculatus* populations. J Agric Food Chem 62:8134–8142 (2014). 10.1021/jf501040x. 24956036

[144] Zhang C , Johnson NA , Hall N , Tian X , Yu Q and Patterson E , Subtelomeric 5‐enolpyruvylshikimate‐3‐phosphate synthase (*EPSPS*) copy number variation confers glyphosate resistance in *Eleusine indica* . Nat Commun 14:4865 (2023). 10.1038/s41467-023-40407-6. PMC10421919 37567866

[145] Laforest M , Soufiane B , Simard MJ , Obeid K , Page E and Nurse RE , Acetyl‐CoA carboxylase overexpression in herbicide‐resistant large crabgrass (*Digitaria sanguinalis*). Pest Manag Sci 73:2227–2235 (2017). 10.1002/ps.4675. 28755464

[146] Yang J , Yu H , Cui H , Chen J and Li X , *PsbA* gene over‐expression and enhanced metabolism conferring resistance to atrazine in *Commelina communis* . Pestic Biochem Physiol 188:105260 (2022). 10.1016/j.pestbp.2022.105260. 36464365

[147] Vila‐Aiub MM , Goh SS , Gaines TA , Han H , Busi R , Yu Q *et al*., No fitness cost of glyphosate resistance endowed by massive EPSPS gene amplification in *Amaranthus palmeri* . Planta 239:793–801 (2014). 10.1007/s00425-013-2022-x. 24385093

[148] Giacomini D , Westra P and Ward SM , Impact of genetic background in fitness cost studies: an example from glyphosate‐resistant palmer Amaranth. Weed Sci 62:29–37 (2014). 10.1614/WS-D-13-00066.1.

[149] Kumar V and Jha P , Growth and reproduction of glyphosate‐resistant and susceptible populations of *Kochia scoparia* . PLoS One 10:e0142675 (2015). 10.1371/journal.pone.0142675. PMC4651312 26580558

[150] Iwakami S , Kamidate Y , Yamaguchi T , Ishizaka M , Endo M , Suda H *et al*., *CYP81A* P450s are involved in concomitant cross‐resistance to acetolactate synthase and acetyl‐CoA carboxylase herbicides in *Echinochloa phyllopogon* . New Phytol 221:2112–2122 (2019). 10.1111/nph.15552. 30347444

[151] Iwakami S , Endo M , Saika H , Okuno J , Nakamura N , Yokoyama M *et al*., Cytochrome P450 *CYP81A12* and *CYP81A21* are associated with resistance to two Acetolactate synthase inhibitors in *Echinochloa phyllopogon* . Plant Physiol 165:618–629 (2014). 10.1104/pp.113.232843. PMC4044852 24760819

[152] Figueiredo MR , Leibhart LJ , Reicher ZJ , Tranel PJ , Nissen SJ , Westra P *et al*., Metabolism of 2,4‐dichlorophenoxyacetic acid contributes to resistance in a common waterhemp (*Amaranthus tuberculatus*) population. Pest Manag Sci 74:2356–2362 (2018). 10.1002/ps.4811. 29194949

[153] Wang H , Fang J , Li X , Sun P , Gao H , Ren Y *et al*., Epigenetic regulation of CYP72A385‐mediated metabolic resistance to novel auxin herbicide Florpyrauxifen‐benzyl in *Echinochloa crus‐galli* (L.) P. Beauv. J Agric Food Chem 72:9199–9214 (2024). 10.1021/acs.jafc.4c00804. 38600742

[154] Cummins I , Wortley DJ , Sabbadin F , He Z , Coxon CR , Straker HE *et al*., Key role for a glutathione transferase in multiple‐herbicide resistance in grass weeds. Proc Natl Acad Sci USA 110:5812–5817 (2013). 10.1073/pnas.1221179110. PMC3625300 23530204

[155] Wang J , Chen J , Li X , Li D , Li Z and Cui H , Pro‐197‐Ser mutation in *ALS* and high‐Level GST activities: multiple resistance to ALS and ACCase inhibitors in *Beckmannia syzigachne* . Front Plant Sci 11:572610 (2020). 10.3389/fpls.2020.572610. PMC7556300 33101340

[156] Rangani G , Noguera M , Salas‐Perez R , Benedetti L and Roma‐Burgos N , Mechanism of resistance to *S*‐metolachlor in palmer amaranth. Front Plant Sci 12:652581 (2021). 10.3389/fpls.2021.652581. PMC7994610 33777086

[157] Pan L , Yu Q , Han H , Mao L , Nyporko A , Fan L *et al*., Aldo‐keto reductase metabolizes glyphosate and confers glyphosate resistance in *Echinochloa colona* . Plant Physiol 181:1519–1534 (2019). 10.1104/pp.19.00979. PMC6878027 31551360

[158] Zhou FY , Han H , Han YJ , Nyporko A , Yu Q , Beckie HJ *et al*., Aldo‐keto reductase may contribute to glyphosate resistance in *Lolium rigidum* . Pest Manag Sci 79:1528–1537 (2023). 10.1002/ps.7325. 36527683

[159] Cutti L , Araujo ALS , Turra GM , Rigon CAG , Angonese PS , Gallon M *et al*., High temperatures are required for expression of metabolic resistance to cyhalofop‐butyl in a biotype of *Echinochloa crus‐galli* . J Agric Food Chem 74:11461–11472 (2026). 10.1021/acs.jafc.5c16988. PMC13088228 41930952

[160] Matzrafi M , Seiwert B , Reemtsma T , Rubin B and Peleg Z , Climate change increases the risk of herbicide‐resistant weeds due to enhanced detoxification. Planta 244:1217–1227 (2016). 10.1007/s00425-016-2577-4. 27507240

[161] Matzrafi M , Shaar‐Moshe L , Rubin B and Peleg Z , Unraveling the transcriptional basis of temperature‐dependent Pinoxaden resistance in *Brachypodium hybridum* . Front Plant Sci 8:1064 (2017). 10.3389/fpls.2017.01064. PMC5478685 28680434

[162] Reade JPH , Milner LJ and Cobb AH , A role for glutathione S‐transferases in resistance to herbicides in grasses. Weed Sci 52:468–474 (2004). 10.1614/P2002-168D.

[163] Milner LJ , Reade JPH and Cobb AH , The effect of temperature on glutathione S‐transferase activity and glutathione content in *Alopecurus myosuroides* (black grass) biotypes susceptible and resistant to herbicides. Weed Res 47:106–112 (2007). 10.1111/j.1365-3180.2007.00540.x.

[164] Vila‐Aiub MM , Gundel PE , Yu Q and Powles SB , Glyphosate resistance in *Sorghum halepense* and *Lolium rigidum* is reduced at suboptimal growing temperatures. Pest Manag Sci 69:228–232 (2013). 10.1002/ps.3464. 23255521

[165] Ghanizadeh H , Harrington KC and James TK , Glyphosate‐resistant population of *Lolium perenne* loses resistance at winter temperatures. N Z J Agric Res 58:423–431 (2015). 10.1080/00288233.2015.1076490.

[166] Guo W , Zhang C , Wang S , Zhang T and Tian X , Temperature influences glyphosate efficacy on glyphosate‐resistant and ‐susceptible goosegrass (*Eleusine indica*). Front Plant Sci 14:1169726 (2023). 10.3389/fpls.2023.1169726. PMC10073670 37035058

[167] He S , Tian J , Ouyang Y , Liao Y , Yu Q , Bai L *et al*., Glyphosate resistance in *Eleusine indica*: involvement of *CYP71AK44* in addition to *EPSPS* gene overexpression. J Agric Food Chem 72:23758–23765 (2024). 10.1021/acs.jafc.4c07765. 39377301

[168] Shrestha A , Budhathoki S and Steinhauer K , Temperature effects on glyphosate resistance in California populations of Junglerice. Agron J 110:1624–1626 (2018). 10.2134/agronj2018.02.0104.

[169] Pan L , Yu Q , Wang J , Han H , Mao L , Nyporko A *et al*., An ABCC‐type transporter endowing glyphosate resistance in plants. Proc Natl Acad Sci USA 118:e2100136118 (2021). 10.1073/pnas.2100136118. PMC8072331 33846264

[170] Rudell EC , Aarthy T , Shyam C , Borgato EA , Kaur S and Jugulam M , High temperature increases 2,4‐D metabolism in resistant *Amaranthus palmeri* . Weed Sci 71:217–223 (2023). 10.1017/wsc.2023.26.

[171] Shyam C , Jhala AJ , Kruger G and Jugulam M , Rapid metabolism increases the level of 2,4‐D resistance at high temperature in common waterhemp (*Amaranthus tuberculatus*). Sci Rep 9:16695 (2019). 10.1038/s41598-019-53164-8. PMC6853974 31723191

[172] Küpper A , Peter F , Zöllner P , Lorentz L , Tranel PJ , Beffa R *et al*., Tembotrione detoxification in 4‐hydroxyphenylpyruvate dioxygenase (HPPD) inhibitor‐resistant palmer amaranth (*Amaranthus palmeri* S. Wats.). Pest Manag Sci 74:2325–2334 (2018). 10.1002/ps.4786. 29105299

[173] Rigon CAG , Küpper A , Sparks C , Montgomery J , Peter F , Schepp S *et al*., Function of cytochrome P450 *CYP72A1182* in metabolic herbicide resistance evolution in *Amaranthus palmeri* populations. J Exp Bot 76:2891–2907 (2025). 10.1093/jxb/eraf114. PMC12223498 40067326

[174] Ge X , D'Avignon DA , Ackerman JJ and Sammons RD , Rapid vacuolar sequestration: the horseweed glyphosate resistance mechanism. Pest Manag Sci 66:345–348 (2010). 10.1002/ps.1911. PMC3080097 20063320

[175] Brunharo CACG and Hanson BD , Vacuolar sequestration of Paraquat is involved in the resistance mechanism in *Lolium perenne* L. spp. *multiflorum* . Front Plant Sci 8:1485 (2017). 10.3389/fpls.2017.01485. PMC5575147 28890724

[176] Goldberg‐Cavalleri A , Onkokesung N , Franco‐Ortega S and Edwards R , ABC transporters linked to multiple herbicide resistance in blackgrass (*Alopecurus myosuroides*). Front Plant Sci 14:1082761 (2023). 10.3389/fpls.2023.1082761. PMC10063862 37008473

[177] Dalazen G , Pisoni A , Menegaz C and Merotto Jr A , Low temperature reverses the resistance to glyphosate in hairy fleabane (*Conyza bonariensis*). J Plant Prot Res 59:433–440 (2019). 10.24425/jppr.2019.131267.

[178] Ge X , D'Avignon DA , Ackerman JJ , Duncan B , Spaur MB and Sammons RD , Glyphosate‐resistant horseweed made sensitive to glyphosate: low‐temperature suppression of glyphosate vacuolar sequestration revealed by 31P NMR. Pest Manag Sci 67:1215–1221 (2011). 10.1002/ps.2169. 21495156

[179] Tani E , Chachalis D , Travlos IS and Bilalis D , Environmental conditions influence induction of key ABC‐transporter genes affecting glyphosate resistance mechanism in *Conyza canadensis* . Int J Mol Sci 17:342 (2016). 10.3390/ijms17040342. PMC4848879 27104532

[180] Yu Q , Cairns A and Powles SB , Paraquat resistance in a population of *Lolium rigidum* . Funct Plant Biol 31:247–254 (2004). 10.1071/FP03234. 32688896

[181] Purba E , Preston C and Powles SB , The mechanism of resistance to paraquat is strongly temperature dependent in resistant *Hordeum leporinum* link and *H. Glaucum* Steud. Planta 196:464–468 (1995). 10.1007/BF00203645.

[182] Robertson BK and Alexander M , Growth‐linked and cometabolic biodegradation: possible reason for occurrence or absence of accelerated pesticide biodegradation. Pestic Sci 41:311–318 (1994). 10.1002/ps.2780410405.

[183] Hu T , Fang H , Pan Q , Xu H , Lv T , Fan X *et al*., Seed microbiome‐mediated herbicide resistance evolution in weeds. New Phytol 242:333–343 (2024). 10.1111/nph.19459. 38062878

[184] Liu K , Luo K , Mao A , Pan L , Yan B , Wu J *et al*., Endophytes enhance Asia minor bluegrass (*Polypogon fugax*) resistance to quizalofop‐p‐ethyl. Plant Soil 450:373–384 (2020). 10.1007/s11104-020-04509-0.

[185] Malook SU , Arora AK and Wong ACN , The role of microbiomes in shaping insecticide resistance: current insights and emerging paradigms. Curr Opin Insect Sci 69:101346 (2025). 10.1016/j.cois.2025.101346. 39999947

[186] Gressel J , Microbiome facilitated pest resistance: potential problems and uses. Pest Manag Sci 74:511–515 (2018). 10.1002/ps.4777. 29072801

[187] Cusaro CM , Capelli E , Picco AM and Brusoni M , Incidence of resistance to ALS and ACCase inhibitors in *Echinochloa* species and soil microbial composition in northern Italy. Sci Rep 14:10544 (2024). 10.1038/s41598-024-59856-0. PMC11078947 38719860

[188] Zhao HH , Li HY and Kong CH , Penoxsulam‐resistant barnyardgrass‐mediated rhizosphere microbial communities affect the growth of rice. Pest Manag Sci 79:2664–2674 (2023). 10.1002/ps.7445. 36883589

[189] Van Horn CR , Moretti ML , Robertson RR , Segobye K , Weller SC , Young BG *et al*., Glyphosate resistance in *Ambrosia trifida*: part 1. Novel rapid cell death response to glyphosate. Pest Manag Sci 74:1071–1078 (2018). 10.1002/ps.4567. 28266132

[190] de Queiroz ARS , Delatorre CA , Lucio FR , Rossi CVS , Zobiole LHS and Merotto A , Rapid necrosis: a novel plant resistance mechanism to 2,4‐D. Weed Sci 68:6–18 (2020). 10.1017/wsc.2019.65.

[191] Leal JFL , Souza A d S , Borella J , Araujo ALS , Langaro AC , Alves MM *et al*., Rapid photosynthetic and physiological response of 2,4‐D–resistant Sumatran fleabane (*Conyza sumatrensis*) to 2,4‐D as a survival strategy. Weed Sci 70:298–308 (2022). 10.1017/wsc.2022.10.

[192] Moretti ML , Van Horn CR , Robertson R , Segobye K , Weller SC , Young BG *et al*., Glyphosate resistance in *Ambrosia trifida*: part 2. Rapid response physiology and non‐target‐site resistance. Pest Manag Sci 74:1079–1088 (2018). 10.1002/ps.4569. 28276187

[193] Angonese PS , de Queiroz ARS , Angonese LS , Machado FM , Napier R , Markus C *et al*., Rapid necrosis: implications of environmental conditions and plant growth stage on 2,4‐D resistance and effect of other auxinic herbicides in Sumatran fleabane (*Conyza sumatrensis*). Weed Technol 37:174–184 (2023). 10.1017/wet.2023.21.

[194] Griffith SM , Brewer TG and Steiner JJ , Thermal dependence of the apparent km of glutathione reductase from three wetland grasses and maize. Ann Bot 87:599–603 (2001). 10.1006/anbo.2001.1380.

[195] Seder S , Temperature Dependence on Enzymatic Activity of A82F F87A Mutant and Wild‐Type Cytochrome P450 BM‐3. Montclair State University: Undergraduate Theses (2020). Available: https://digitalcommons.montclair.edu/undergraduate_theses/1.

[196] Diffenbaugh NS and Barnes EA , Data‐driven predictions of the time remaining until critical global warming thresholds are reached. Proc Natl Acad Sci USA 120:e2207183120 (2023). 10.1073/pnas.2207183120. PMC9963891 36716375

[197] Délye C , Jasieniuk M and Le Corre V , Deciphering the evolution of herbicide resistance in weeds. Trends Genet 29:649–658 (2013). 10.1016/j.tig.2013.06.001. 23830583

[198] Pittendrigh BR , Huesing J , Walters KR , Olds BP , Steele LD , Sun L *et al*., Chapter 11 – negative cross‐resistance: history, present status, and emerging opportunities, in Insect Resistance Management (Second Edition), ed. by Onstad DW . Academic Press, San Diego, pp. 373–401 (2014). 10.1016/C2011-0-07259-9.

[199] Cutti L , Rigon CAG , Kaspary TE , Turra GM , Markus C and Merotto A , Negative cross‐resistance to clomazone in imazethapyr‐resistant *Echinochloa crus‐galli* caused by increased metabolization. Pestic Biochem Physiol 178:104918 (2021). 10.1016/j.pestbp.2021.104918. 34446194

[200] Xu Y , Li S , Hao L , Li X and Zheng M , Tribenuron‐methyl‐resistant *Descurainia sophia* L. exhibits negative cross‐resistance to imazethapyr conferred by a Pro197Ser mutation in acetolactate synthase and reduced metabolism. Pest Manag Sci 78:1467–1473 (2022). 10.1002/ps.6764. 34951107

[201] HRAC – Herbicide Resistance Action Committee , HRAC Global Herbicide MoA Classification 2024 (2024). Available: https://hracglobal.com/tools/2024-hrac-global-herbicide-moa-classification.

[202] Kless H , Oren‐Shamir M , Malkin S , McIntosh L and Edelman M , The D‐E region of the D1 protein is involved in multiple quinone and herbicide interactions in photosystem II. Biochemistry 33:10501–10507 (1994). 10.1021/bi00200a035. 8068689

[203] Poston DH , Hirata CM and Wilson HP , Response of acetolactate synthase from imidazolinone‐susceptible and ‐resistant smooth pigweed to ALS inhibitors. Weed Sci 50:306–311 (2002). 10.1614/0043-1745(2002)050[0306:ROASFI]2.0.CO;2.

[204] Dimaano NG , Yamaguchi T , Fukunishi K , Tominaga T and Iwakami S , Functional characterization of cytochrome P450 *CYP81A* subfamily to disclose the pattern of cross‐resistance in *Echinochloa phyllopogon* . Plant Mol Biol 102:403–416 (2020). 10.1007/s11103-019-00954-3. 31898147

[205] Gershater MC , Cummins I and Edwards R , Role of a carboxylesterase in herbicide bioactivation in *Arabidopsis thaliana* . J Biol Chem 282:21460–21466 (2007). 10.1074/jbc.M701985200. 17519238

[206] Ruiz‐Santaella JP , Heredia A and Prado RD , Basis of selectivity of cyhalofop‐butyl in *Oryza sativa* L. Planta 223:191–199 (2006). 10.1007/s00425-005-0075-1. 16160841

[207] Ferhatoglu Y and Barrett M , Studies of clomazone mode of action. Pestic Biochem Physiol 85:7–14 (2006). 10.1016/j.pestbp.2005.10.002.

[208] Ferhatoglu Y , Avdiushko S and Barrett M , The basis for the safening of clomazone by phorate insecticide in cotton and inhibitors of cytochrome P450s. Pestic Biochem Physiol 81:59–70 (2005).

[209] Suda H , Kubo T , Yoshimoto Y , Tanaka K , Tanaka S , Uchino A *et al*., Transcriptionally linked simultaneous overexpression of P450 genes for broad‐spectrum herbicide resistance. Plant Physiol 192:3017–3029 (2023). 10.1093/plphys/kiad286. PMC10400030 37195199

[210] Yasuor H , Zou W , Tolstikov VV , Tjeerdema RS and Fischer AJ , Differential oxidative metabolism and 5‐ketoclomazone accumulation are involved in *Echinochloa phyllopogon* resistance to clomazone. Plant Physiol 153:319–326 (2010). 10.1104/pp.110.153296. PMC2862431 20207709

[211] Guo F , Iwakami S , Yamaguchi T , Uchino A , Sunohara Y and Matsumoto H , Role of *CYP81A* cytochrome P450s in clomazone metabolism in *Echinochloa phyllopogon* . Plant Sci 283:321–328 (2019). 10.1016/j.plantsci.2019.02.010. 31128703

[212] Pandian BA , Sathishraj R , Prasad PVV and Jugulam M , A single gene inherited trait confers metabolic resistance to chlorsulfuron in grain sorghum (*Sorghum bicolor*). Planta 253:48 (2021). 10.1007/s00425-020-03563-3. 33484360

[213] Norsworthy JK , Ward SM , Shaw DR , Llewellyn RS , Nichols RL , Webster TM *et al*., Reducing the risks of herbicide resistance: best management practices and recommendations. Weed Sci 60:31–62 (2012). 10.1614/WS-D-11-00155.1.

[214] Stadler LJ , Mutations in barley induced by x‐rays and radium. Science 68:186–187 (1928). 10.1126/science.68.1756.186. 17774921

[215] Jander G , Baerson SR , Hudak JA , Gonzalez KA , Gruys KJ and Last RL , Ethylmethanesulfonate saturation mutagenesis in Arabidopsis to determine frequency of herbicide resistance. Plant Physiol 131:139–146 (2003). 10.1104/pp.102.010397. PMC166794 12529522

[216] Preston C and Powles SB , Evolution of herbicide resistance in weeds: initial frequency of target site‐based resistance to acetolactate synthase‐inhibiting herbicides in *Lolium rigidum* . Heredity (Edinb) 88:8–13 (2002). 10.1038/sj.hdy.6800004. 11813100

[217] Wrubel RP and Gressel J , Are herbicide mixtures useful for delaying the rapid evolution of resistance? A case study. Weed Technol 8:635–648 (1994). 10.1017/S0890037X00039828.

[218] Busi R , Powles SB , Beckie HJ and Renton M , Rotations and mixtures of soil‐applied herbicides delay resistance. Pest Manag Sci 76:487–496 (2020). 10.1002/ps.5534. 31251459

[219] Comont D , Lowe C , Hull R , Crook L , Hicks HL , Onkokesung N *et al*., Evolution of generalist resistance to herbicide mixtures reveals a trade‐off in resistance management. Nat Commun 11:3086 (2020). 10.1038/s41467-020-16896-0. PMC7303185 32555156

[220] Suda H , Yoshimoto Y , Kubo T , Tanaka K , Tanaka S , Yamaguchi Y *et al*., Simultaneous overexpression of multiple herbicide‐metabolizing genes for broad‐spectrum resistance in an agricultural weed *Echinochloa phyllopogon* . bioRxiv preprint:2025.2002.2020.639371 (2022). 10.1101/2022.01.02.474499.

[221] Cutti L , Rigon CAG , Girelli N , Angonese PS , Ulguim ADR and Merotto A , The safener isoxadifen‐ethyl confers fenoxaprop‐p‐ethyl resistance on a biotype of *Echinochloa crus‐galli* . Pest Manag Sci 78:2287–2298 (2022). 10.1002/ps.6851. 35220677

[222] Brunharo CACG , Takano HK , Mallory‐Smith CA , Dayan FE and Hanson BD , Role of glutamine Synthetase Isogenes and herbicide metabolism in the mechanism of resistance to Glufosinate in *Lolium perenne* L. spp. *multiflorum* biotypes from Oregon. J Agric Food Chem 67:8431–8440 (2019). 10.1021/acs.jafc.9b01392. 31067047

[223] Oliveira MC , Gaines TA , Dayan FE , Patterson EL , Jhala AJ and Knezevic SZ , Reversing resistance to tembotrione in an *Amaranthus tuberculatus* (var. *rudis*) population from Nebraska, USA with cytochrome P450 inhibitors. Pest Manag Sci 74:2296–2305 (2018). 10.1002/ps.4697. 28799707

[224] Schwarz M , Eno RFM , Freitag‐Pohl S , Coxon CR , Straker HE , Wortley DJ *et al*., Flavonoid‐based inhibitors of the phi‐class glutathione transferase from black‐grass to combat multiple herbicide resistance. Org Biomol Chem 19:9211–9222 (2021). 10.1039/d1ob01802g. PMC8564858 34643629

[225] Westerveld DB , Soltani N , Hooker DC , Robinson DE , Tranel PJ , Laforest M *et al*., Biologically effective dose of metribuzin applied preemergence and postemergence for the control of waterhemp (*Amaranthus tuberculatus*) with different mechanisms of resistance to photosystem II–inhibiting herbicides. Weed Sci 69:631–641 (2021). 10.1017/wsc.2021.50.

[226] Gafni R , Nassar JA , Matzrafi M , Blank L and Eizenberg H , Unraveling the reasons for failure to control *Amaranthus albus*: insights into herbicide application at different growth stages, temperature effect, and herbicide resistance on a regional scale. Pest Manag Sci 80:4757–4769 (2024). 10.1002/ps.8192. 38809094

[227] Wu C , Varanasi V and Perez‐Jones A , A nondestructive leaf‐disk assay for rapid diagnosis of weed resistance to multiple herbicides. Weed Sci 69:274–283 (2021). 10.1017/wsc.2021.15.

[228] Shaner DL , Nadler‐Hassar T , Henry WB and Koger CH , A rapid in vivo shikimate accumulation assay with excised leaf discs. Weed Sci 53:769–774 (2005). 10.1614/WS-05-009R.1.

[229] Nol N , Tsikou D , Eid M , Livieratos IC and Giannopolitis CN , Shikimate leaf disc assay for early detection of glyphosate resistance in *Conyza canadensis* and relative transcript levels of *EPSPS* and *ABC* transporter genes. Weed Res 52:233–241 (2012). 10.1111/j.1365-3180.2012.00911.x.

[230] Brosnan JT , Vargas JJ , Breeden GK , Grier L , Aponte RA , Tresch S *et al*., A new amino acid substitution (ala‐205‐Phe) in acetolactate synthase (ALS) confers broad spectrum resistance to ALS‐inhibiting herbicides. Planta 243:149–159 (2016). 10.1007/s00425-015-2399-9. PMC4698308 26353912

[231] Zhang Z , Ban Y , Wei J , Wu Q , Dong L and Feng Z , Rapid resistance detection of *Amaranthus retroflexus* to Fomesafen via Kompetitive allele‐specific PCR (KASP). Plants (Basel) 14:515 (2025). 10.3390/plants14040515. PMC11858902 40006774

[232] Ghanizadeh H , Griffiths AG , Buddenhagen CE , Anderson CB and Harrington KC , A PCR plus restriction enzyme‐based technique for detecting target‐enzyme mutations at position Pro‐106 in glyphosate‐resistant *Lolium perenne* . PLoS One 16:e0246028 (2021). 10.1371/journal.pone.0246028. PMC7853469 33529261

[233] Ferreira LAI , Oliveira JRS , Constantin J and Brunharo C , Evolution of ACCase‐inhibitor resistance in *Chloris virgata* is conferred by a Trp2027Cys mutation in the herbicide target site. Pest Manag Sci 79:5220–5229 (2023). 10.1002/ps.7723. 37592752

[234] Yu J , Gao H , Pan L , Yao Z and Dong L , Mechanism of resistance to cyhalofop‐butyl in Chinese sprangletop (*Leptochloa chinensis* (L.) Nees). Pestic Biochem Physiol 143:306–311 (2017). 10.1016/j.pestbp.2016.11.001. 29183606

[235] Notomi T , Mori Y , Tomita N and Kanda H , Loop‐mediated isothermal amplification (LAMP): principle, features, and future prospects. J Microbiol 53:1–5 (2015). 10.1007/s12275-015-4656-9. 25557475

[236] Yin F , Wang M , Liao M , Cao H and Zhao N , Detection of two common ACCase mutations associated with high levels of fenoxaprop‐P‐ethyl resistance in shortawn foxtail (*Alopecurus aequalis*) using loop‐mediated isothermal amplification. Weed Sci 72:693–702 (2024). 10.1017/wsc.2024.44.

[237] Milani A , Panozzo S , Grazia TM and Scarabel L , Development of a rapid detection assay for acetolactate synthase inhibitors resistance in three Amaranthus weed species through loop‐mediated isothermal amplification. J Sci Food Agric 104:5522–5532 (2024). 10.1002/jsfa.13385. 38358049

[238] Zhang H , Xu Y , Fohlerova Z , Chang H , Iliescu C and Neuzil P , LAMP‐on‐a‐chip: revising microfluidic platforms for loop‐mediated DNA amplification. Trends Anal Chem 113:44–53 (2019). 10.1016/j.trac.2019.01.015. PMC7112807 32287531

[239] Xiao X , Lin Z , Huang X , Lu J , Zhou Y , Zheng L *et al*., Rapid and sensitive detection of *Vibrio vulnificus* using CRISPR/Cas12a combined with a recombinase‐aided amplification assay. Front Microbiol 12:767315 (2021). 10.3389/fmicb.2021.767315. PMC8566878 34745075

[240] Liu S , Lun J , Hu L and Pan L , Rapid and field‐deployable detection of the S59G mutation in glutamine Synthetase endowing Glufosinate resistance in *Eleusine indica* . J Agric Food Chem 73:23292–23299 (2025). 10.1021/acs.jafc.5c08583. 40897678

[241] Liu S , Lun J , Zhan Y , Li Z , Tian J , Zhang C *et al*., CRISPR/Cas12a combined with RAA for on‐site detection of ALS W574L mutation in three *Alopecurus* species: A visual approach for herbicide resistance monitoring. J Agric Food Chem 73:8907–8914 (2025). 10.1021/acs.jafc.5c02188. 40177728

[242] Liu S , Cao Z , Lun J , Zhang C and Pan L , Rapid detection of ACCase W2027C mutation in *Leptochloa chinensis* (L.) Nees using RAA‐CRISPR/Cas12a: a sensitive and visual tool for herbicide resistance diagnosis. Pest Manag Sci 81:4769–4776 (2025). 10.1002/ps.8840. 40265707

[243] Yin H , Ge Z , Yao S , Chen G , Yuan S , Yang Q *et al*., Resistance to ACCase‐inhibiting herbicides conferred by the Ile‐1781‐Val mutation in *Leptochloa fusca* and visual detection of target mutations by RAA‐CRISPR/Cas12a. Pest Manag Sci 81:5581–5588 (2025). 10.1002/ps.8911. 40396330

[244] Ban Y , Zhang Z , Wei J , Xu G , Ma Y , Yin S *et al*., Establishment of a CRISPR‐based system for rapidly detecting the target‐site resistance of American sloughgrass (*Beckmannia syzigachne*) to Pinoxaden. Pest Manag Sci 81:5649–5658 (2025). 10.1002/ps.8919. 40415729

[245] Wu D , Shen E , Jiang B , Feng Y , Tang W , Lao S *et al*., Genomic insights into the evolution of *Echinochloa* species as weed and orphan crop. Nat Commun 13:689 (2022). 10.1038/s41467-022-28359-9. PMC8814039 35115514

[246] Onkokesung N , Brazier‐Hicks M , Tetard‐Jones C , Bentham A and Edwards R , Molecular diagnostics for real‐time determination of herbicide resistance in wild grasses. J Biotechnol 358:64–66 (2022). 10.1016/j.jbiotec.2022.09.004. 36100138

[247] Allison SJ and Milner J , RNA interference by single‐ and double‐stranded siRNA with a DNA extension containing a 3' nuclease‐resistant mini‐hairpin structure. Mol Ther Nucleic Acids 2:e141 (2014). 10.1038/mtna.2013.68. PMC3894584 24399205

[248] Zabala‐Pardo D , Gaines TA , Lamego FP and Avila LA , RNAi as a tool for weed management: challenges and opportunities. Adv Weed Sci 40:e020220096 (2022). 10.51694/AdvWeedSci/2022;40:seventy-five006.

[249] Li J , Zhang Y , Li L , Wei S , Huang Z , Chen L *et al*., Expression of *Echinochloa oryzoides CYP71A1* and *GSTU23* catalyzes the metabolism of thiobencarb, thereby conferring resistance. Pestic Biochem Physiol 213:106555 (2025). 10.1016/j.pestbp.2025.106555. 40744595

[250] Callaway E , Gene drives thwarted by emergence of resistant organisms. Nature 542:15 (2017). 10.1038/542015. 28150791

[251] Raiyemo DA , Montgomery JS , Cutti L , Abdollahi F , Llaca V , Fengler K *et al*., Chromosome‐level assemblies of *Amaranthus palmeri*, *Amaranthus retroflexus*, and *Amaranthus hybridus* allow for genomic comparisons and identification of a sex‐determining region. Plant J 121:e70027 (2025). 10.1111/tpj.70027. PMC11850965 39994881

[252] Gomes AC , Montgomery J , Araujo ALS , Morran S , Cutti L , Patterson E *et al*., Chromosome‐Level assemblies of the allohexaploid genomes of *Conyza sumatrensis* and *Conyza bonariensis* . Genome Biol Evol 17:evaf065 (2025). 10.1093/gbe/evaf065. PMC12008746 40181513

[253] Laforest M , Martin SL , Bisaillon K , Soufiane B , Meloche S and Page E , A chromosome‐scale draft sequence of the Canada fleabane genome. Pest Manag Sci 76:2158–2169 (2020). 10.1002/ps.5753. 31951071

[254] Yang W , Wang B , Lei G , Chen G and Liu D , Advances in nanocarriers to improve the stability of dsRNA in the environment. Front Bioeng Biotechnol 10:974646 (2022). 10.3389/fbioe.2022.974646. PMC9424858 36051593

[255] Mujtaba M , Wang D , Carvalho LB , Oliveira JL , Pereira AES , Sharif R *et al*., Nanocarrier‐mediated delivery of miRNA, RNAi, and CRISPR‐Cas for plant protection: current trends and future directions. ACS Agric Sci Technol 1:417–435 (2021). 10.1021/acsagscitech.1c00146.

[256] Polito RA , Viana VE , Camargo ER and Avila LA , Precise transcript targeting using RNAi for weed control. Adv Weed Sci 42:e020240014 (2024). 10.51694/AdvWeedSci/2024;42:00016.

[257] Mai J , Liao L , Ling R , Guo X , Lin J , Mo B *et al*., Study on RNAi‐based herbicide for *Mikania micrantha* . Synth Syst Biotechnol 6:437–445 (2021). 10.1016/j.synbio.2021.11.005. PMC8637008 34901482

[258] Narva K , Otto E , Sridharan K , Flannagan R , Barnes E , Mézin L *et al*., Calantha™: the first commercialized Sprayable DsRNA product for insect control, in RNA Interference in Agriculture: Basic Science to Applications, ed. by Smagghe G , Palli SR and Swevers L . Springer Nature, Cham, pp. 679–715 (2025). 10.1007/978-3-031-81549-2_27.

[259] Rodrigues TB , Mishra SK , Sridharan K , Barnes ER , Alyokhin A , Tuttle R *et al*., First Sprayable double‐stranded RNA‐based biopesticide product targets *proteasome* subunit Beta Type‐5 in Colorado potato beetle (*Leptinotarsa decemlineata*). Front Plant Sci 12:728652 (2021). 10.3389/fpls.2021.728652. PMC8650841 34887882

[260] Linyu W , Lianjun Z , Ning L , Xiwu G and Xiaoning L , Effect of RNAi targeting *CYP6CY3* on the growth, development and insecticide susceptibility of *Aphis gossypii* by using nanocarrier‐based transdermal dsRNA delivery system. Pestic Biochem Physiol 177:104878 (2021). 10.1016/j.pestbp.2021.104878. 34301368

[261] Aderolu IA , Oyerinde AA and Agunloye OOM , From resistance breakers to RNAi: targeting *Spodoptera frugiperda* detoxification genes for sustainable management. Research Square (2025). 10.21203/rs.3.rs-6687516/v1.

[262] Lv H , Li X , Li J , Yu C , Zeng Q , Ning G *et al*., Overcoming resistance in insect pest with a nanoparticle‐mediated dsRNA and insecticide co‐delivery system. Chem Eng J 475:146239 (2023). 10.1016/j.cej.2023.146239.

[263] Luo Z , Zeng Y , Qian H , Zhao H , Yu Q , Bai L *et al*., Conjoint genome‐transcriptome analysis to identify the conserved *ABCC3* gene involved in glyphosate resistance in *Lolium* spp. J Agric Food Chem 73:27115–27125 (2025). 10.1021/acs.jafc.5c10416. 41082625

[264] Wu L , Luo Z , Lu Y , Li J , Liu M , Cai H *et al*., Chromosome‐level genome assembly of triploid *Cyperus rotundus* uncovers allelic expression patterns and herbicide resistance mechanisms. Plant Commun 7:101624 (2026). 10.1016/j.xplc.2025.101624. PMC12903389 41305888

[265] Chen W , Liao Y , Bai D , Yu Q , Bai L and Pan L , First case of glyphosate resistance in *Polypogon fugax*: possible involvement of P450‐based mechanisms. Pest Manag Sci 81:3349–3357 (2025). 10.1002/ps.8704. 39912321

[266] Tian J , Bai D , He S , Li Z , Bai L and Pan L , Overexpression of cytochrome P450 *CYP71AF43* contributing resistance to fenoxaprop‐P‐ethyl in *Alopecurus myosuroides* from China. Pestic Biochem Physiol 209:106355 (2025). 10.1016/j.pestbp.2025.106355. 40082045

[267] Zhou FY , Lu H , Huang X , Han Y , Zhang Y , Han H *et al*., Cytochrome *CYP72A15* may play a role in metabolic resistance to mesotrione in wild radish. Pestic Biochem Physiol 210:106380 (2025). 10.1016/j.pestbp.2025.106380. 40262865

[268] Zhang L , Du Y , Wang J , Deng Y , Wang T , Bai T *et al*., *CYP72A15* confers resistance against penoxsulam to *Echinochloa phyllopogon* . Pestic Biochem Physiol 214:106427 (2025). 10.1016/j.pestbp.2025.106427. 40915780

[269] Saika H , Horita J , Taguchi‐Shiobara F , Nonaka S , Nishizawa‐Yokoi A , Iwakami S *et al*., A novel rice cytochrome P450 gene, *CYP72A31*, confers tolerance to acetolactate synthase‐inhibiting herbicides in rice and Arabidopsis. Plant Physiol 166:1232–1240 (2014). 10.1104/pp.113.231266. PMC4226355 24406793

[270] Shen J , Yang Q , Hao L , Zhang L , Li X and Zheng M , The metabolism of a novel cytochrome P450 (*CYP77B34*) in Tribenuron‐methyl‐resistant *Descurainia sophia* L. to herbicides with different mode of actions. Int J Mol Sci 23:5812 (2022). 10.3390/ijms23105812. PMC9147942 35628621

[271] Zheng T , Yu X , Sun Y , Zhang Q , Zhang X , Tang M *et al*., Expression of a cytochrome P450 gene from Bermuda grass *Cynodon dactylon* in soybean confers tolerance to multiple herbicides. Plants (Basel) 11:949 (2022). 10.3390/plants11070949. PMC9002376 35406929

[272] Yamaguchi T , Matsumura K , Iwakami S , Sunohara Y and Matsumoto H , Heterologous expression of *CYP81A6* from rice (*Oryza sativa*) in *Escherichia coli* and structural analyses of bensulfuron‐methyl metabolites. Weed Biol Manag 21:164–171 (2021). 10.1111/wbm.12235.

[273] Pan G , Zhang X , Liu K , Zhang J , Wu X , Zhu J *et al*., Map‐based cloning of a novel rice cytochrome P450 gene *CYP81A6* that confers resistance to two different classes of herbicides. Plant Mol Biol 61:933–943 (2006). 10.1007/s11103-006-0058-z. 16927205

[274] Guo F , Endo M , Yamaguchi T , Uchino A , Sunohara Y , Matsumoto H *et al*., Investigation of clomazone‐tolerance mechanism in a long‐grain cultivar of rice. Pest Manag Sci 77:2454–2461 (2021). 10.1002/ps.6274. 33432689

[275] Liu C , Liu S , Wang F , Wang Y and Liu K , Expression of a rice *CYP81A6* gene confers tolerance to bentazon and sulfonylurea herbicides in both Arabidopsis and tobacco. Plant Cell Tissue Organ Cult 109:419–428 (2012). 10.1007/s11240-011-0106-5.

[276] Zhang Y , Zhang Q , Liu Q , Zhao Y , Xu W , Hong C *et al*., Fine mapping and functional validation of the maize nicosulfuron‐resistance gene *CYP81A9* . Front Plant Sci 15:1443413 (2024). 10.3389/fpls.2024.1443413. PMC11328016 39157517

[277] Nordby JN , Williams MM , Pataky JK , Riechers DE and Lutz JD , A common genetic basis in sweet corn inbred Cr1 for cross sensitivity to multiple cytochrome P450‐metabolized herbicides. Weed Sci 56:376–382 (2008). 10.1614/WS-07-145.1.

[278] Choe E and Williams MM 2nd , Expression and comparison of sweet corn *CYP81A9*s in relation to nicosulfuron sensitivity. Pest Manag Sci 76:3012–3019 (2020). 10.1002/ps.5848. 32248609

[279] Chayapakdee P , Hengphasatporn K , Yamaguchi T , Dimaano NG , Shigeta Y , Sunohara Y *et al*., Promiscuous cytochrome P450s confer metabolic resistance to synthetic auxin herbicides in the weedy grass *Echinochloa phyllopogon*. bioRxiv preprint (2025). 10.1101/2025.07.29.667533.

[280] Pan L , Guo Q , Wang J , Shi L , Yang X , Zhou Y *et al*., *CYP81A68* confers metabolic resistance to ALS and ACCase‐inhibiting herbicides and its epigenetic regulation in *Echinochloa crus‐galli* . J Hazard Mater 428:128225 (2022). 10.1016/j.jhazmat.2022.128225. 35032953

[281] Yao S , Yin H , Li Y , Yang Q , Yuan S and Deng W , Cytochrome P450 *CYP81A104* in *Eleusine indica* confers resistance to multiherbicide with different modes of action. Pest Manag Sci 80:5791–5798 (2024). 10.1002/ps.8310. 39003629

[282] Ge Z , Lu D , Yin H , Yuan S , Yang X , Yang Q *et al*., Overexpression of a cytochrome P450 gene, *CYP81Q32*, endows resistance to HPPD‐inhibiting herbicides in *Leptochloa chinensis* . Pestic Biochem Physiol 221:107141 (2026). 10.1016/j.pestbp.2026.107141. 42264742

[283] Qi J , Liu Z , Ouyang Y , Wei L , Lu Y , Zhao S *et al*., *EcCYP89B16* confers metabolic herbicide resistance and is associated with Calvin cycle adjustments in *Echinochloa crus‐galli* . Plant Physiol 201:kiag161 (2026). 10.1093/plphys/kiag161. 41877616

[284] Shen J , Yang Q , Xu F , Han Y , Li Y and Zheng M , Effects of amino acid mutation in cytochrome P450 (*CYP96A146*) of *Descurainia sophia* on the metabolism and resistance to Tribenuron‐methyl. J Agric Food Chem 73:370–379 (2025). 10.1021/acs.jafc.4c10217. 39698796

[285] Bao J , Zhou J , Xie Z , Zou M , Napier R and Li J , *CYP99A2* from *Aegilops tauschii* metabolizes pyroxsulam but not mesosulfuron‐methyl, causing different natural sensitivity to two herbicides. Pest Manag Sci 81:6210–6219 (2025). 10.1002/ps.8967. 40497426

[286] Bai S , Yin M , Lyu Q , Jiang B and Li L , Cytochrome P450 *BsCYP99A44* and *BsCYP704A177* confer metabolic resistance to ALS herbicides in *Beckmannia syzigachne* . Int J Mol Sci 23:12175 (2022). 10.3390/ijms232012175. PMC9602494 36293032

[287] Yin F , Wang Z , Jiang M , Liao M , Cao H and Zhao N , Two cytochrome P450s, *CYP709B1* and *CYP704C1*, play essential roles in metabolism‐based multiple herbicide resistance in American Sloughgrass (*Beckmannia syzigachne* (Steud.) Fernald). J Agric Food Chem 72:14126–14140 (2024). 10.1021/acs.jafc.4c02674. 38861684

[288] Zhao N , Yan Y , Liu W and Wang J , Cytochrome P450 *CYP709C56* metabolizing mesosulfuron‐methyl confers herbicide resistance in *Alopecurus aequalis* . Cell Mol Life Sci 79:205 (2022). 10.1007/s00018-022-04171-y. PMC11072224 35334005

[289] Thyssen GN , Naoumkina M , McCarty JC , Jenkins JN , Florane C , Li P *et al*., The P450 gene *CYP749A16* is required for tolerance to the sulfonylurea herbicide trifloxysulfuron sodium in cotton (*Gossypium hirsutum* L.). BMC Plant Biol 18:186 (2018). 10.1186/s12870-018-1414-2. PMC6131939 30200872

[290] Gao H , Wei J , Zhang X , Yu J , Li J , Feng Z *et al*., Glutathione *S*‐transferase gene *GST1* metabolizing Penoxsulam and conferring Penoxsulam resistance in *Echinochloa phyllopogon* . J Agric Food Chem 73:10870–10880 (2025). 10.1021/acs.jafc.5c01661. 40267457

[291] Fang Y , Bai D , Qi J , Zeng Y , Liu M and Pan L , Glutathione transferases are linked to Penoxsulam resistance in *Echinochloa crus‐galli* (L.) P. Beauv. J Agric Food Chem 73:18220–18230 (2025). 10.1021/acs.jafc.5c04707. 40627566

[292] Evans AF Jr , O'Brien SR , Ma R , Hager AG , Riggins CW , Lambert KN *et al*., Biochemical characterization of metabolism‐based atrazine resistance in *Amaranthus tuberculatus* and identification of an expressed GST associated with resistance. Plant Biotechnol J 15:1238–1249 (2017). 10.1111/pbi.12711. PMC5595711 28218978

[293] Chen W , Bai D , Liao Y , Yu Q , Bai L and Pan L , *PfGSTF2* endows resistance to quizalofop‐p‐ethyl in *Polypogon fugax* by GSH conjugation. Plant Biotechnol J 23:216–231 (2025). 10.1111/pbi.14491. PMC11672750 39466674

[294] Zhan Y , Cao Z , Qi J , Luo Y , Hu L , Bai L *et al*., Fenoxaprop‐P‐ethyl resistance in *Alopecurus aequalis*: involvement of *GSTF13* in addition to Ile‐1781‐Leu mutation. Pestic Biochem Physiol 214:106614 (2025). 10.1016/j.pestbp.2025.106614. 40915850

[295] Dücker R , Lümmen P , Wolf T , Brabetz V and Beffa R , An intronless tau class glutathione transferase detoxifies several herbicides in flufenacet‐resistant ryegrass. Plant Physiol 196:1254–1267 (2024). 10.1093/plphys/kiae330. 38848314

[296] Liu X , Hou Z , Zhang Y , Merchant A , Zhong ME , Ma G *et al*., Cloning and functional characterization of a tau class glutathione transferase associated with haloxyfop‐P‐methyl resistance in *Digitaria sanguinalis* . Pest Manag Sci 79:3950–3958 (2023). 10.1002/ps.7588. 37248658

[297] Wang J , Du Y , Zhang L , Deng Y , Wang T , Xiong Z *et al*., Genomic insights into herbicide resistance evolution in allopolyploid *Monochoria korsakowii* . Plant J 126:e70890 (2026). 10.1111/tpj.70890. 42012804

[298] Shi H , Liu C , Wang S , Wang Y , Zhang Z , Shen Y *et al*., Gene mapping and identification of candidate genes associated with fomesafen herbicide tolerance in mung bean (*Vigna radiata* L.). Theor Appl Genet 139:81 (2026). 10.1007/s00122-026-05184-y. 41762234

[299] Feng T , Wang L , Lei T , Wu B , Wu L , Wang J *et al*., A natural glutathione S‐transferase gene *GSTU23* confers metabolic resistance to metamifop in *Echinochloa crus‐galli* . Int J Biol Macromol 277:134078 (2024). 10.1016/j.ijbiomac.2024.134078. 39038575

[300] Li Z , Jiang J , Song J , Bai L and Pan L , One glutathione *S*‐transferase gene, *GSTU163*, was involved in Mesosulfuron‐methyl resistance in *Alopecurus japonicus* . J Agric Food Chem 73:21829–21841 (2025). 10.1021/acs.jafc.5c08615. 40839404

[301] Qian H , Zeng Y , Zhao H , Fu M , Yu Q and Pan L , Diclofop‐methyl resistance conferred by *LrGSTU181* overexpression in *Lolium rigidum* . Pestic Biochem Physiol 216:106803 (2026). 10.1016/j.pestbp.2025.106803. 41350060

[302] Zeng Y , Qian H , Zhao H , Fu M , Pan L and Liu M , *LrGSTU181* contributes to glufosinate resistance in the multiple resistant *Lolium rigidum* populations. J Agric Food Chem 74:14322–14334 (2026). 10.1021/acs.jafc.5c17635. 42047551

[303] Yang F , Wang Y , Zhou W , Xue C , Dong F , Zheng Y *et al*., Glycosylation of glyphosate drives residue reduction and herbicide tolerance in rice. Proc Natl Acad Sci USA 123:e2516099123 (2026). 10.1073/pnas.2516099123. PMC12956818 41730107

[304] Li Z , Song J , Liu M , Tian J , Bai L and Pan L , Overexpressing of *GT8* confers resistance to fenoxaprop‐P‐ethyl in *Alopecurus japonicus* . Pest Manag Sci 81:3984–3994 (2025). 10.1002/ps.8764. 40084545

[305] Wang H , Liu Y , Sun P , Wang Y and Huang Q , *EcUGT79*‐like mediated metabolic resistance to auxin herbicide quinclorac in *Echinochloa crus‐galli* . J Agric Food Chem 73:22516–22529 (2025). 10.1021/acs.jafc.5c09734. 40862634

[306] Tian J , Bai D , He S , Cao Y , Liao Y , Wang J *et al*., Pro‐197‐his/ser mutation and the metabolic gene *DsUGT84A1*, synergistically confer resistance to tribenuron‐methyl in *Descurainia sophia* . Plant J 123:e70487 (2025). 10.1111/tpj.70487. 40988456

[307] Huang XX , Zhao SM , Zhang YY , Li YJ , Shen HN , Li X *et al*., A novel UDP‐glycosyltransferase 91C1 confers specific herbicide resistance through detoxification reaction in Arabidopsis. Plant Physiol Biochem 159:226–233 (2021). 10.1016/j.plaphy.2020.12.026. 33387851

[308] Maeda H , Murata K , Sakuma N , Takei S , Yamazaki A , Karim MR *et al*., A rice gene that confers broad‐spectrum resistance to β‐triketone herbicides. Science 365:393–396 (2019). 10.1126/science.aax0379. 31346065

